# Transformation of alkyl and aryl halides into alcohols, thiols and thiophenols

**DOI:** 10.1039/d6ra00558f

**Published:** 2026-07-02

**Authors:** Oscar Dunne, Emily A. Collins, Timothy P. O'Sullivan

**Affiliations:** a School of Chemistry, University College Cork Cork T12 YN60 Ireland tim.osullivan@ucc.ie; b School of Pharmacy, University College Cork Cork T12 YN60 Ireland; c Analytical and Biological Chemistry Research Facility, University College Cork Cork T12 YN60 Ireland

## Abstract

Alcohols and thiols are key functionalities which are found in a wide variety of both natural and synthetic bioactive compounds. Additionally, they provide access to an array of other major functional groups, such as aldehydes, ketones, ethers, disulfides and sulfonamides to name but a few. The direct conversion of alkyl and aryl halides constitutes a useful and efficient means of preparing alcohols, thiols and thiophenols. This review summarises the different approaches that have been reported in the literature up to 2026, and includes the application of modern synthetic methods, such as electrochemical and photochemical techniques. In each section, the main features of the different strategies are discussed, while their advantages and disadvantages are captured in high-level summary tables. Accordingly, this review should serve as a useful overview of the current state-of-the-art while also helping to identify gaps for future investigation.

## Abstract 2

A systematic review of the different approaches reported in the literature for the transformation of alkyl and aryl halides into alcohols, thiols and thiophenols.

## Graphical abstract

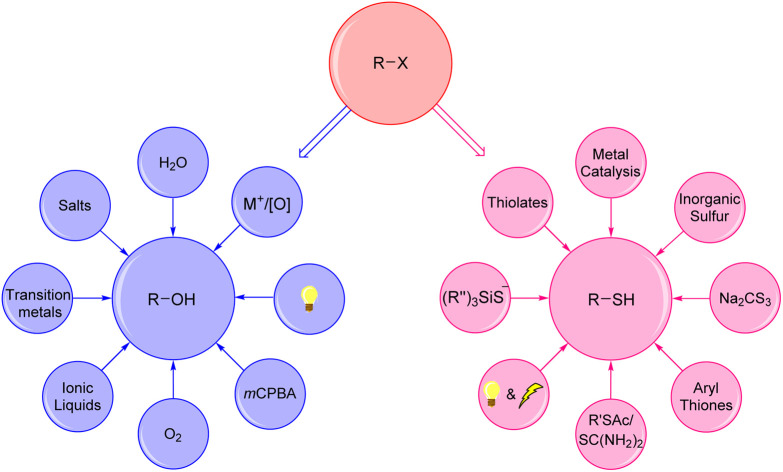

## 1. Introduction

**Fig. 1:**
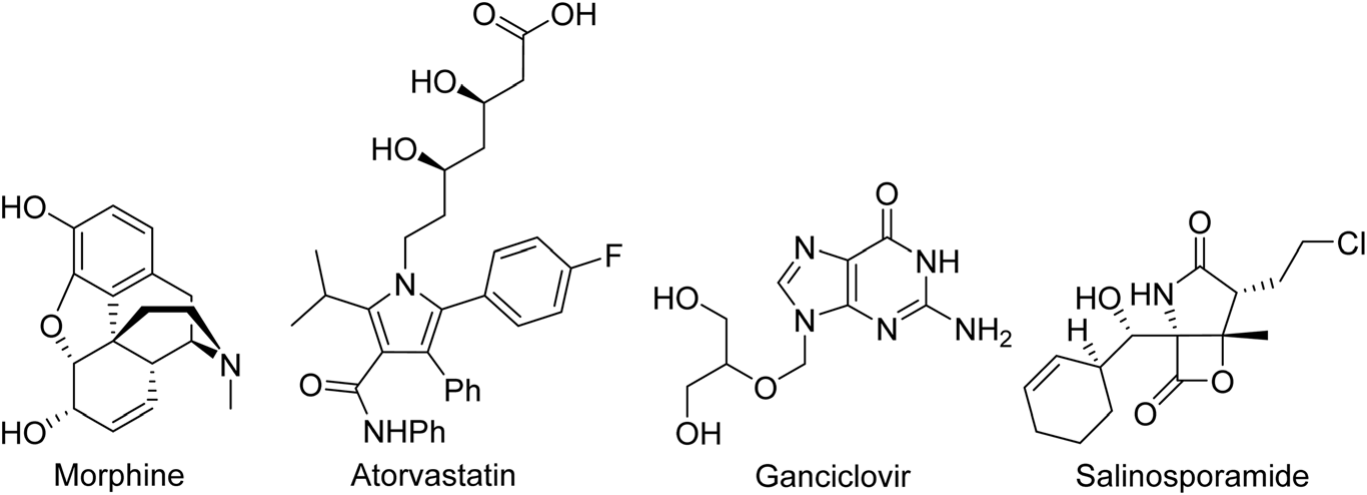
Therapeutic agents containing alcohol functionalities.

**Fig. 2:**
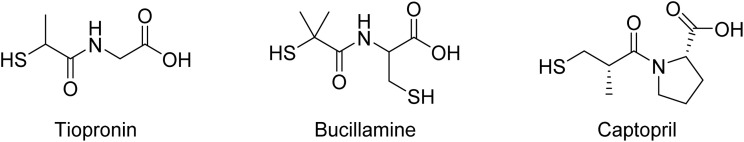
Therapeutic agents containing thiol functionalities.

Alcohols are present in a large number of pharmaceutical agents, including the analgesic morphine,^1^ the antihypercholesterolaemic atorvastatin^2^ and the antiviral agent ganciclovir (Fig. 1).^3^ Additionally, alcohols provide access to a wide range of other functionalities, such as aldehydes and carboxylic acids *via* oxidation,^4^ alkenes *via* dehydration^5^ and myriad others *via* Mitsunobu chemistry.^6^ Thiols are encountered less frequently than their oxygen counterparts but, nevertheless, constitute a valuable functional group. Thiols are found in pharmacologically active compounds such as tiopronin,^7^ used in the treatment of kidney stones, and the antirheumatic agent bucillamine^8^ (Fig. 2). Additionally, thiols can provide access to their corresponding disulfides *via* oxidation^9^ and are often utilised in the preparation of sulfonamides.^10–12^ Although the direct conversion of alkyl and aryl halides to alcohols and thiols remains somewhat underexploited, this strategy has been successfully applied to the synthesis of several medicinally relevant compounds, such as the anti-cancer agent salinosporamide (Fig. 1).^13^ Another example is that of the angiotensin-converting enzyme inhibitor captopril (Fig. 2), where the thiol group, which is essential for biological activity due to its interaction with the catalytic Zn^2+^ ion at the active site of the enzyme, is introduced *via* displacement of a halide.^14,15^ Furthermore, while a broad range of methods for obtaining alcohols and thiols from alkyl halides have been previously reported,^16,17^ no integrated review has yet been published specifically focussing on the transformation of alkyl and aryl halides to alcohols, thiols and thiophenols. A comprehensive survey of the literature to date has been conducted in this article, covering the preparation of aliphatic alcohols, aliphatic thiols and thiophenols.

Note that the synthesis of phenols is not presented here, as this has been dealt with extensively elsewhere.^18–23^

## 2. Aliphatic alcohols

This section focuses on the preparation of aliphatic alcohols from alkyl halides and is divided into the following subsections: 2.1 Reactions in water, 2.2 Silver catalysis, 2.3 Reactions with *m*CPBA, 2.4 Oxygen-mediated reactions, 2.5 Ionic liquid catalysis, 2.6 Mercury-assisted hydroxylations, 2.7 Reactions with salts, 2.8 Photochemical methods, and 2.9 Metallation-oxidation methods.

### 2.1 Reactions in water

The growing interest in ‘green chemistry’ and sustainability has led to an increased focus on water as a reaction medium.^24–26^ This includes reactions both “in water” (where the reactants form a homogenous mixture in the aqueous reaction medium) and “on water” (where the reactants form a heterogeneous mixture in the aqueous medium),^27,28^ with the latter approach being particularly useful for Diels–Alder cycloadditions^29^ and Claisen rearrangements.^30^ Aside from its ability to modulate reaction kinetics, water is an obviously attractive option given that it is a renewable, non-petroleum-based, non-toxic and non-flammable solvent as well as a potential oxygen source.^24^ The aqueous hydrolysis of alkyl halides is, however, a challenging process due to the poor nucleophilicity of water.^31^ In most cases, a more nucleophilic hydroxide ion is instead used to effect this transformation. To overcome the low reactivity of water, the addition of a polar, aprotic solvent can greatly enhance overall nucleophilicity through a solvent-mediated oxygen transfer mechanism. This feat has been achieved by a number of research groups,^32–34^ although elevated temperatures are typically required and the toxicity of these additional solvents may be a complicating factor.^32,34^

Hutchins and Taffer observed that a combination of either HMPA or NMP and water permits the facile conversion of alkyl halides to the alcohol products.^32^ Heating of primary alkyl halides in 15% v/v water in NMP or HMPA at 100 °C afforded the target alcohols in good to excellent yields (Table 1, entries 1–8). HMPA was the superior solvent, returning higher yields and requiring shorter reaction times (entries 1, 3, 5, 7, 9 *vs.* 2, 4, 6, 8, 10). Simple alkyl chlorides (entry 10) proved significantly less reactive than allyl chlorides which underwent rapid conversion in 96% yield (entry 13). Transformation of secondary alkyl halides also proceeded smoothly, albeit over longer reaction times (entries 14–18).

**Table 1:** Preparation of alcohols using HMPA/water or NMP/water

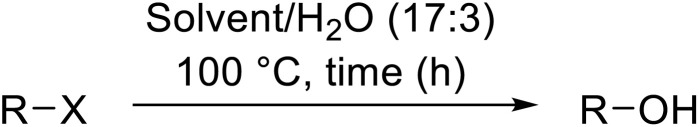					
Entry	R	X	Solvent	Time (h)	Yield
1	1-Dodecyl	I	HMPA	2.5	94%
2	1-Dodecyl	I	NMP	5.5	69%
3	1-Dodecyl	Br	HMPA	5.5	94%
4	1-Dodecyl	Br	NMP	15	75%
5	1-Octyl	I	HMPA	2.5	92%
6	1-Octyl	I	NMP	5.5	78%
7	1-Octyl	Br	HMPA	5.5	92%
8	1-Octyl	Br	NMP	15	87%
9	1-Octyl	Cl	HMPA	40	47%
10	1-Octyl	Cl	NMP	111	26%
11	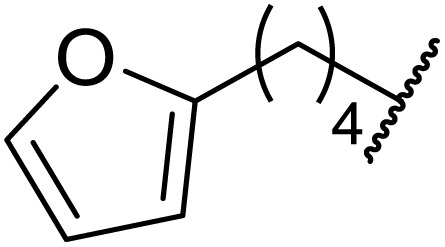	Br	HMPA	6	95%
12	PhCOCH_2_	Br	NMP	6	69%
13	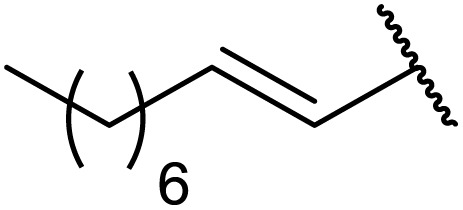	Cl	NMP	5	96%
14	Me(CH_2_)_5_CHMe	I	HMPA	8	75%
15	Me(CH_2_)_5_CHMe	Br	NMP	24	64%
16	Me(CH_2_)_7_CHMe	Br	NMP	24	78%
17	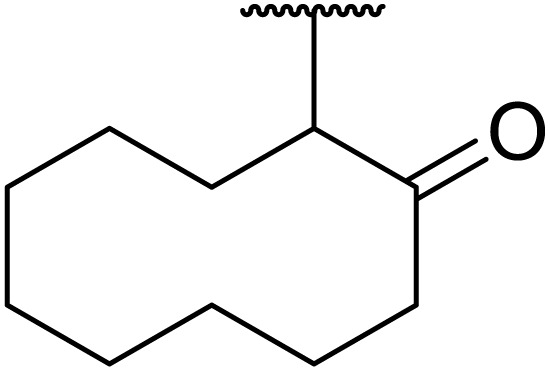	Br	NMP	50	70%
18	PhCOCH(Ph)	Br	NMP	70	70%

Liu and co-workers exploited a water/DMSO system to prepare various alcohols from their corresponding alkyl halides (Table 2).^33^ Following an initial screening study with benzyl bromide, a 1 : 2 v/v water/DMSO solution was found to be optimal. Primary benzylic halides provided good to high yields of the desired alcohols (entries 1–12). By contrast, primary aliphatic halides required the addition of catalytic amounts of caesium carbonate and tetrabutylammonium iodide, as well as higher reaction temperatures, to afford the corresponding alcohols in moderate yields (entries 13–17). Secondary alkyl bromides were converted in good to high yields (entries 18–20) while tertiary alkyl bromides required more forceful conditions to provide the target alcohols in moderate yields (entries 21–22).

**Table 2:** DMSO-mediated aqueous hydrolysis of alkyl halides^a^

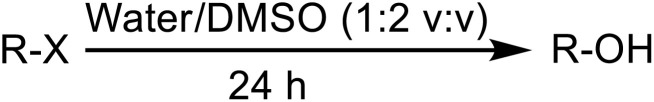					
Entry	R	X	Temp. (°C)	Method	Yield
1	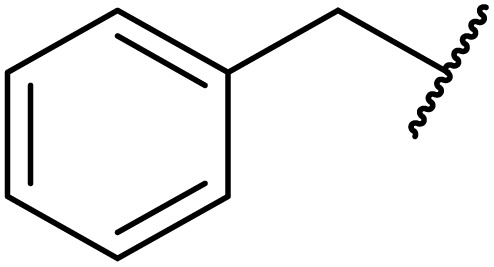	Br	50	A	83%
2^b^	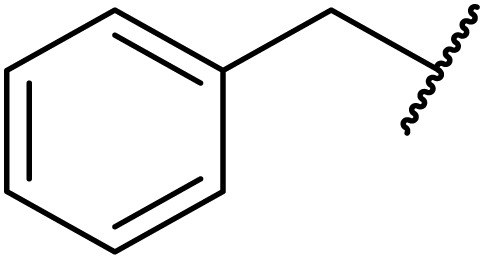	Cl	50	A	65%
3	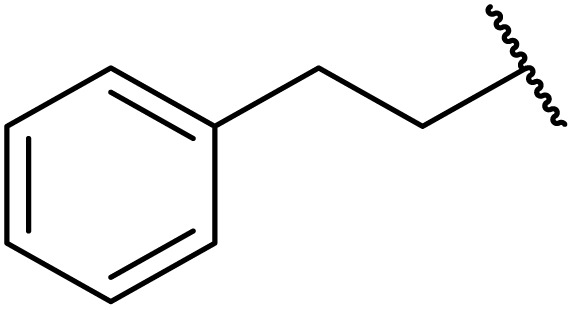	Br	100	B	78%
4	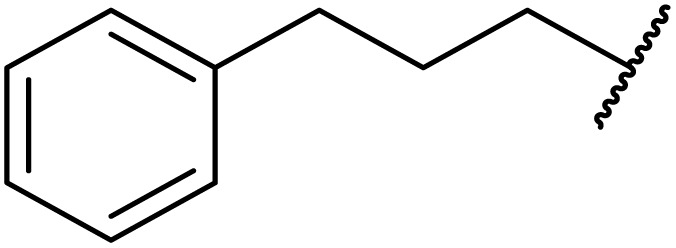	Br	100	B	65%
5	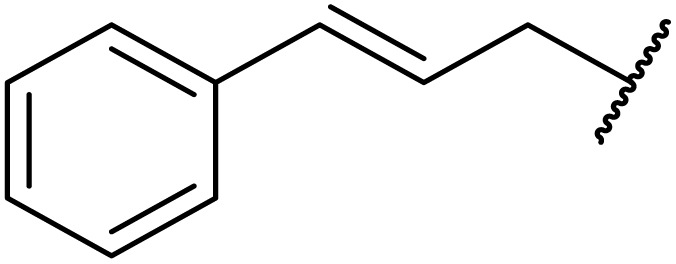	Br	50	A	35%
6	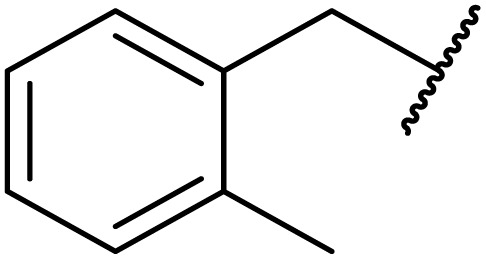	Br	50	A	79%
7	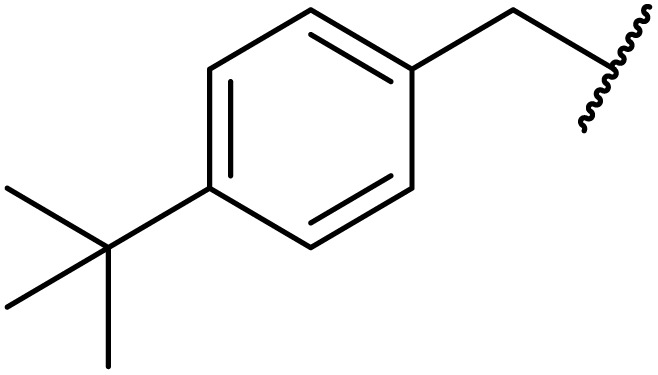	Br	50	A	64%
8	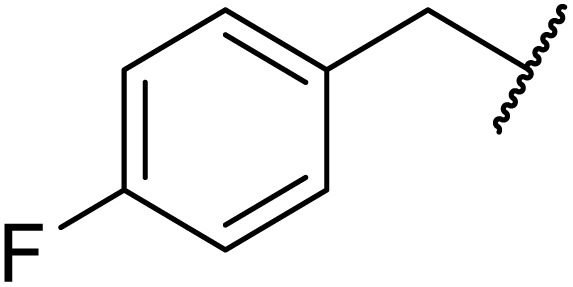	Br	50	A	87%
9	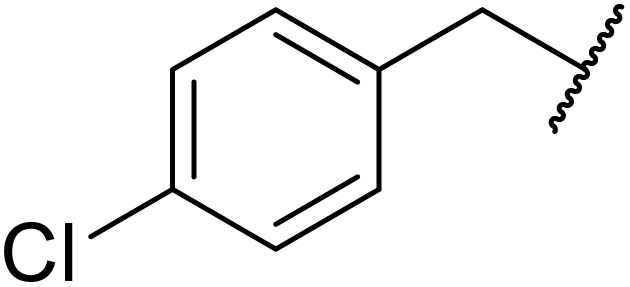	Br	50	A	69%
10	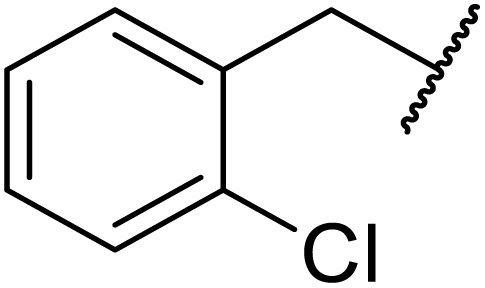	Br	50	A	77%
11	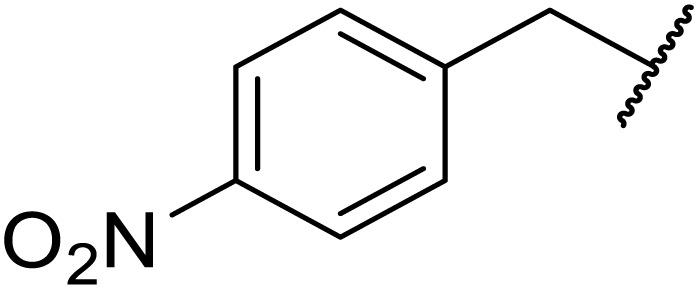	Br	50	A	81%
12	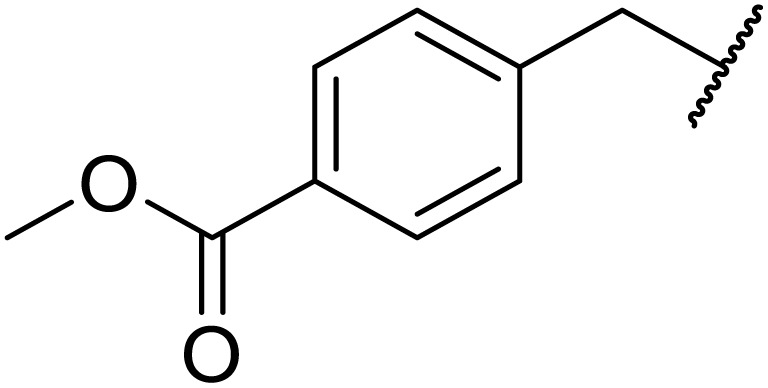	Br	50	A	62%
13	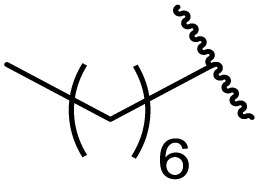	Br	100	B	58%
14	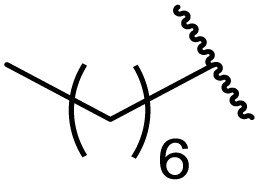	I	100	B	c56%
			100	B	52%
15	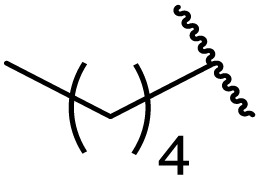	Br	100	B	51%
16	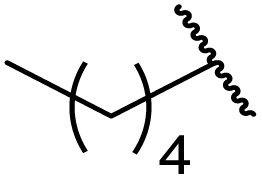	I	100	B	c55%
			100	B	48%
17	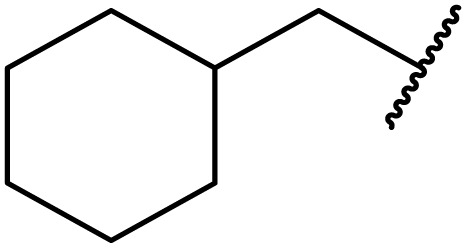	Br	100	B	21%
18	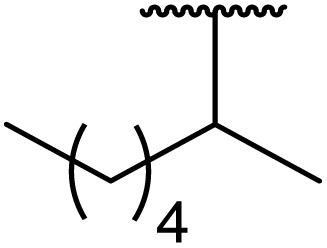	Br	100	B	29%
19	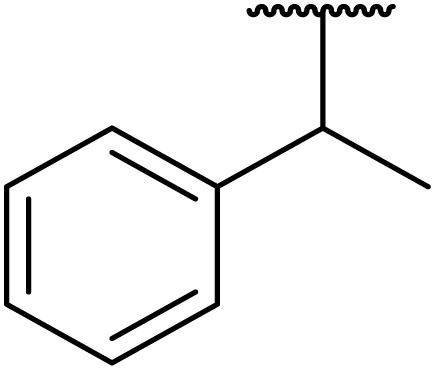	Br	50	A	60%
20	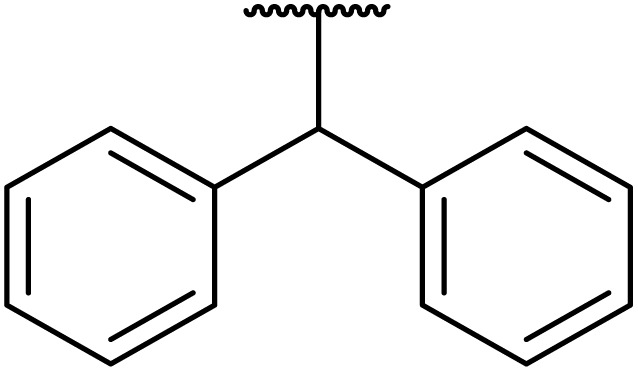	Br	50	A	80%
21	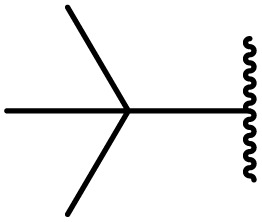	Br	50	A	22%
			100	B	56%
22	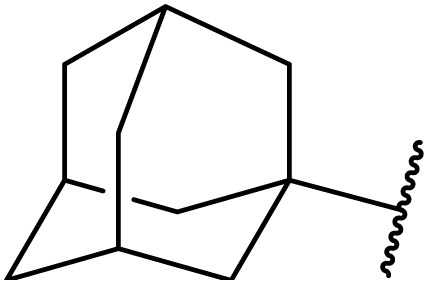	Br	50	A	62%
			100	B	78%

a Method A – no additives; Method B – addition of Cs_2_CO_3_ (20 mol%) and *n*Bu_4_NH_4_I (20 mol%).

b Reaction time 48 h.

c No *n*Bu_4_NH_4_I.

Shastri and co-workers similarly investigated a catalyst-free hydroxylation of alkyl halides using a polar, aprotic solvent system.^34^ A systematic comparison of several solvents found that water/DMSO and water/DMF performed the best, with the former slightly surpassing the latter. Primary aliphatic bromides were transformed to the corresponding alcohols in high yields (Table 3, entries 1–5). Primary alkyl bromides containing a coumarin backbone were highly reactive under these conditions and the target alcohols were isolated in yields above 88% (entries 6–14). Other primary alkyl halides (entries 15–18) were well tolerated with dihydroxylation observed for dihalogenated substrates (entries 16–17). Hydrolysis of more challenging secondary and tertiary alkyl halides similarly proceeded in high yields (entries 19–20). When reactions were conducted in water/acetic acid, only alcohol products were recovered and formation of the acetate adducts was not observed (entries 3–4, 6, 11–12, 14–17, 20).

**Table 3:** Hydrolysis of alkyl halides in polar aprotic water/solvent mixtures

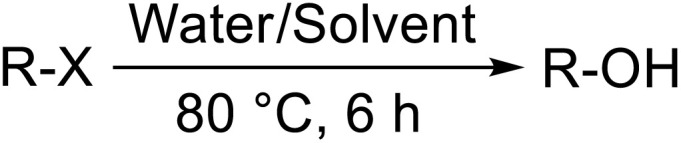						
Entry	R	X	Water/solvent ratio (v/v)	Yield (DMSO)	Yield (DMF)	Yield (acetic acid)
1	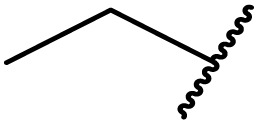	Br	7 : 3	88%	86%	—
2	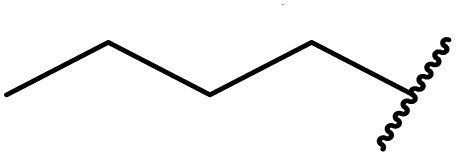	Br	7 : 3	80%	79%	—
3	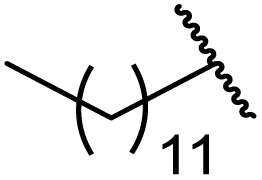	Br	7 : 3	85%	84%	a77%
4	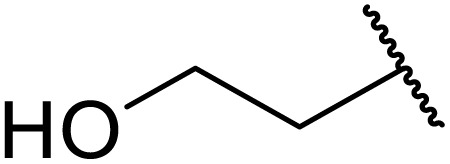	Br	7 : 3	82%	81%	a75%
5	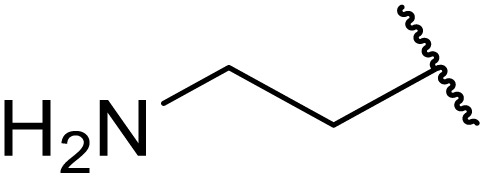	Br	7 : 3	84%	83%	—
6	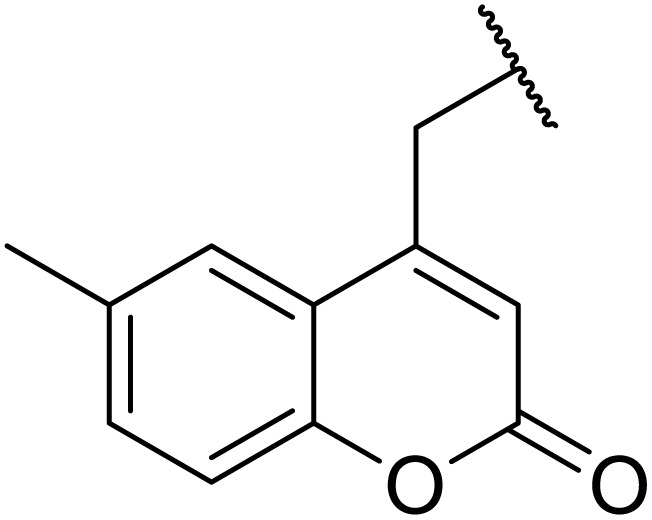	Br	8 : 2	95%	94%	93%
7	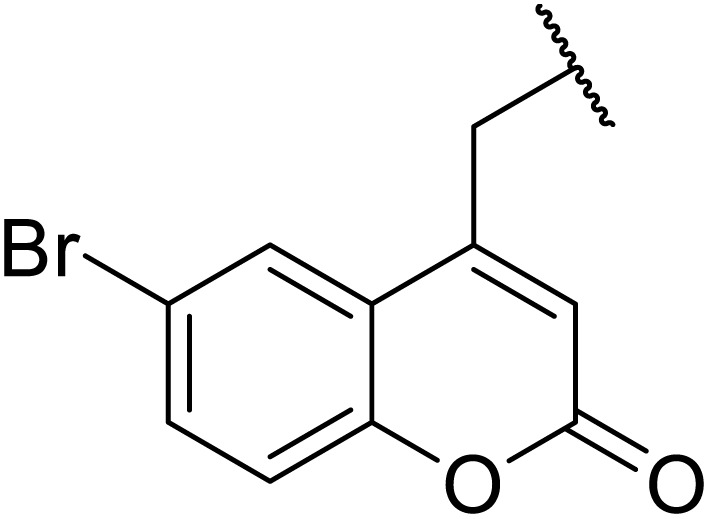	Br	8 : 2	93%	92%	—
8	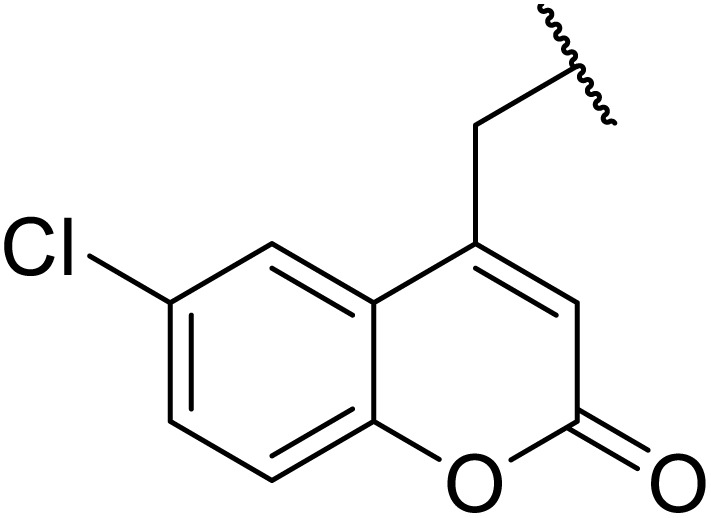	Br	8 : 2	91%	89%	—
9	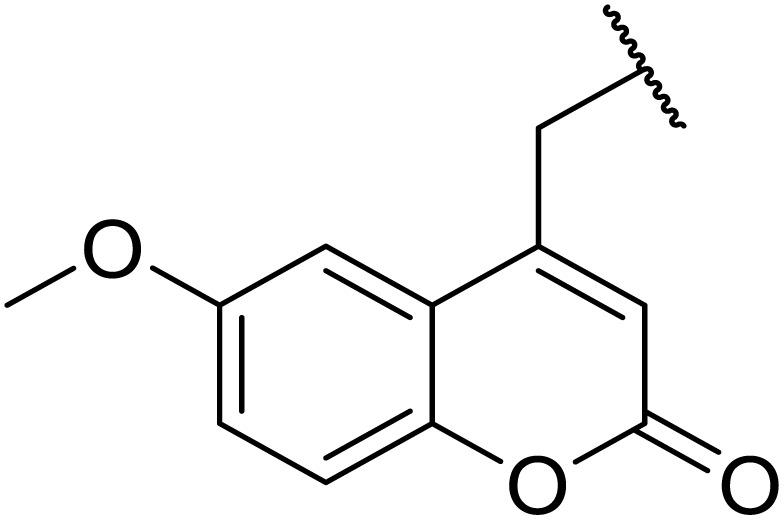	Br	8 : 2	92%	90%	—
10	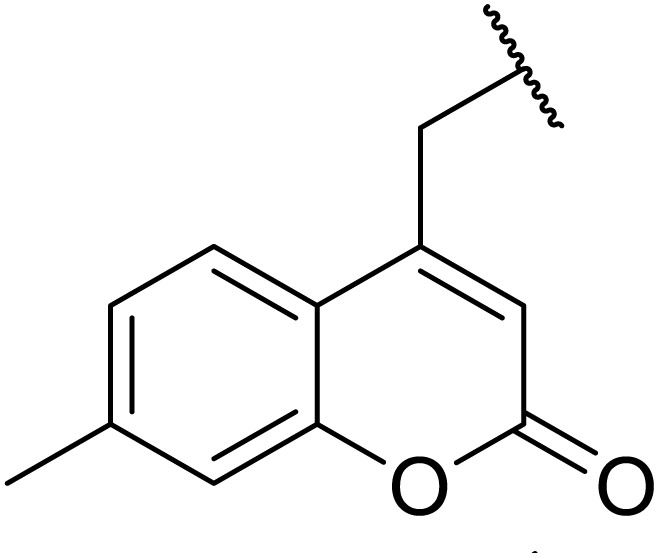	Br	8 : 2	94%	93%	—
11	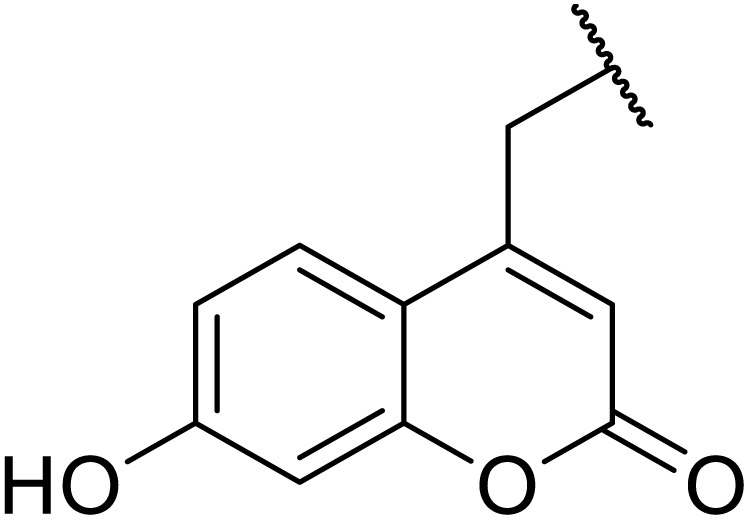	Br	8 : 2	90%	88%	88%
12	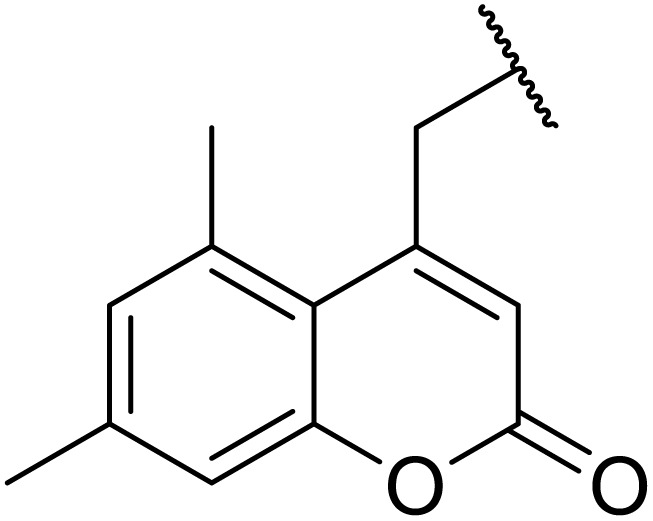	Br	8 : 2	93%	92%	91%
13	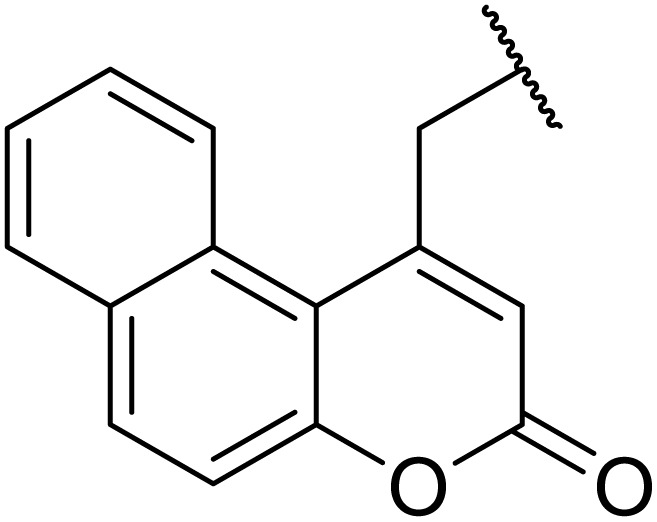	Br	8 : 2	91%	89%	—
14	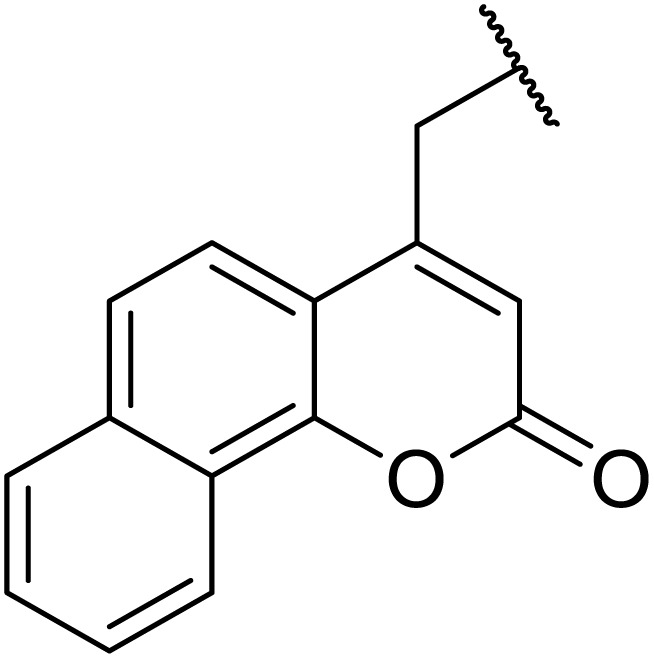	Br	8 : 2	92%	90%	90%
15	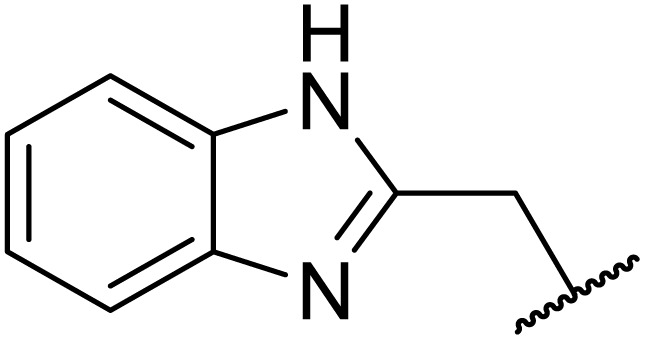	Cl	8 : 2	77%	75%	73%
16	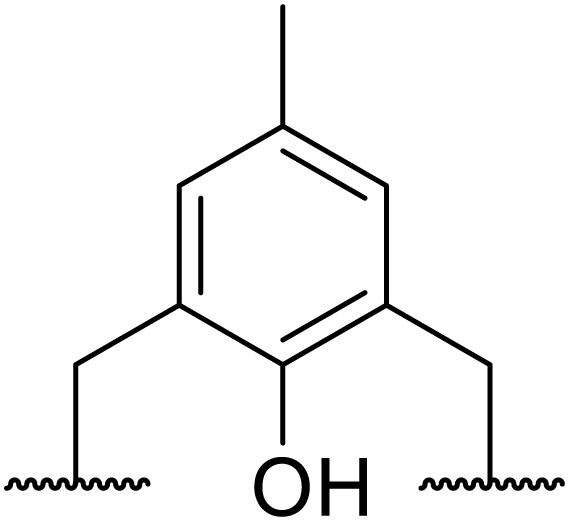	Cl	8 : 2	73%	70%	69%
17	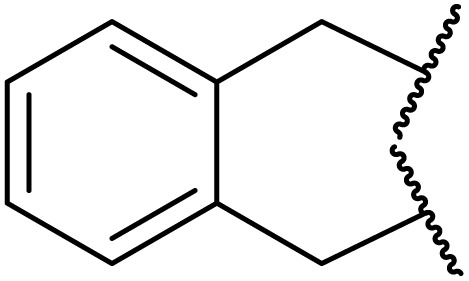	Br	8 : 2	79%	76%	75%
18	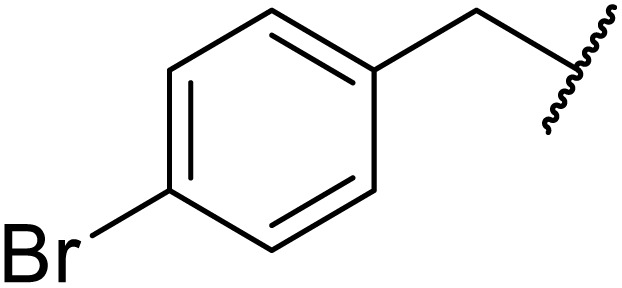	Cl	8 : 2	75%	73%	—
19	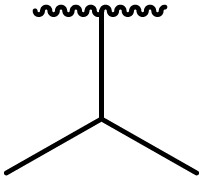	Br	7 : 3	77%	76%	—
20	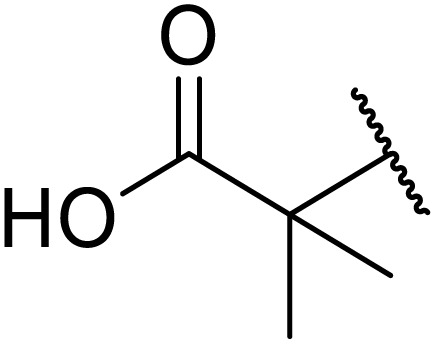	Br	7 : 3	78%	78%	a76%

a Water/solvent ratio (v/v) (8 : 2).

A two-step preparation of alcohols from alkyl halides was developed by Lee and co-workers which avoids elimination side reactions.^35^ The oxygen functionality was installed *via* acetoxylation using either potassium acetate or tetraethylammonium acetate, which was followed by hydrolysis with potassium hydroxide to produce the final alcohol (Table 4). Primary alkyl iodides, bromides, chlorides and mesylates could be converted to their corresponding alcohols in excellent yields of 84–98% (entries 1–5), although the less reactive chlorides and mesylates necessitated higher temperatures or extended reaction times (entries 3–4). Secondary mesylates were successfully hydrolysed with no evidence for the formation of elimination products (entries 6–7) or cleavage of a Boc protecting group (entry 7). Higher yields and shorter reaction times were recorded when potassium acetate was replaced with tetraethylammonium acetate, likely due to the increased solubility and nucleophilicity of the latter (entries 6–7). A similar alkaline hydrolysis method has been exploited in the stereospecific synthesis of 16α-hydroxy-17-oxo steroids.^36^

**Table 4:** Two-step hydrolysis of alkyl halides

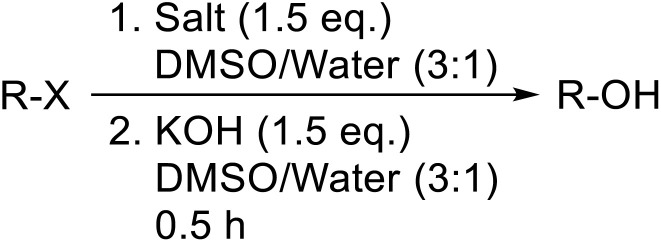						
Entry	R	X	Salt	Temp. (°C)	Time (h)	Yield
1	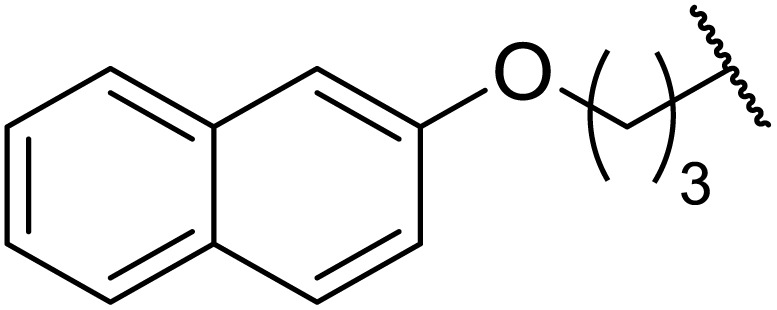	I	KOAc	r.t.	1.5/0.5	98%
2	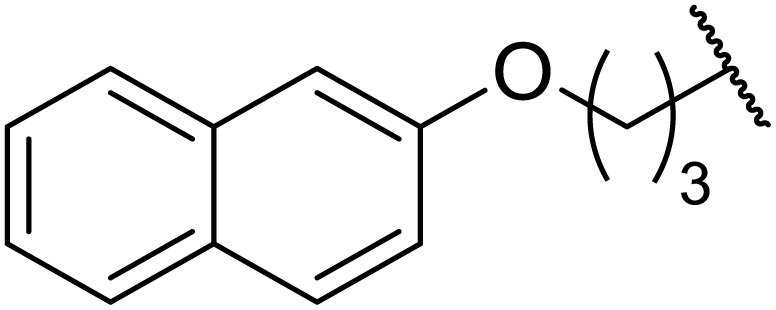	Br	KOAc	r.t.	2.5/0.5	96%
3	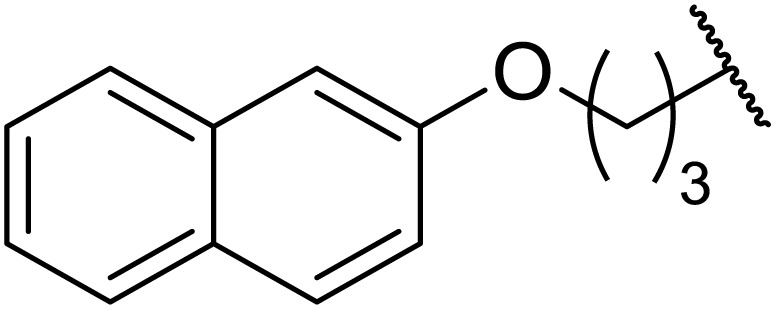	Cl	TEAOAc	50	7.5/0.5	92%
4	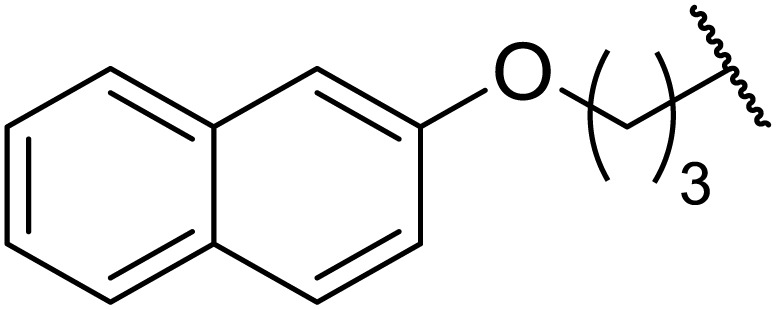	OMs	KOAc	40	2.5/0.5	94%
5	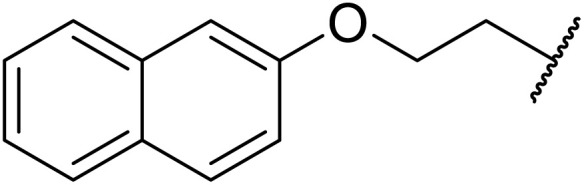	Br	KOAc	r.t.	3.5/0.5	84%
6	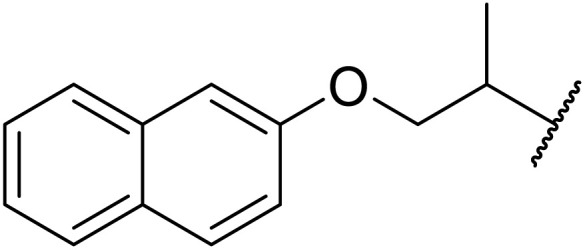	OMs	KOAc	70	5.0/0.5	89%
		OMs	TEAOAc	70	3.0/0.5	92%
7	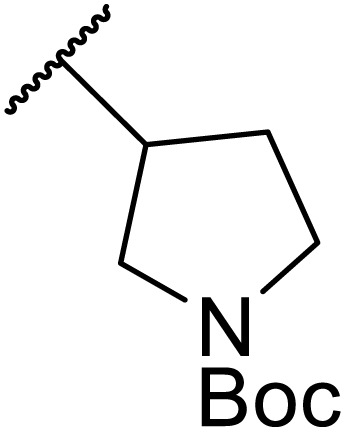	OMs	KOAc	100	9.5/0.5	65%
		OMs	TEAOAc	80	3.5/0.5	96%

The hydrolysis of alkyl halides in water/DMSO has been successfully applied to the synthesis of long chain ether diols for use in the treatment of metabolic syndrome (Table 5).^37^ Primary alkyl bromides with varying chain lengths and substituents were hydrolysed in yields of 38–99% (entries 1–3).

**Table 5:** Water/DMSO-mediated synthesis of long chain ether diols

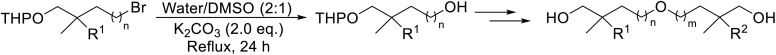			
Entry	*n*	R^1^	Yield of ROH
1	4	CH_3_	99%
2	5	CH_3_	83%
3	4	Ph	38%

**Scheme 1:**
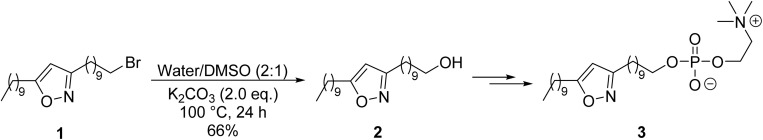
Hydrolysis of alkyl bromides in water/DMSO.

In an effort to synthesise phospholipids with potential antiparasitic activity, Magoulas and colleagues utilised a water/DMSO-mediated hydrolysis of a primary alkyl bromide (Scheme 1).^38^ After 24 hours at 100 °C, intermediate 1 was converted in 66% yield to alcohol 2 which was subsequently carried through to target phospholipid derivative 3.

### 2.2 Silver catalysis

Compared to other transition metals, silver-mediated catalysis has been traditionally associated with low catalytic efficiency, and this has limited its application in synthetic organic chemistry until relatively recently.^39^ Advances in technologies, however, such as the development of metal nanoparticles, has sparked renewed interest in this field.^40–42^ Silver has been long used to catalyse the conversion of alkyl halides to alcohols, due to its high affinity for halides.^43^ The procedures described below typically proceed *via* formation of a hydrolytically-unstable oxygenated intermediate following nucleophilic displacement of the halide by a nucleophilic oxygen source. Subsequent aqueous hydrolysis affords the target alcohol.^44,45^ These reactions generally provide products in high yields,^44,45^ are compatible with alkyl bromides and chlorides,^44,45^ and are suitable for complex, polyfunctional substrates.^45^ Several groups have also used silver salts in a polar solvent/water reaction medium and obtained the target alcohols in a single step.^46–48^ A drawback with many of these approaches centres around the high catalyst loading (*e.g.* stoichiometric or even superstoichiometric). Finally, while silver is not particularly toxic in its elemental form, compounds such as silver nitrate are highly toxic to aquatic life.^40,49^

Gingras *et al.* employed silver salts (*e.g.* silver nitrate, silver tosylate and silver oxide) for the hydrolysis of alkyl and cyclic halides in the presence of bis(tributyltin) oxide (Table 6).^44^ In this scenario, bis(tributyltin) oxide acts as the oxygen nucleophile where the tin atom increases the nucleophilicity of the attached oxygen atom *via* electron channelling.^50^ DMF proved crucial as the reaction medium due to its strong coordinating ability. In general, the yields obtained from primary alkyl halides ranged from moderate to excellent (entries 1–13). In the absence of the tin oxide, yields were significantly lower (entry 4 *vs.* 5). Primary alkyl chlorides failed to react under these conditions (entry 6) unless first converted to the corresponding alkyl iodides *via* a Finkelstein reaction (entries 7 and 8). Unlike other substitution reactions involving alkyl halides, little, if any, elimination side products were observed. For example, 2-iodoethylbenzene was converted without the formation of unwanted styrene (entry 9). This methodology was also compatible with ester-containing substrates (entries 11–13). By contrast, transformation of secondary and tertiary alkyl halides proved challenging with elimination constituting the major reaction pathway (entries 14 and 15). Silver nitrate has been similarly employed in the preparation of chiral catalysts^51^ while silver oxide was likewise utilised in the synthesis of congested adamanylideneadamantanes.^52^

**Table 6:** Ag(i)-catalysed synthesis of alcohols from halides in the presence of (Bu_3_Sn)_2_O

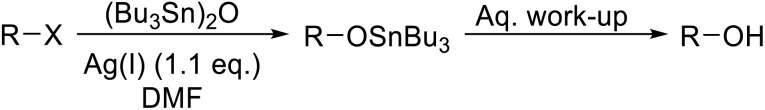							
Entry	R	X	Organotin eq.	Silver salt (eq.)	Temp. (°C)	Time (h)	Yield
1	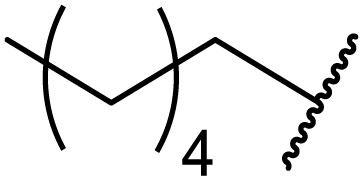	I	2.2	AgTos (2.0)	20	2	84%
2	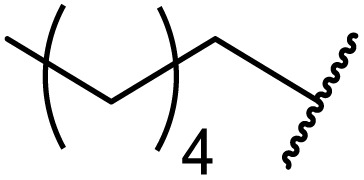	Br	2.2	AgTos (1.1)	90	22	74%
3	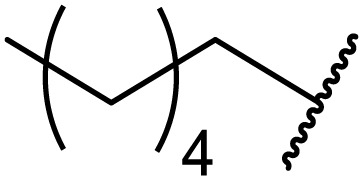	Br	2.2	Ag_2_O (1.1)	90	14	71%
4	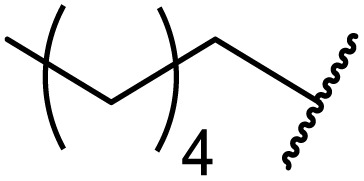	Br	0	Ag_2_O (1.1)	90	22	12%
5	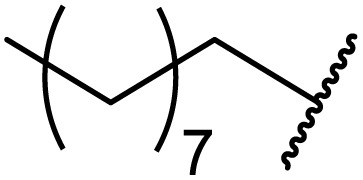	Br	2.2	AgTos (1.1)	80	23	47%
6	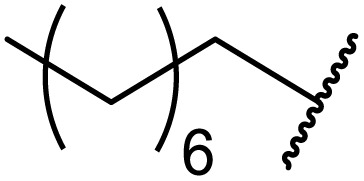	Cl	2.2	AgNO_3_ (1.1)	125	36	—
7^a^	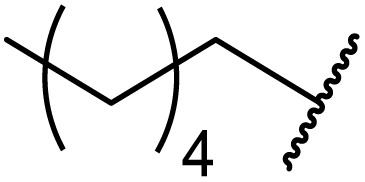	Cl	2.2	AgTos (1.1)	20	2	74%
8^a^	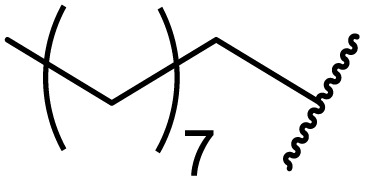	Cl	2.2	AgNO_3_ (1.1)	20	3	50%
9	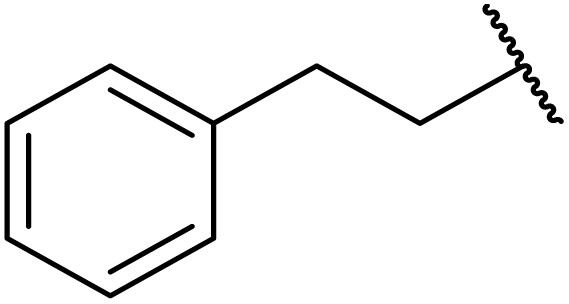	I	2.2	AgNO_3_ (2.0)	20	0.5	70%
10^a^	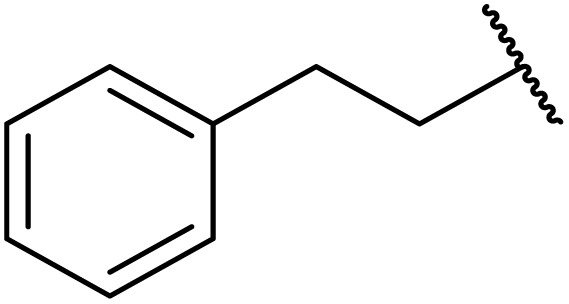	Br	2.2	AgNO_3_ (1.1)	20	0.5	63%
11	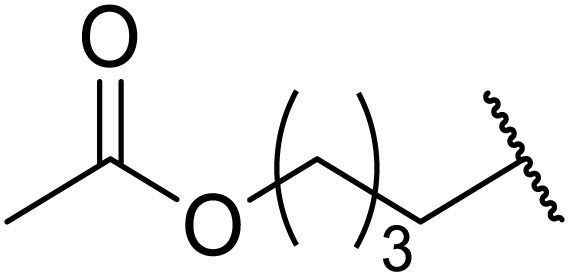	Br	2.2	AgNO_3_ (1.1)	90	24	28%
12^a^	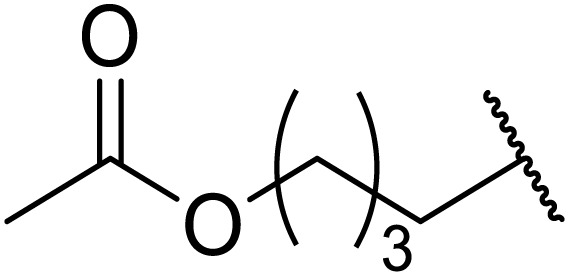	Br	2.2	AgNO_3_ (1.1)	20	5	91%
13	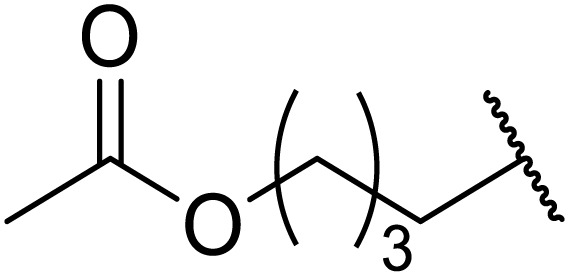	I	2.0	AgTos (1.3)	20	5.5	96%
14	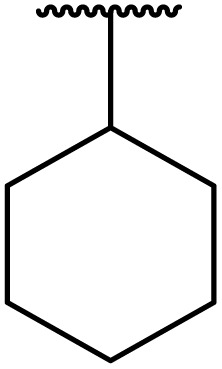	I	2.2	AgTos (1.1)	75	28	0–5%
15	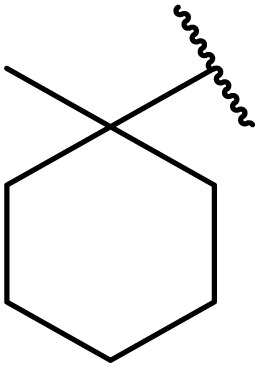	Br	2.2	AgNO_3_ (1.1)	20	4	0–5%

a Converted to alkyl iodide *via* Finkelstein reaction.

The conversion of alkyl halides to alcohols in DMF under silver catalysis has been reported by Abad and colleagues.^45^ This transformation proceeds over two steps, namely the silver-catalysed substitution of the alkyl halide by DMF to generate a formate ester, followed by acidic or basic hydrolysis (Table 7). Silver tetrafluoroborate resulted in rapid formyloxylation whereas reactions with silver acetate proved much more sluggish. Switching to silver nitrate saw significant formation of the nitrate side product. The use of nitromethane as a solvent was found to substantially increase the overall reaction rate. Activated alkyl chlorides gave high yields of the desired alcohols (entry 1). Unactivated alkyl chlorides reacted slowly at room temperature and required heating to 75 °C for full conversion (entry 2). By contrast, alkyl bromides reacted readily at room temperature (entry 3). Sterically hindered alkyl bromides and iodides were transformed in good yields (entries 4 and 5). As expected, primary alkyl iodides reacted more rapidly than the corresponding bromides (entry 4 *vs.* 5). High yields of alcohol products were obtained from primary benzylic bromides (entry 6). This methodology was also compatible with secondary alkyl halides which reacted *via* formation of stabilised carbocation intermediates (entries 7–10). By contrast, the formyloxylation of bromocyclododecane was unsuccessful, leading to a mixture of *E*/*Z* cyclododecenes (entry 11). Tertiary alkyl halides reacted *via* an elimination pathway to furnish solely elimination products (entry 12).

**Table 7:** AgBF_4_-catalysed conversion of halides to alcohols^a^

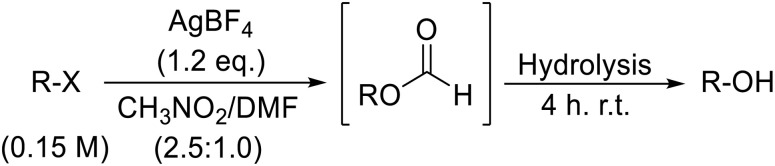						
Entry	R–X	Temp. (°C)	Time (h)	Hydrolysis conditions	R–OH	Yield
1	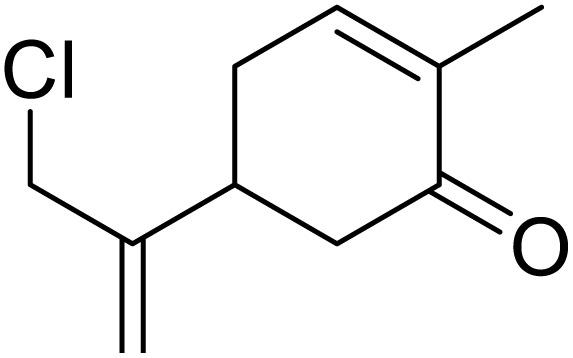	r.t.	24	A, B	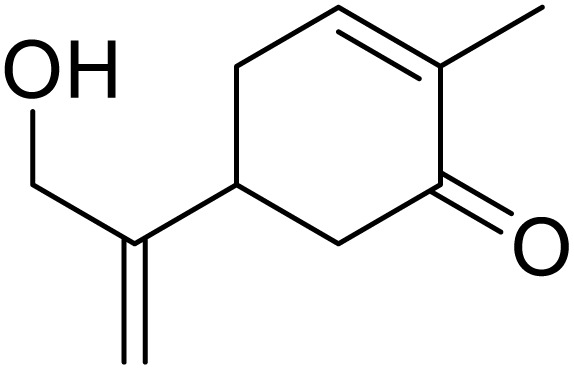	86%
2	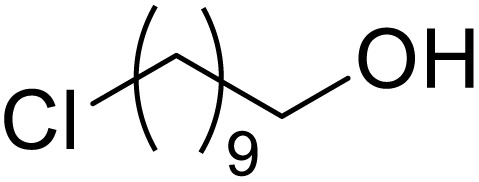	75	144	A, B	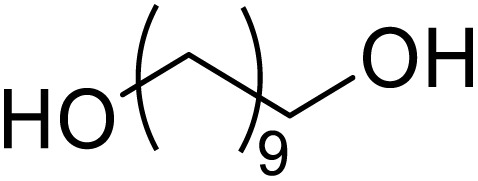	89%
3	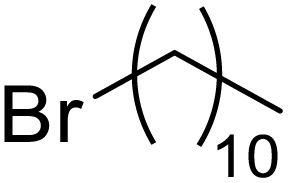	r.t.	24	A, B	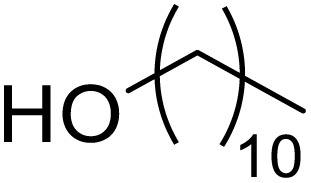	85%
4	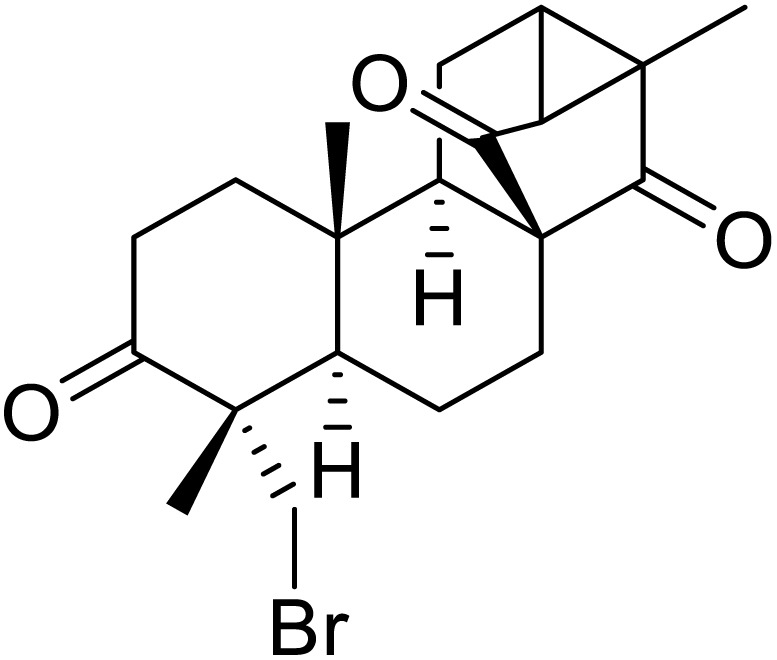	60	12	C	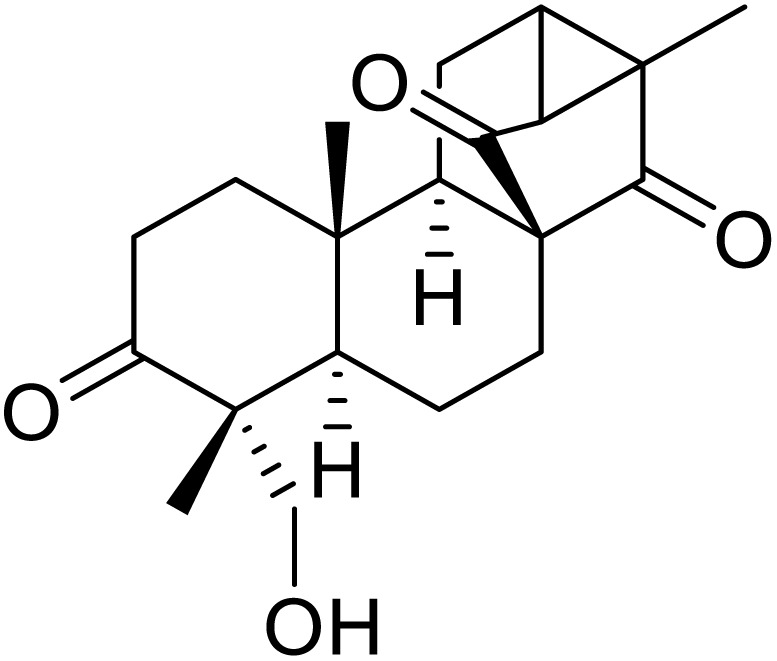	82%
5	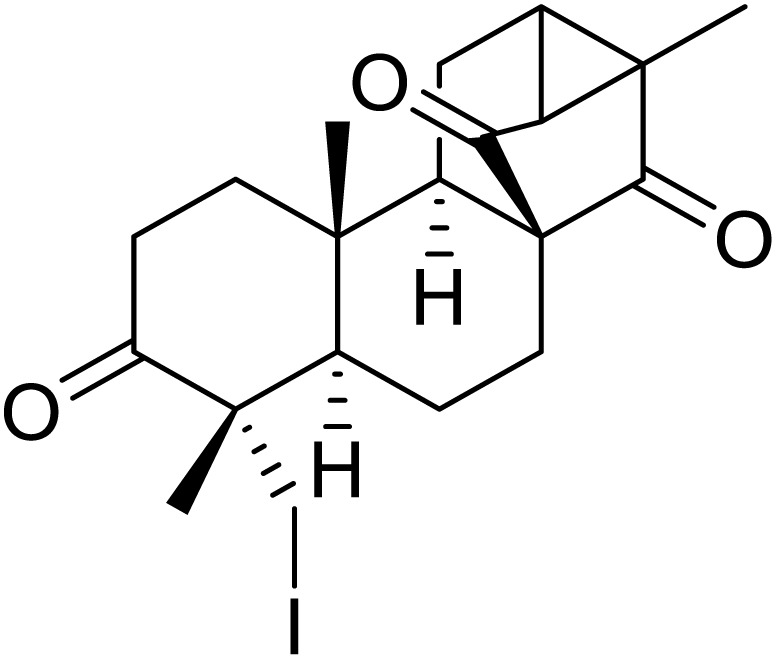	40	12	C	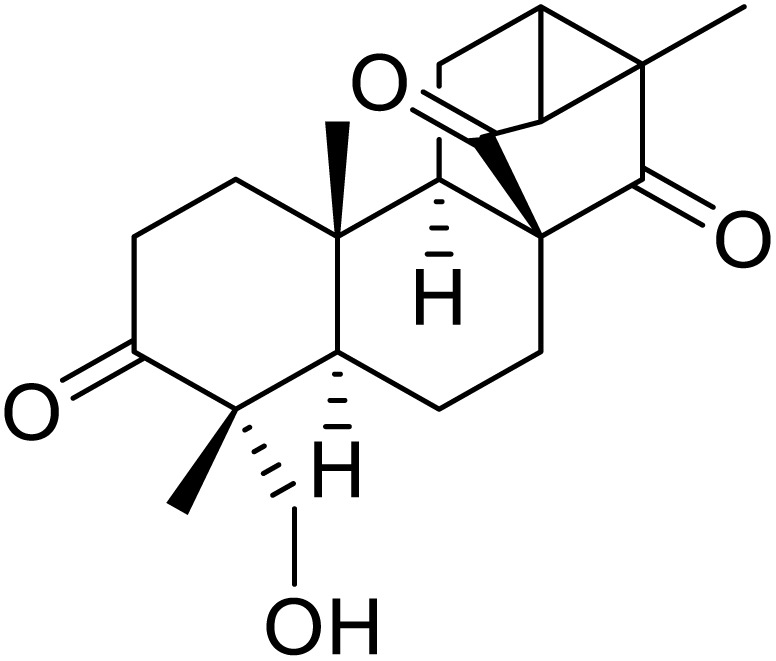	85%
6	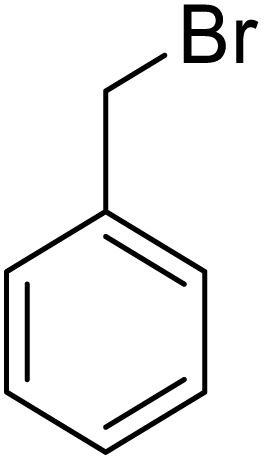	r.t.	12	A	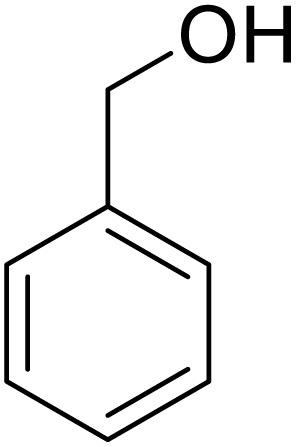	90%
7	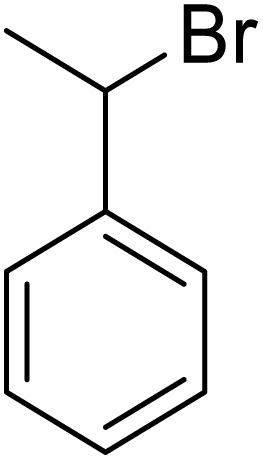	r.t.	1.5	A, B	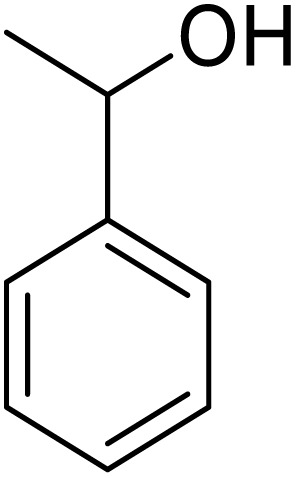	95%
8	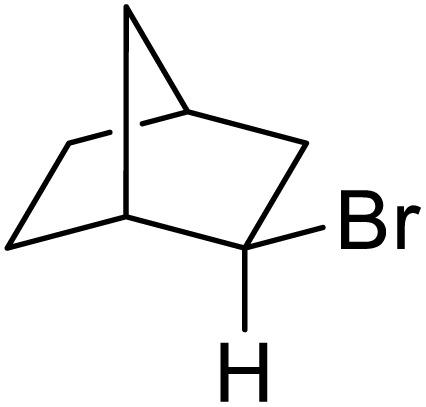	0	0.5	A, B	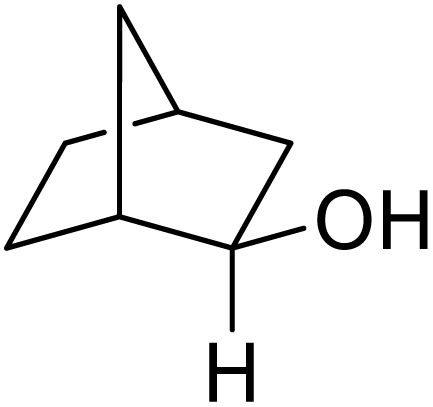	87%
9	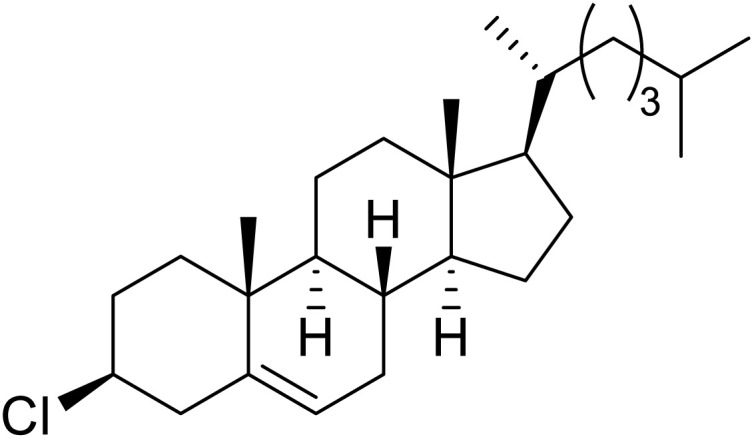	40	12	A	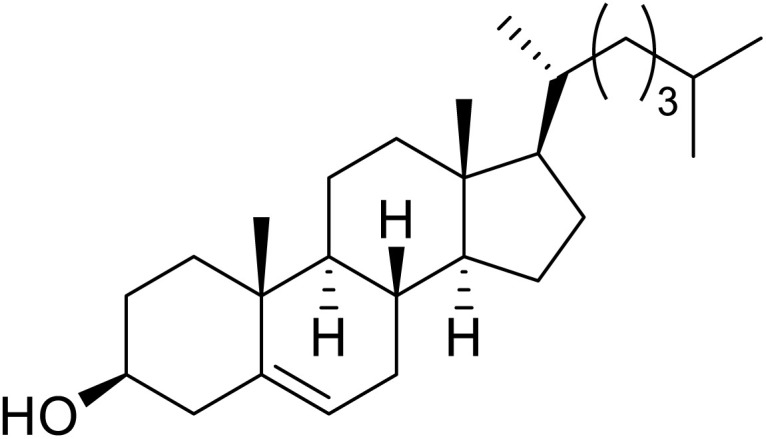	89%
10	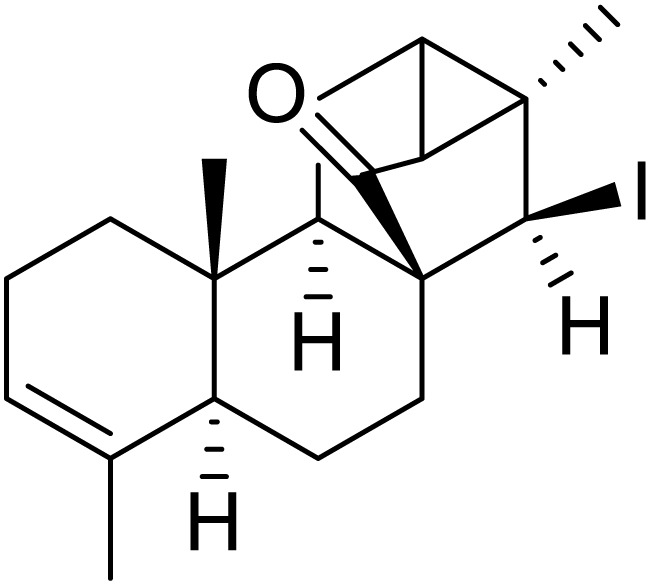	r.t.	1	A	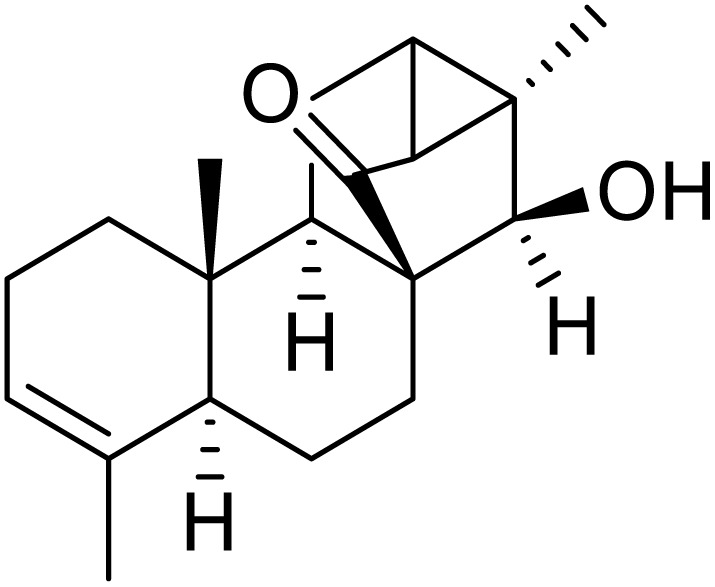	85%
11	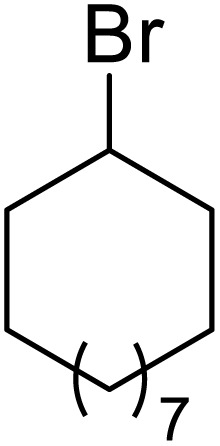	r.t.	12	—	—	—
12	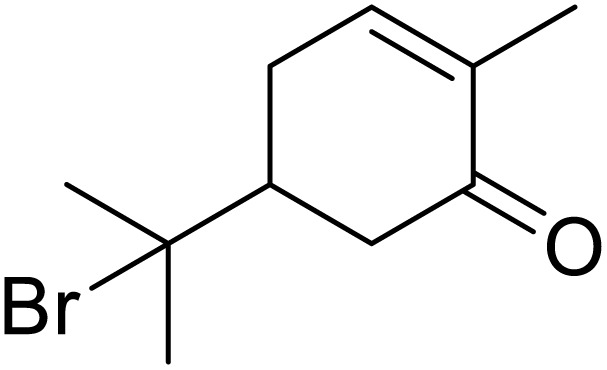	−10	0.25	—	—	—

a A: K_2_CO_3_–MeOH, B: (CO_2_H)_2_–MeOH, C: HCl–MeOH.

**Scheme 2:**
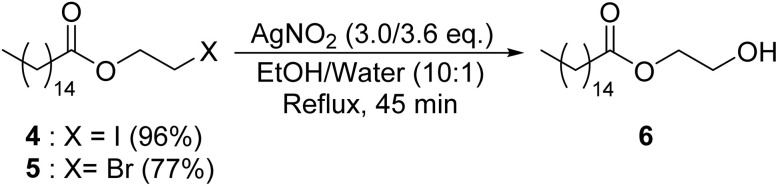
Hydrolysis of haloethyl esters using silver nitrite.

The generation of large amounts of unwanted diesters when preparing glycol monoesters of fatty acids, even in the presence of a large excess of glycol, has been noted in the literature.^53^ Bevan and colleagues circumvented this problem by conversion of 2-haloethyl esters to the corresponding alcohols (Scheme 2).^46^ Treatment of 2-iodoethyl palmitate (4) with 3.0 equivalents of silver nitrite under reflux afforded 6 in 96% yield. Conversion of 2-bromoethyl palmitate (5) was similarly successful, albeit in a lower (77%) yield and required 3.6 equivalents of silver nitrite.

**Scheme 3:**
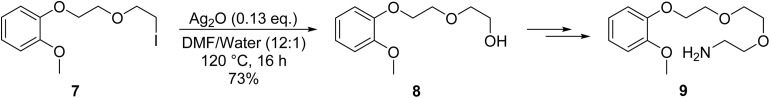
Preparation of guaiacol-derived aminoethers.

The antitussive and anaesthetic properties of aminoether analogues of guaiacol have been investigated by Carenini and co-workers.^47^ A key step in the synthesis of these molecules involves the conversion of alkyl iodide 7 to alcohol 8 in the presence of silver oxide (Scheme 3). The reaction was conducted in a 12 : 1 mixture of DMF/water and heated to 120 °C to afford target alcohol 8 in 73% yield. A notable feature of this work is the relatively low catalyst loading.

**Scheme 4:**
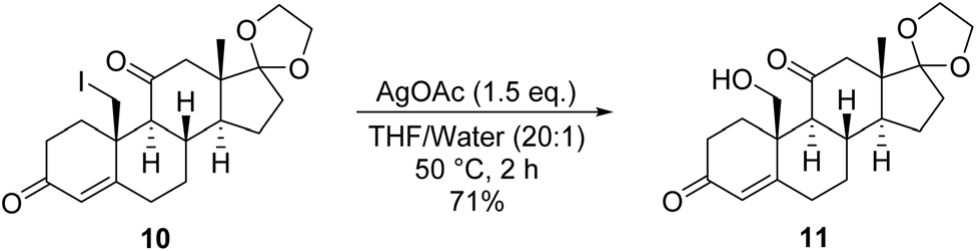
Silver acetate-mediated hydrolysis.

**Scheme 5:**
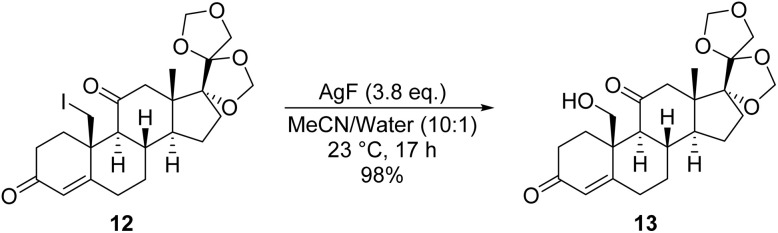
Silver fluoride-mediated hydrolysis.

Ouabagenin is a complex cardiotonic which may have potential application in the treatment of heart failure.^54^ In their development of a total synthesis of ouabagenin and related analogues, Baran *et al.* investigated the conversion of alkyl iodide 10 to primary alcohol 11 (Scheme 4).^48^ Hydrolysis of 10 was accomplished using 1.5 equivalents of silver acetate in THF/water at 50 °C. In the same paper, the hydrolysis of alkyl iodide 12 was achieved using silver fluoride to afford alcohol 13 in 98% yield, although the rationale for switching from silver acetate to silver fluoride is not provided (Scheme 5).

### 2.3 Reactions with *m*CPBA

*meta*-Chloroperoxybenzoic acid (*m*CPBA) is a strong oxidising agent which is widely used in transformations such as the oxidation of alkenes, carbonyl compounds, *N*-heterocycles, *etc.*^55^ In addition, it has found use in named reactions *e.g.* Baeyer–Villiger oxidation,^56^ Meisenheimer rearrangement,^57^ Cope elimination^58^ and Rubottom oxidation,^59^ to name a few. Its widespread use has been attributed to its versatile oxidising ability and relative ease of handling.^55,60^ Beyond 85% purity, however, it should be handled with caution due to its shock-sensitive and potentially explosive nature.^61^ A less common use of *m*CPBA involves the oxidative displacement of iodine from alkyl iodides to afford the corresponding alcohols.^62^ Other oxidising agents such as ozone, hydrogen peroxide and periodate are ineffective at performing this transformation.^63^ The reagent is somewhat limited by substrate scope, and is only capable of converting alkyl iodides to alcohols and not less reactive halides. Furthermore, stereochemical retention of secondary iodides is dependent on the nature of adjacent functional groups^62^ and the formation of *m*-chlorobenzoate, epoxide and/or ketone side products may be difficult to eliminate.^62,64^ An excess of *m*CPBA is typically required for full conversion. Contrastingly, this approach does offer expedient reaction times and excellent yields for simple substrates. Additionally, introducing water to the system can help ameliorate side-product formation.^64^

The oxidative displacement of iodide from primary alkyl iodides and vicinal iodocyclohexanes was examined by Cambie and colleagues in both dichloromethane and *t*-BuOH/water (Table 8).^62^ Unsubstituted primary alkyl iodides gave the desired alcohols in quantitative yields (entries 1–3). Neighbouring substituents had a significant impact on the reaction outcome (entries 4–7). While retention of configuration was observed in most cases, stereochemical inversion was recorded for the acetate and trifluoroacetate substrates (entries 4 and 5). The lower yield for the methoxy-substituted substrate was partly due to the formation of the *meta*-chlorobenzoate ester side product (entry 7). The same was true for vicinal thiocyanate substrates (entries 8 and 9). When these reactions were instead performed in *t*-BuOH/water, the yields were generally higher, albeit at the cost of longer reaction times and higher oxidant loading (entries 5–10). In particular, the use of *t*-BuOH/water eliminated the formation of the *meta*-chlorobenzoate side products leading to significantly improved yields in certain cases (entries 6–9). Complete inversion was observed for reactions involving secondary alkyl iodides with comparable outcomes in dichloromethane or *t*-BuOH/water (entry 10). Reactions of bromo-substituted iodocyclohexanes conducted in *t*-BuOH/water afforded the desired alcohol exclusively (entry 11) whereas the corresponding chlorinated substrate produced a mixture of alcohol and epoxide products (entry 12). Similar results were reported by Reich and Peake in related studies.^63^

**Table 8:** Oxidative displacement of iodine from alkyl halides using *m*CPBA

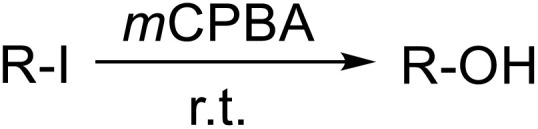						
Entry	R–I	*m*CPBA (eq.)	Time (min)	Solvent	Product	Yield
1	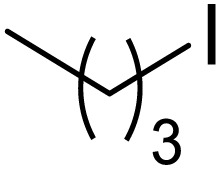	2.2	5	CH_2_Cl_2_	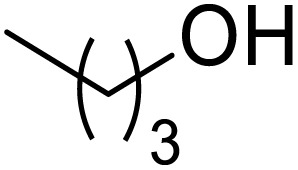	95%
2	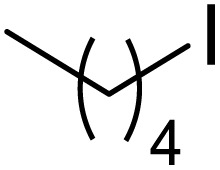	2.2	5	CH_2_Cl_2_	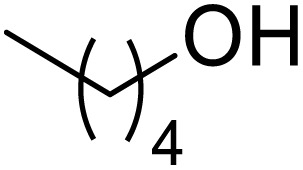	100%
3	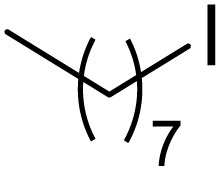	2.2	1	CH_2_Cl_2_	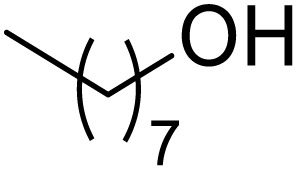	95%
4	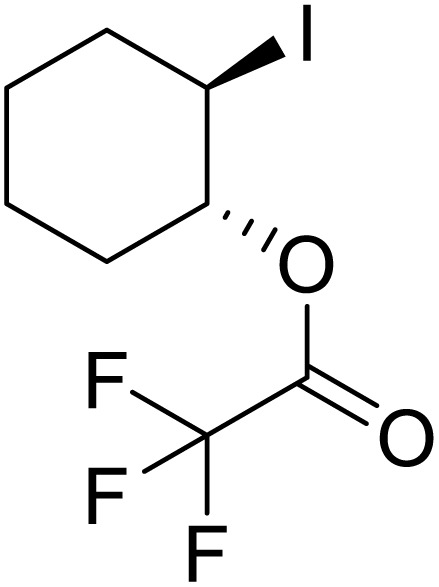	2.2	180	CH_2_Cl_2_	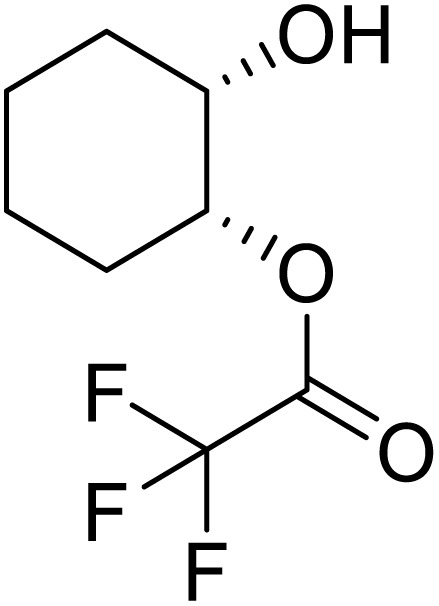	100%
5	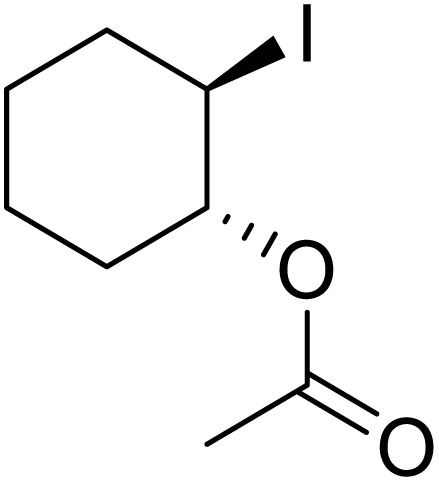	2.2	60	CH_2_Cl_2_	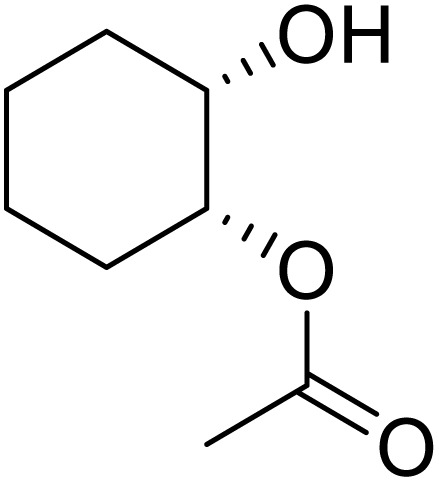	95%
		3	15	*t*-BuOH/water		78%
6	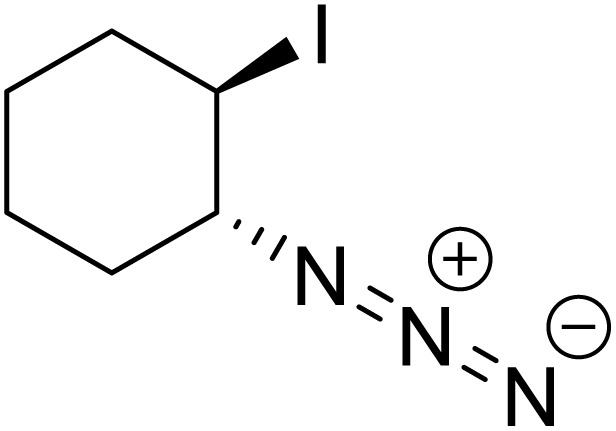	3	5	CH_2_Cl_2_	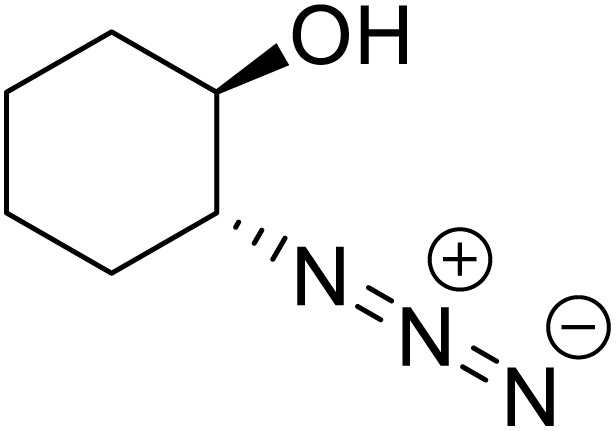	77%
		4	10	*t*-BuOH/water		84%
7	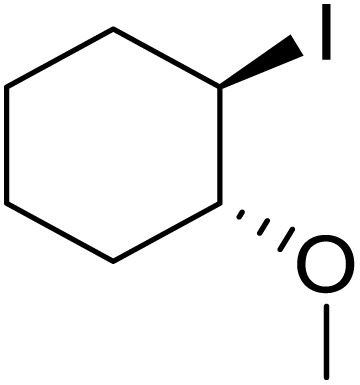	2.2	5	CH_2_Cl_2_	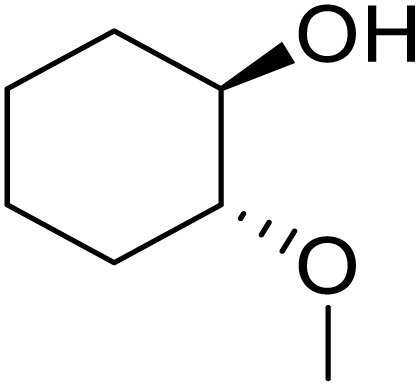	31%
		3	30	*t*-BuOH/water		67%
8	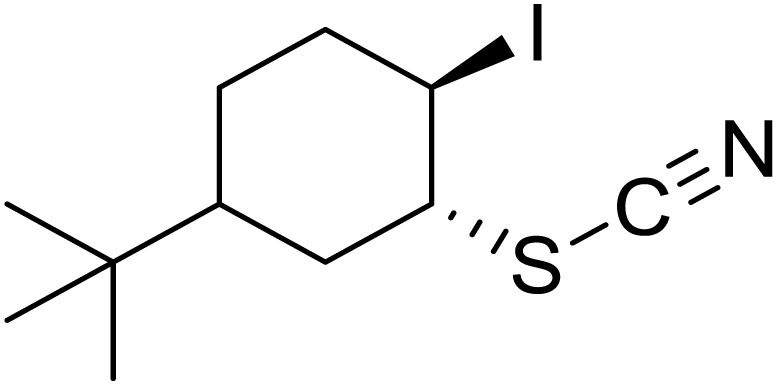	4.5	5	CH_2_Cl_2_	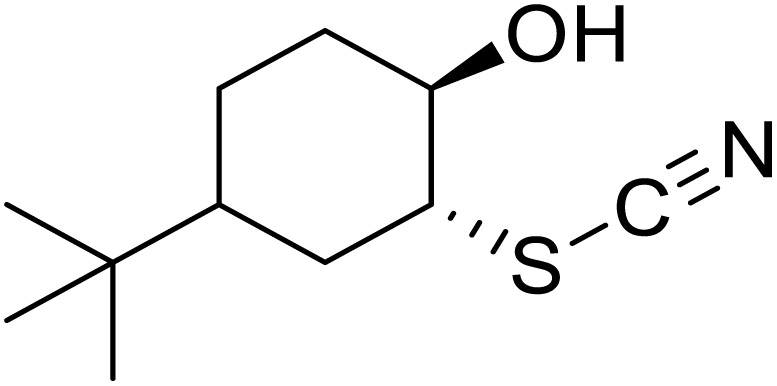	34%
		10	60	*t*-BuOH/water		77%
9	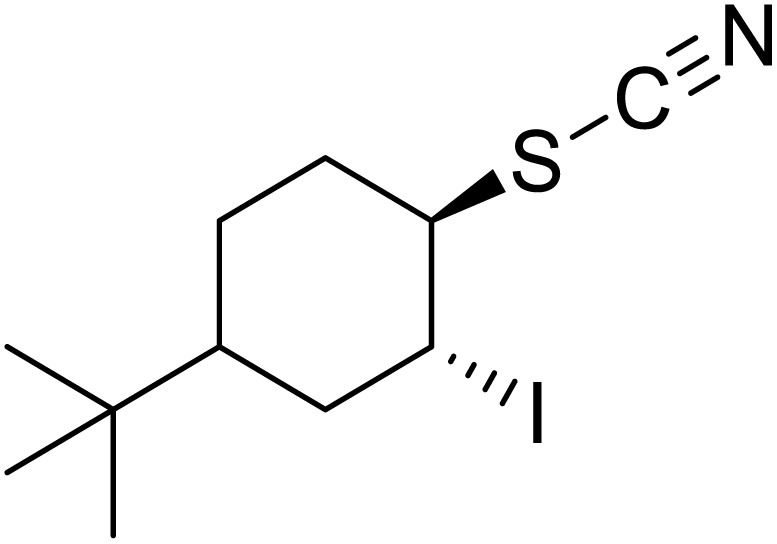	4.5	5	CH_2_Cl_2_	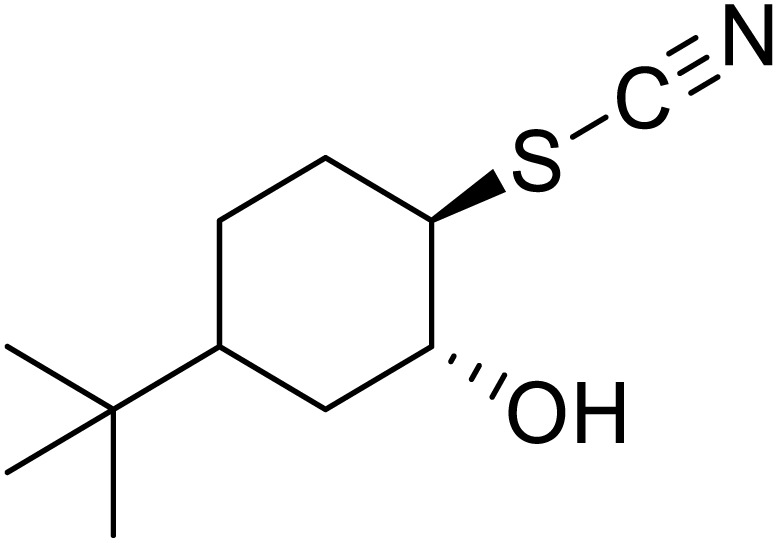	34%
		10	60	*t*-BuOH/water		77%
10	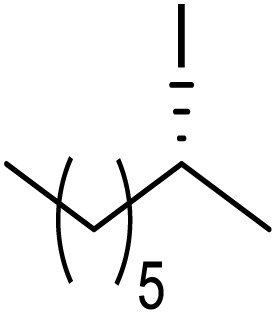	2.2	10	CH_2_Cl_2_	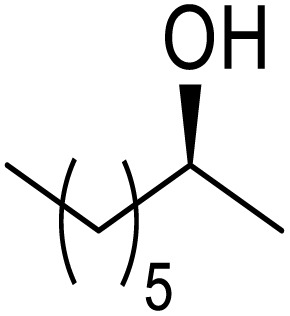	56%
		2.2	10	*t*-BuOH/water		53%
11	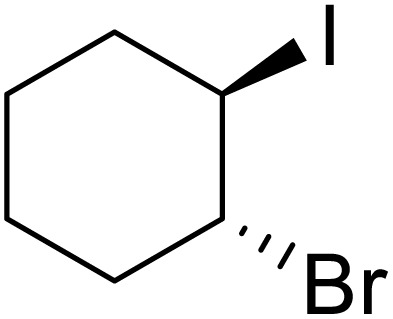	3	7	*t*-BuOH/water	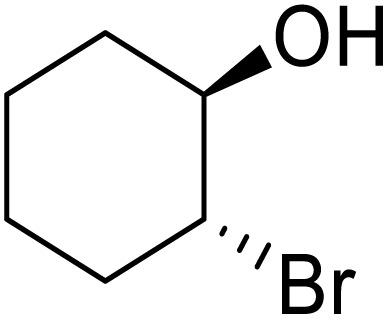	74%
12	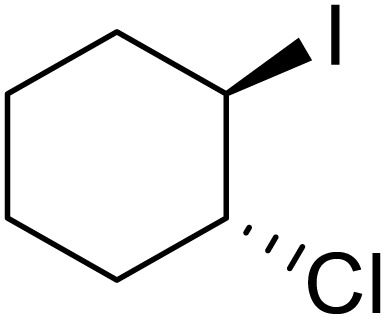	4	60	*t*-BuOH/water	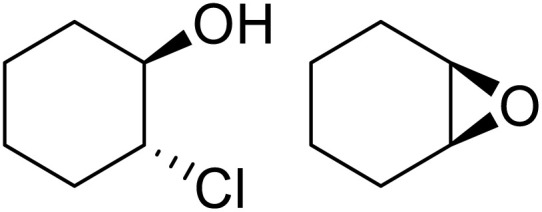	4 : 1 ROH/epoxide

An in-depth study of the chemical and biological oxidation of alkyl halides was undertaken by Macdonald and colleagues (Table 9).^64^ Oxidative displacement of primary alkyl iodides in the presence of *m*CPBA proceeded smoothly to give the expected alcohols (entries 1–3). The yields for secondary alkyl iodides were markedly reduced with significant amounts of ketone and epoxide side products recovered (entries 4–5). By contrast, neither ketone nor epoxide formation was observed in the case of *tert*-butyl iodide, although some of the benzoate ester was generated (entry 6). Although the authors found the effect of solvent or temperature was much less important than substrate structure, the addition of water was accompanied by a reduction in side products (entry 2 *vs.* 7).

**Table 9:** *m*CPBA-mediated displacement of iodides from alkyl iodides

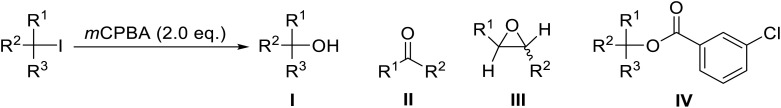										
Entry	R^1^	R^2^	R^3^	Solvent	Temp. (°C)	Time (min)	Ratio I	Ratio II	Ratio III	Ratio IV
1	(H_3_C)_3_C	H	H	CH_2_Cl_2_	27	8	85%	0%	0%	5%
2	*n*-C_6_H_13_	H	H	CH_2_Cl_2_	27	6	90%	0%	0%	10%
3	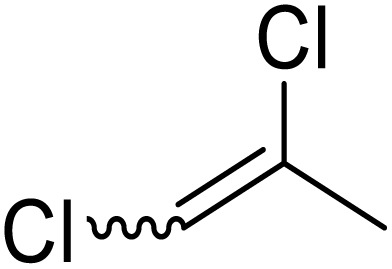	H	H	CH_2_Cl_2_	27	5	100%	0%	0%	0%
4	*n*-C_6_H_13_	CH_3_	H	CH_2_Cl_2_	40	3	31%	29%	24%	16%
5	–(CH_2_)_5_–		H	CH_2_Cl_2_	27	8	54%	14%	32%	0%
6	CH_3_	CH_3_	CH_3_	CH_2_Cl_2_	27	2	85%	0%	0%	15%
7	*n*-C_6_H_13_	H	H	CH_2_Cl_2_/Water	27	8	100%	0%	0%	0%

**Scheme 6:**
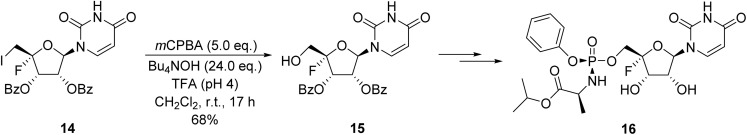
Hydroxylation of antiviral precursors using *m*CPBA.

This approach has been successfully applied to the synthesis of antiviral prodrugs for the treatment of hepatitis C (Scheme 6).^65^ The oxygen atom bound to C5 of the ribose sugar in 16 was installed as a hydroxyl group following treatment of 14 with five equivalents of *m*CPBA in a buffered dichloromethane solution. Alcohol 15 was obtained in a 68% yield after 17 hours at room temperature.

### 2.4 Oxygen-mediated reactions

The use of oxygen as an oxidant has many obvious advantages given that it is inexpensive, sustainable and environmentally benign.^66^ A common feature of this chemistry is the reliance on transition metal catalysts,^67^ although methods have also been developed using biocatalysis.^68^ Many of the methods described herein require the addition of tin hydrides for initial reductive cleavage of the C–X bond which is then oxygenated to the target alcohol.^69–71^ This approach, however, is mostly limited to alkyl iodides, although conversion of alkyl bromides has been achieved in some cases. The application of ultrasonic irradiation may be beneficial, expanding the reaction scope to include tertiary alkyl iodides, as well as affording higher overall yields.^71^ However, organotin/tin hydride additives are problematic due to their toxicity.^72^ Interestingly, a combination of superoxide/crown ether does permit the transformation of alkyl chlorides and bromides to their respective alcohols, albeit in poor to moderate yields, something which is typically outside the scope of molecular oxygen-based radical chemistry.^73^

Tin hydrides are widely employed in the reductive cleavage of carbon–halogen bonds.^74^ When conducted in the presence of molecular oxygen, carbon–halogen bonds may be converted to carbon–oxygen bonds. Nakamura and co-workers successfully employed this strategy for the conversion of alkyl halides to alcohols (Table 10).^69^ The transformation of allylic halides proceeded smoothly with the regiochemistry of the starting material mostly retained (entries 1–3). Ultrasound irradiation proved beneficial for allylic substrates (entries 2–3). It is critical that the reactions are conducted at low temperatures as oxygenation of the *Z*-isomer (entry 2) at 95 °C resulted in a 61% yield of the *E*-allylic alcohol. Unactivated primary (entry 4) and tertiary (entry 5) alkyl iodides were also amenable to this approach. For chiral substrates, the stereoselectivity was found to be low (entries 6 and 7). Other functional groups were well tolerated including silyl ether (entry 8) and Boc (entry 9) protecting groups.

**Table 10:** Aerobic conversion of halides to alcohols in the presence of tributyltin hydride

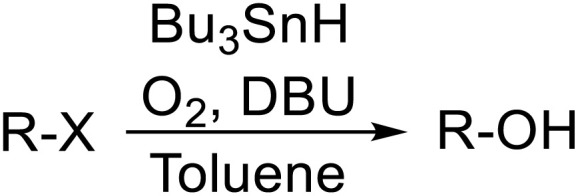						
Entry	R	X	Bu_3_SnH (eq.)	Time (h)	Temp. (°C)	Yield
1^a^	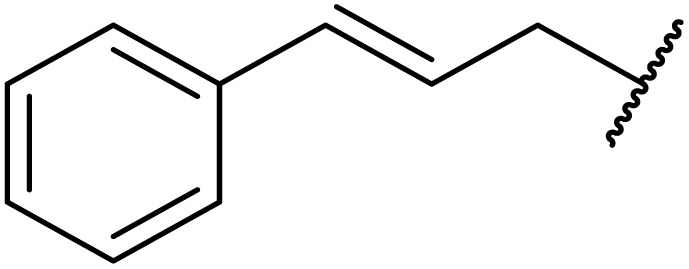	Br	2.1	24	0–20	80%
2^a^^,^^b^^,^^c^	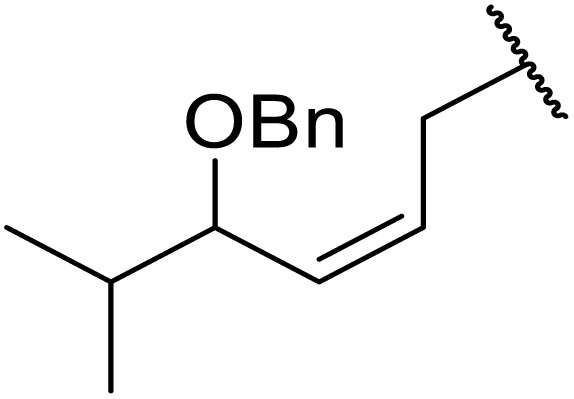	Br	2.1	16	0–7	71%
3^b^	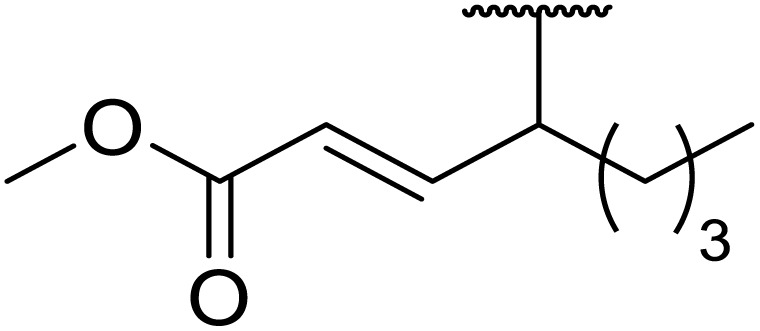	Br	2.1	24	0–30	55%
4^d^	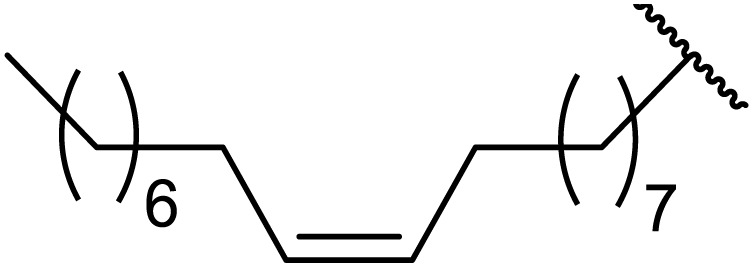	I	2.5	20	−20–0	70%
5^d^	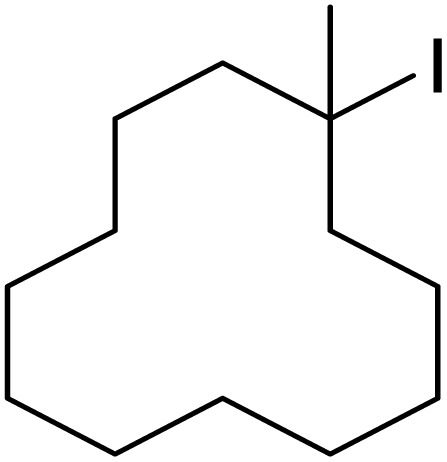	I	2.5	4	0–20	89%
6^b^	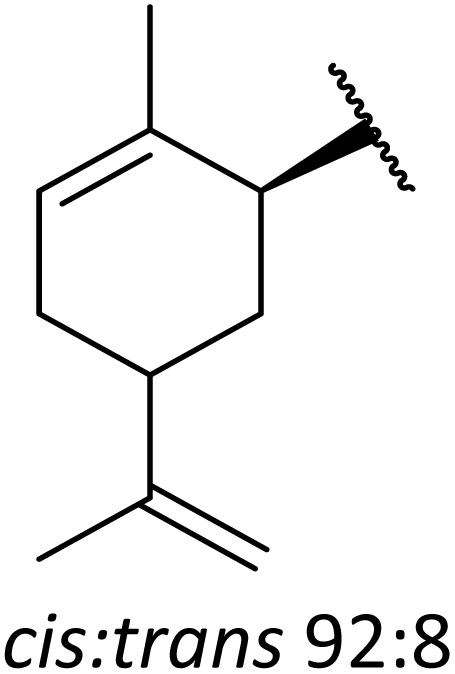	Br	2.1	10	12	81% *cis* : *trans* 52 : 48
7	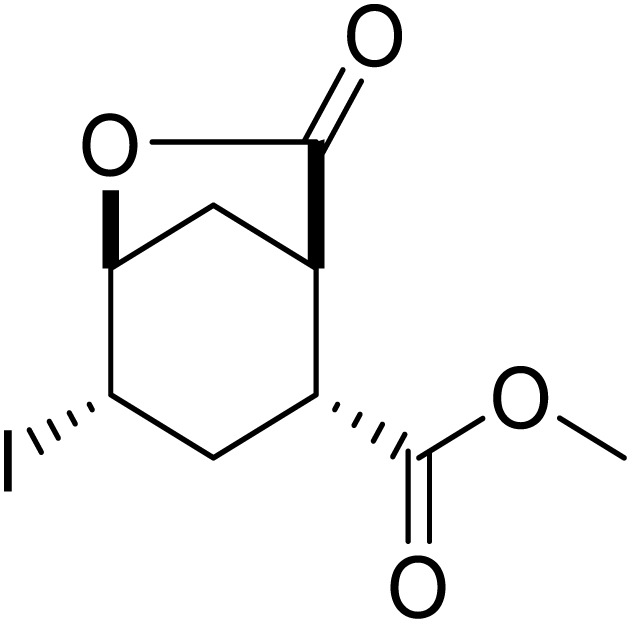	I	3.0	22	0–20	58% *cis* : *trans* 52 : 48
8	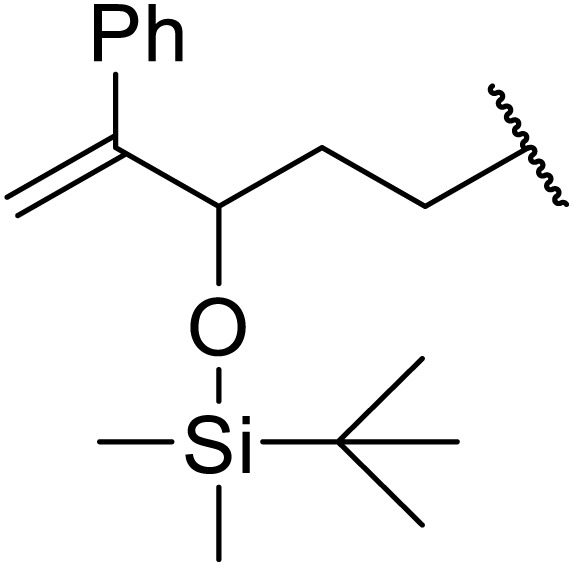	I	3.0	26	0–20	91%
9^d^	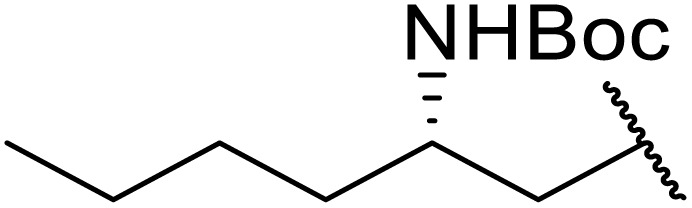	I	2.1	6	0–20	65%

a 0.3 eq. NaBH_4_ added at end of reaction.

b Under ultrasound radiation.

c MeCN as solvent.

d 0.1 eq. PPh_3_ added at end of reaction.

Nakamura further developed this work by way of an organotin chloride/sodium cyanoborohydride catalyst system which provided access to ^18^O- and ^17^O-labelled alcohols.^70^ The combination of 1 mol% azobisisobutyronitrile (AIBN) and either Bu_3_SnCl or Bu_2_(*t*-Bu)SnCl (5 mol%) in the presence of two equivalents of sodium cyanoborohydride with continuous bubbling of oxygen was an effective system for transforming a variety of alkyl halides (Table 11). Good to high yields of the target alcohols were obtained from primary (entries 1–8), secondary (entries 9–10) and tertiary (entries 11–13) alkyl bromides and iodides. Conversion of alkyl bromides to the corresponding alkyl iodides *via* Finkelstein reactions was necessary in some cases (entry 4). Transformation of allylic bromides occurred with complete retention of stereo- and regiochemistry to furnish *trans*-cinnamyl alcohol exclusively (entry 14). Although Bu_3_SnCl or Bu_2_(*t*-Bu)SnCl may be employed as a catalyst, the use of Bu_2_(*t*-Bu)SnCl minimises the formation of unwanted alkane side products (entry 1 *vs.* 3, entry 5 *vs.* 6, entry 11 *vs.* 12). Alternatively, similar results can be achieved by adding small amounts of perfluorodecaline to increase oxygen solubility in the reaction mixture (entry 1 *vs.* 2). This approach also allows for the preparation of radiolabelled alcohols using either ^18^O_2_ or ^17^O_2_ gas (entries 7, 8, 10 and 13).

**Table 11:** Aerobic conversion of halides to alcohols in the presence of a radical initiator

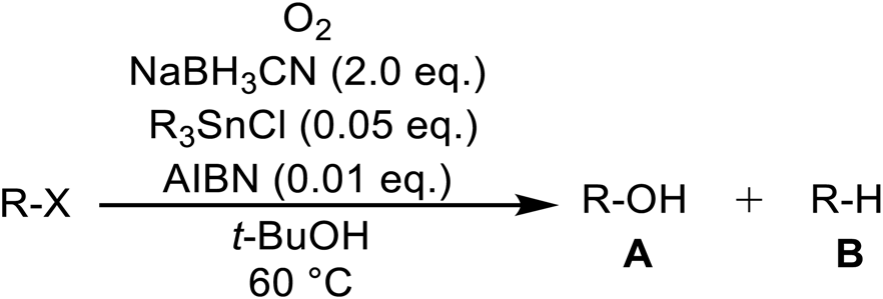							
Entry	R	X	Catalyst	Time (h)	Isotope^c^	Yield of A	Ratio of A : B
1	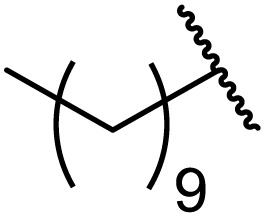	I	Bu_3_SnCl	16	—	68%	70 : 30
2^a^	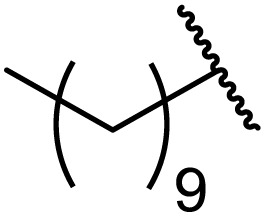	I	Bu_3_SnCl	14	—	93%	93 : 7
3	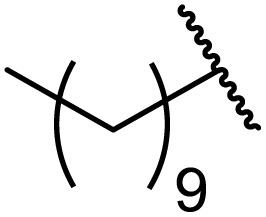	I	Bu_2_(*t*-Bu)SnCl	19	—	82%	91 : 9
4^b^	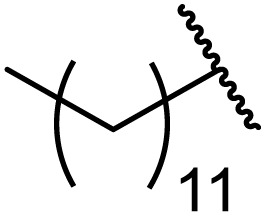	Br	Bu_2_(*t*-Bu)SnCl	12	—	90%	93 : 7
5	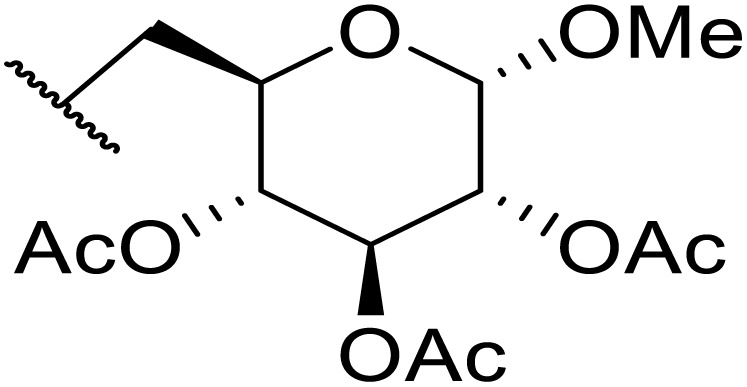	I	Bu_3_SnCl	19	—	77%	84 : 16
6	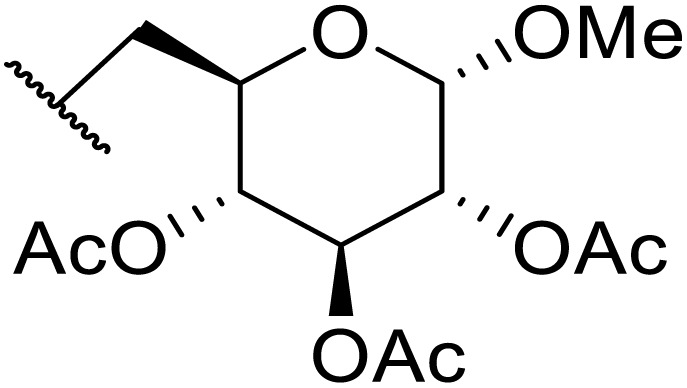	I	Bu_2_(*t*-Bu)SnCl	11	—	88%	91 : 9
7	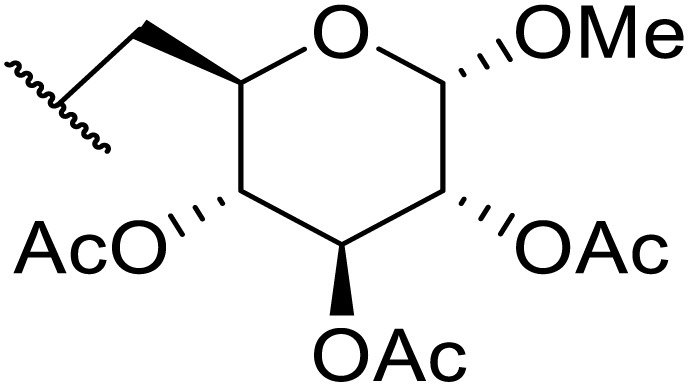	I	Bu_2_(*t*-Bu)SnCl	13	^18^O^c^	88%	—
8	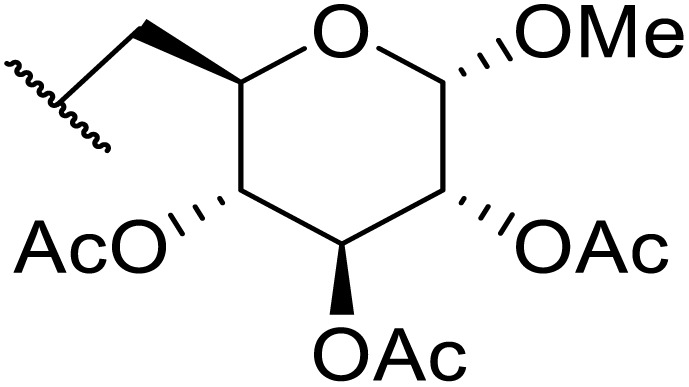	I	Bu_2_(*t*-Bu)SnCl	13	^17^O^c^	80%	—
9	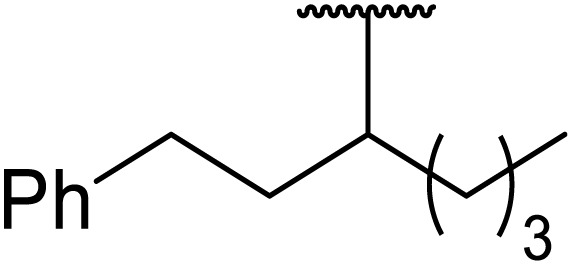	I	Bu_2_(*t*-Bu)SnCl	10	—	92%	93 : 7
10	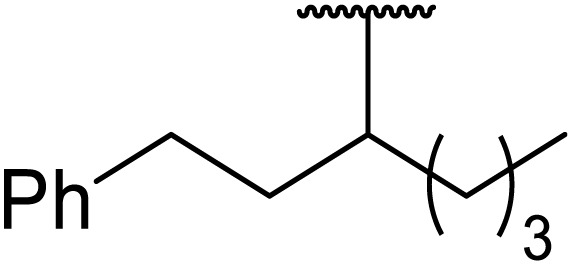	I	Bu_2_(*t*-Bu)SnCl	15	^18^O^c^	98%	—
11	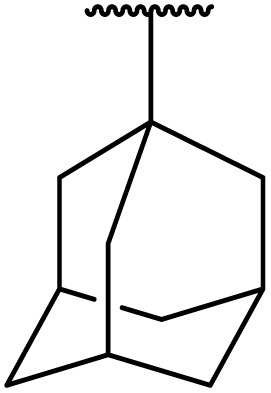	I	Bu_3_SnCl	18	—	94%	96 : 4
12	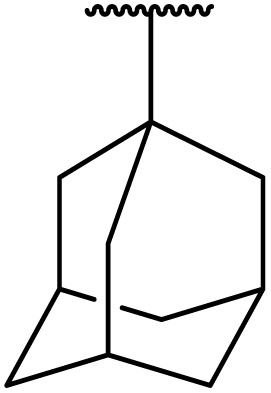	I	Bu_2_(*t*-Bu)SnCl	20	—	96%	97 : 3
13	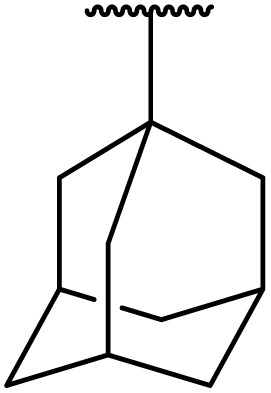	I	Bu_2_(*t*-Bu)SnCl	16	^18^O^c^	98%	—
14	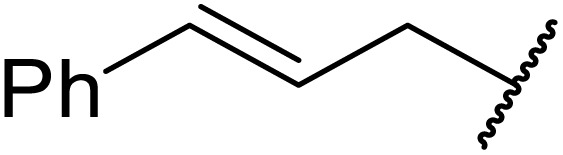	Br	Bu_2_(*t*-Bu)SnCl	18	—	69%	70 : 30

a 3 eq. perfluorodecaline added.

b 2 eq. NaI added.

c 99 atom % ^18^O_2_ (2 eq.) or 55 atom % ^17^O_2_ (1.5 eq.) was used.

A further improvement in this chemistry was reported by the same authors employing ultrasound irradiation, with primary, secondary, tertiary, benzylic and allylic alkyl halides being converted in almost quantitative yields (Table 12).^71^ All of the test substrates were converted to the expected alcohols in excellent yields (entries 1–6) apart from cinnamyl bromide which was characterised by increased side product formation (entry 7). Transformation of acyclic tertiary alkyl iodides was accompanied by small amounts of olefin side product (entry 4).

**Table 12:** Aerobic conversion of halides to alcohols under ultrasonic radiation

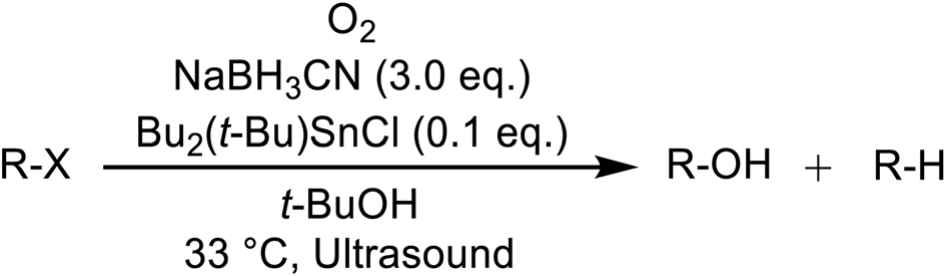					
Entry	R	X	Time (h)	Yield of ROH	Yield of RH
1	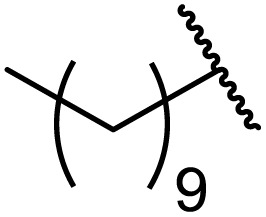	I	20	98%	2%
2	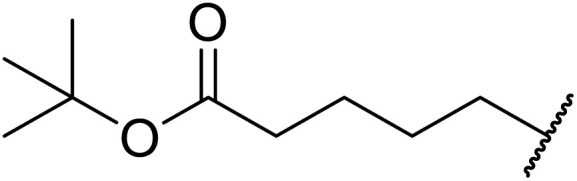	I	16	95%	5%
3	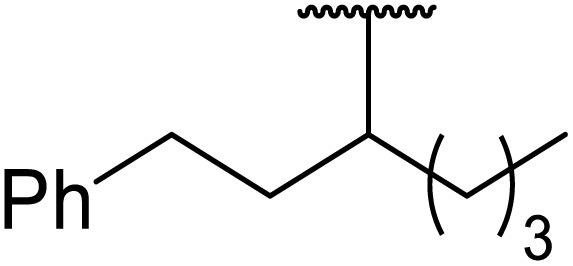	I	13	100%	0%
4	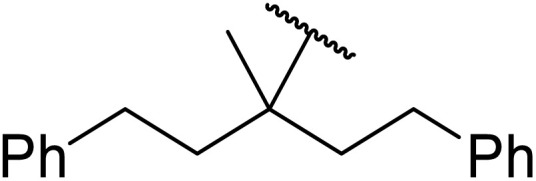	I	22	90%	0%
5	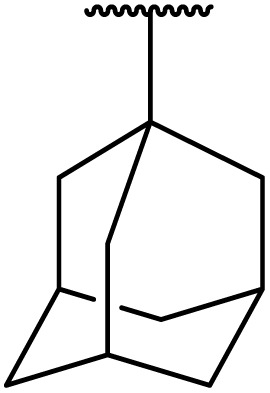	I	18	100%	0%
6	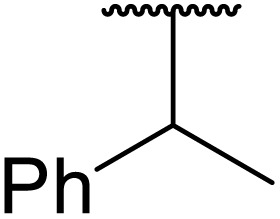	Br	20	96%	3%
7	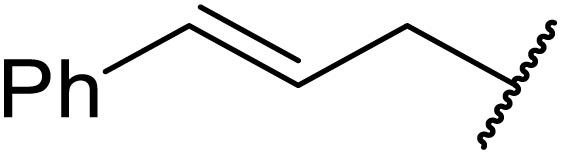	Br	16	60%	25%

**Scheme 7:**
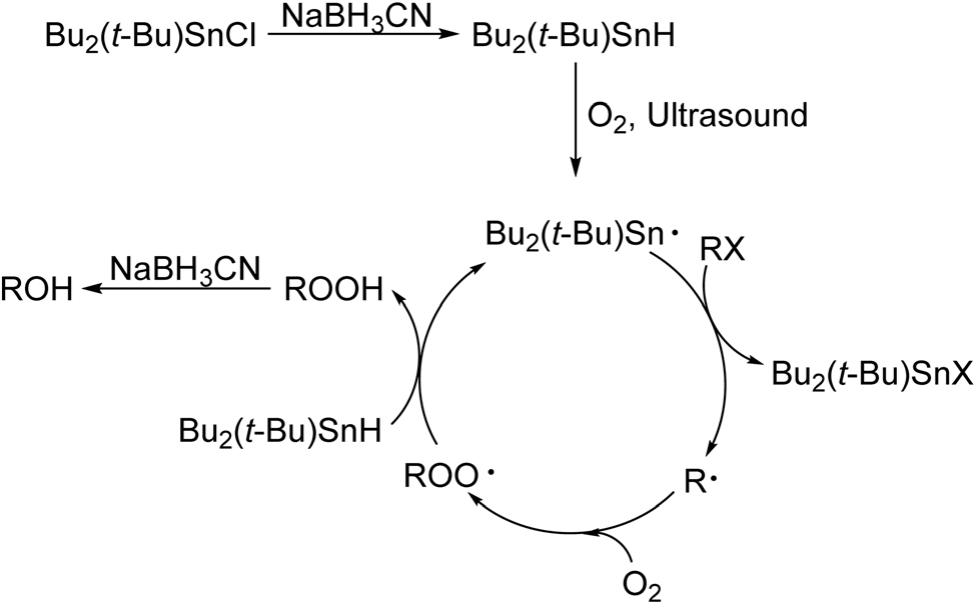
Catalytic cycle.

This mechanism proceeds *via* generation of a radical intermediate which is then oxygenated to form a peroxide and, finally, reduced to the alcohol (Scheme 7). No reaction was observed in the absence of ultrasound at 33 °C. Although the reaction did proceed above 50 °C, significant quantities of unwanted side products were also generated. The authors postulate that ultrasound irradiation facilitates tin radical formation, as well as the generation of Bu_2_(*t*-Bu)SnH from Bu_2_(*t*-Bu)SnCl and NaBH_3_CN.

A one-pot, radical oxygenation of α-iodocarboxylate derivatives was reported by Renaud and colleagues.^73^ Typically, a solution of the α-iodocarbonyl in dichloromethane under an oxygen atmosphere was treated with triethylborane as radical initiator over 5 hours, followed by reduction with dimethyl sulfide. This methodology was compatible with tertiary iodides (Table 13, entries 4, 6) as well as nucleophile-sensitive substrates (entries 3, 7). By contrast, stereochemical control was limited in the case of Evans' oxazolidinone (entry 7) or Oppolzer's sultam (entry 8).^75^ The authors attribute this outcome to the low steric bulk and high reactivity of oxygen.

**Table 13:** Radical oxygenation of α-iodocarboxylic acid derivatives

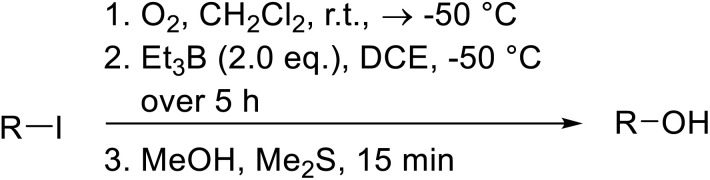		
Entry	R	Yield
1	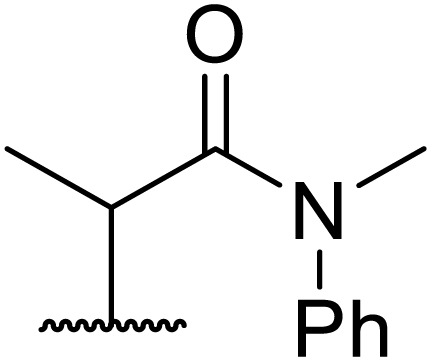	70%
2	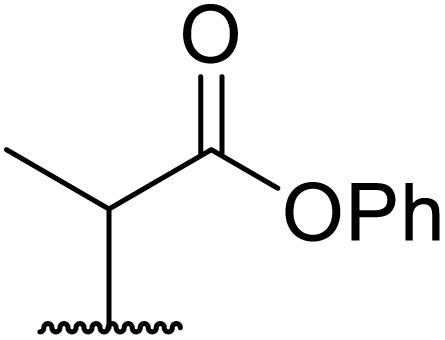	82%
3	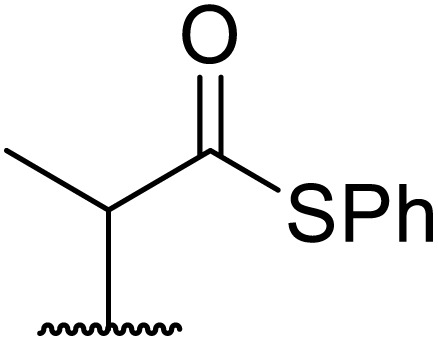	69%
4	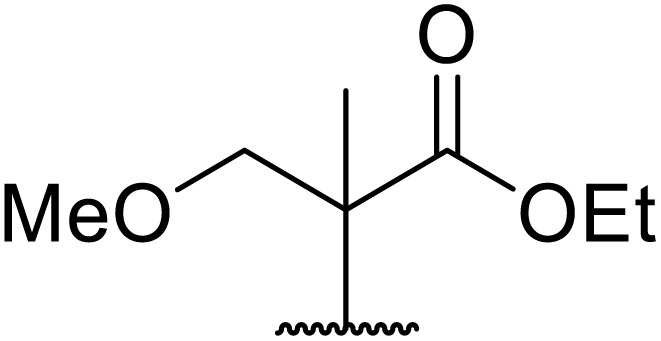	81%
5	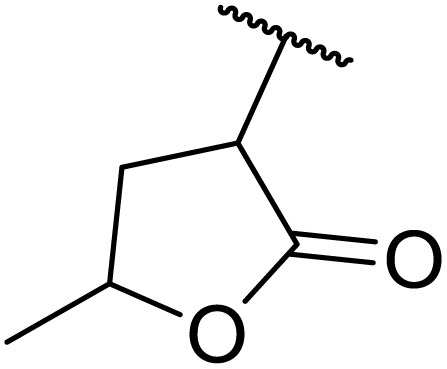	88%
6	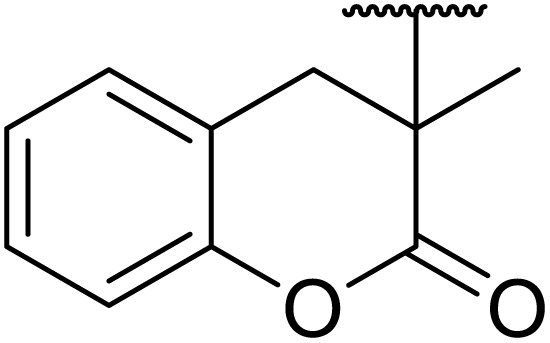	82%
7	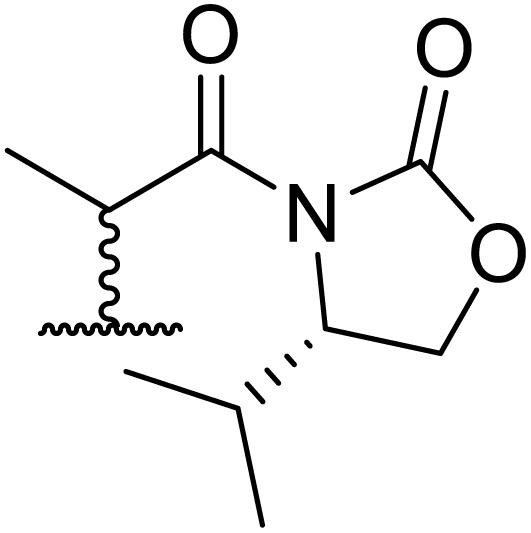	a88%
8	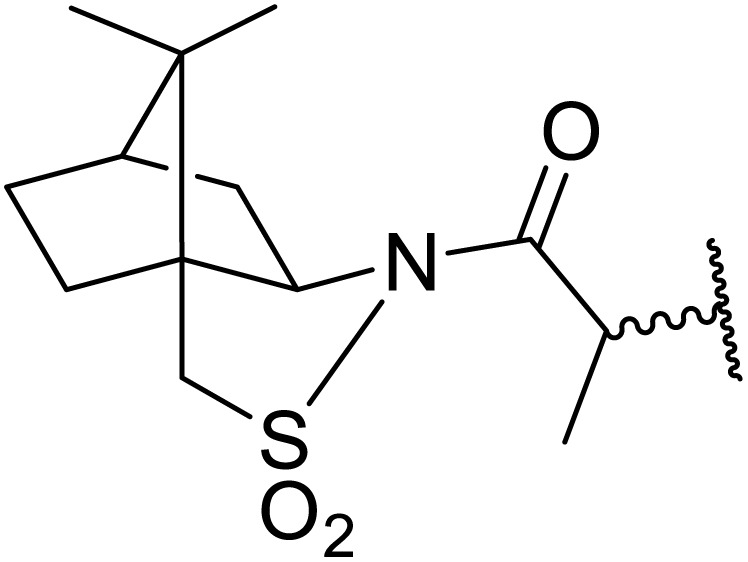	b98%

a dr 60 : 40.

b dr 60 : 40.

San Filippo and co-workers discovered that the reaction of alkyl halides and tosylates with excess potassium superoxide facilitated their rapid conversion to alcohols (Table 14).^76^ The highest yields were obtained using a 3 : 1 ratio of superoxide to alkyl halide in the presence of 18-crown-6. Yields were comparable for primary (entries 1–4), secondary (entries 5–8) and benzylic (entry 10) alkyl halides but were noticeably poorer for tertiary alkyl halides (entry 9). The authors noted that while substitution predominates with primary alkyl halides, an increasing amount of side products was recovered from secondary and tertiary substrates. The substitution of secondary alkyl tosylates and bromides proceeded mostly with inversion (entries 6 and 7) which suggests that a radical type mechanism is not involved.

**Table 14:** Conversion of alkyl halides to alcohols by reaction with superoxide

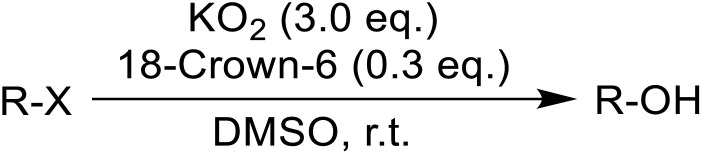					
Entry	R–X	X	Time (h)	R–OH	Yield
1	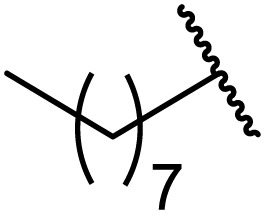	I	1.25	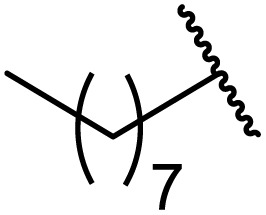	46%
2	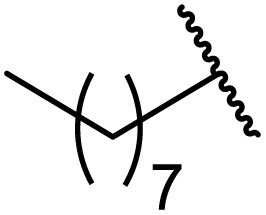	Br	1.25	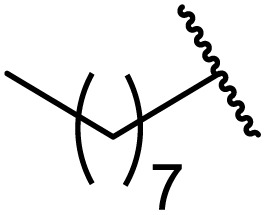	63%
3	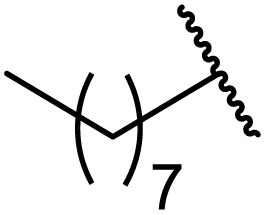	OTs	1.25	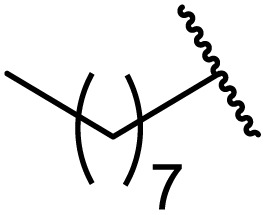	75%
4	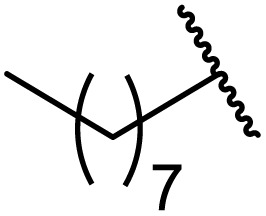	Cl	3	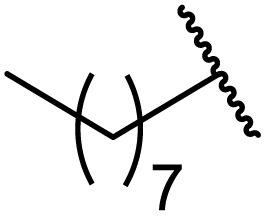	34%
5	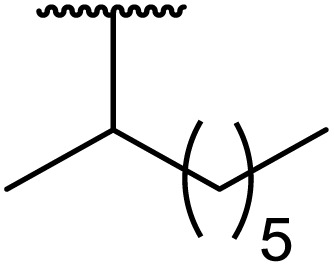	I	1.25	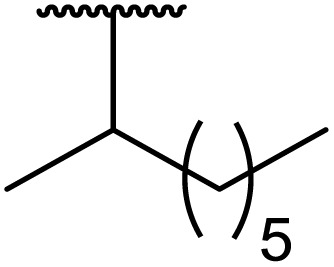	48%
6	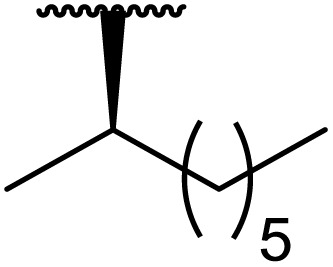	Br	1.25	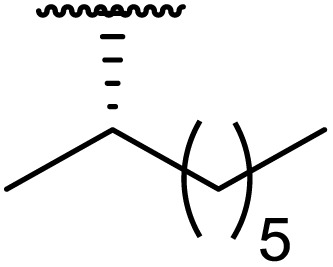	51%
7	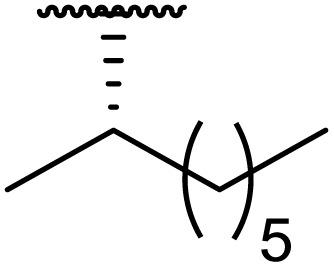	OTs	1.25	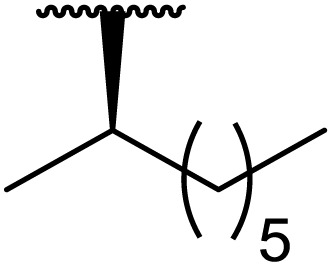	75%
8	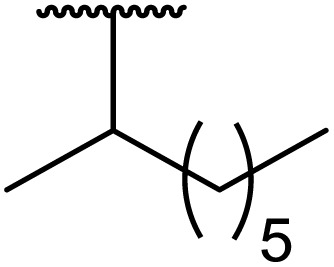	Cl	3	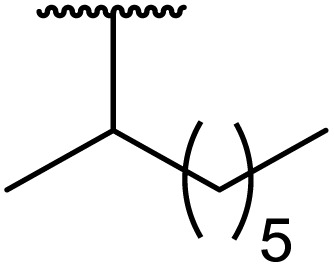	36%
9	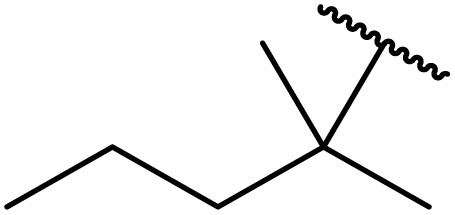	Br	1.25	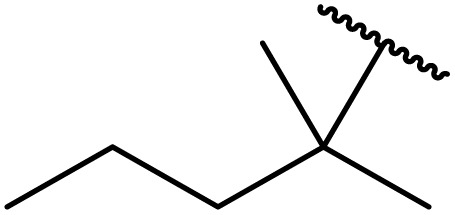	20%
10	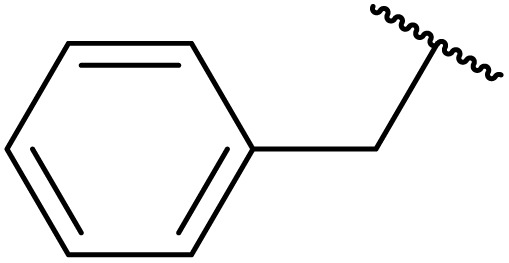	Cl	1.25	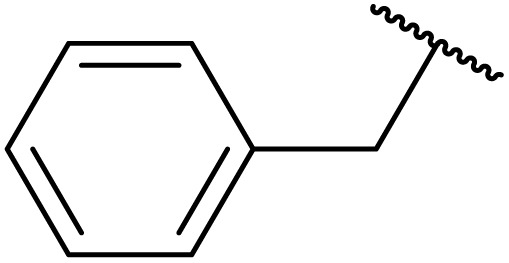	41%

The application of superoxides in this manner has been reported for several natural product syntheses, including prostaglandins,^77^ salinomycin^78^ and boscartin F.^79^

### 2.5 Ionic liquid catalysis

Ionic liquids (ILs) are compounds composed of anions and cations with melting points below 100 °C.^80^ The hydrophilicity of ILs can be fine-tuned with the right combination of anions/cations, allowing for the development of so-called “designer solvents”. ILs have several desirable properties including low flammability, low vapour pressure, low volatility, and the ability to dissolve many inorganic and organic compounds.^81^ In addition, ILs have been shown to possess high catalytic potential and good thermal stability.^82^ The sheer scope of reactions that ILs can catalyse is noteworthy, from the first published report of IL catalysis in a Friedel–Crafts acylation,^83^ to their application to the Pechmann reaction,^84^ Koch carbonylation^85^ and Mannich reaction.^86^ Ionic liquids can also increase the nucleophilicity of water and have demonstrated their utility in nucleophilic substitution reactions.^87^ Despite these apparent advantages, there are relatively few examples in the literature of ILs having been applied to the conversion of alkyl halides to alcohols.^88,89^ This is surprising, given that it has been shown that alkyl chlorides/bromides with diverse functionalities can be converted into alcohols in excellent yields in relatively short reaction times.^89^ A potential drawback of IL-mediated reactions is the requirement for elevated temperatures, often 90–110 °C.

**Fig. 3:**
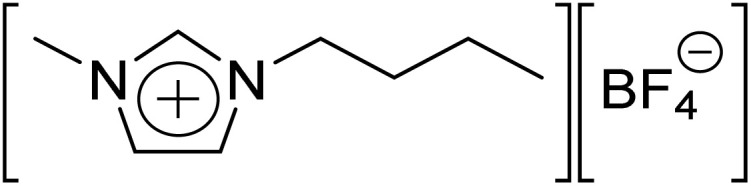
Imidazole/tetrafluoroborate-based ionic liquid.

By way of example, Kim and co-workers demonstrated the nucleophilic hydroxylation of halide-containing compounds.^88^ Using 2-(3-bromopropyl)naphthalane as a model compound, a range of ionic liquids and co-solvents were screened, with 1-*n*-butyl-3-methylimidazolium tetrafluoroborate ([bmim][BF_4_^−^]) in 1,4-dioxane at 110 °C ultimately affording the highest yields (Fig. 3). The addition of 1,4-dioxane was necessary to account for the low solubility of the alkyl bromide in neat [bmim][BF_4_^−^].

Primary aliphatic chlorides returned low yields with mostly starting material recovered (Table 15, entry 1). By contrast, conversion of primary alkyl iodides and bromides proceeded in high yields (entries 2–5). The desired secondary alcohol was similarly obtained in excellent yields from the matching alkyl bromide (entry 6). Significantly, elimination products were not observed during these reactions, while [bmim][BF_4_^−^] could be reused over five cycles without loss of activity.

**Table 15:** Hydroxylation of alkyl halides using [bmim][BF_4_^−^]

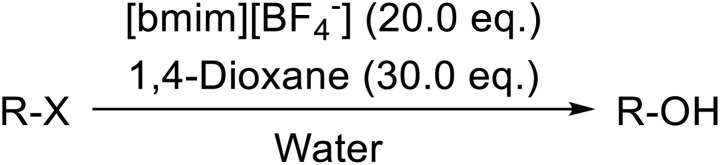					
Entry	R	X	Temp. (°C)	Time (h)	Yield
1	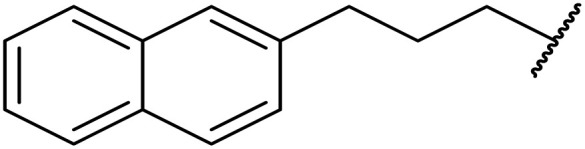	Cl	110	48	5%
2	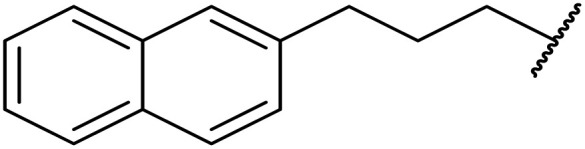	I	100	18	95%
3	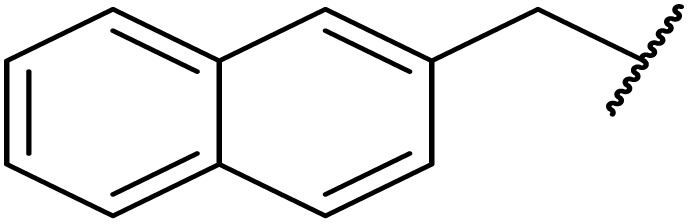	Br	90	3	91%
4	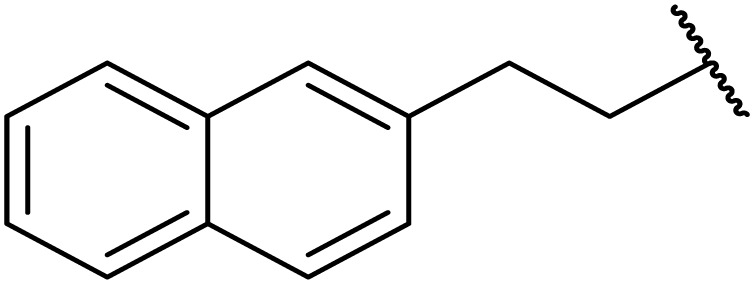	Br	110	72	80%
5	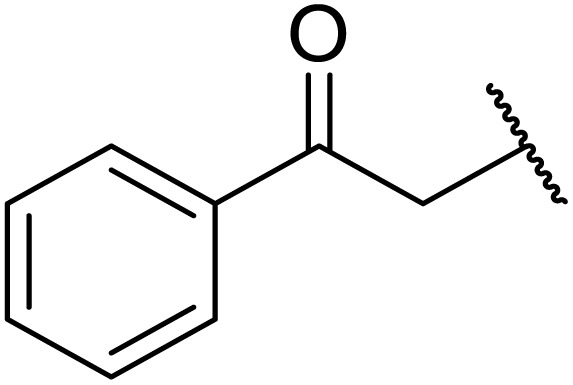	Br	100	12	68%
6	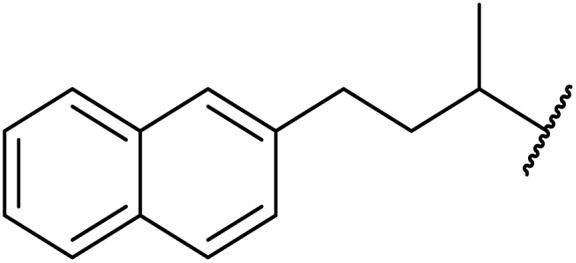	Br	110	48	95%

**Fig. 4:**
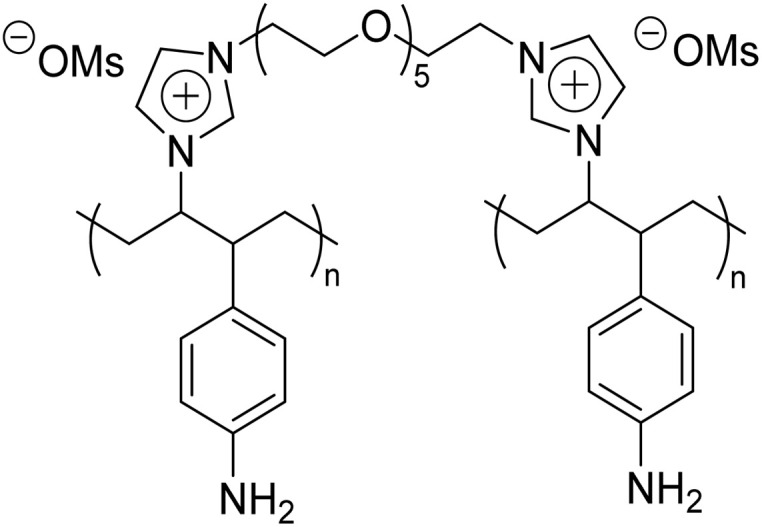
Structure of APS-HEGBIL.

The same authors investigated an amino-polystyrene supported hexaethylene glycol-bridged ionic liquid (APS-HEGBIL) which was initially prepared *via* a cross linking polymerisation between amino styrene and hexaethylene glycol-bridged dicationic 1-vinyl imidazole salt (Fig. 4).^89^

Primary alkyl bromides, chlorides and iodides were converted in excellent yields (Table 16, entries 1–12). The addition of both APS-HEGBIL and potassium carbonate was crucial for successful hydroxylation as their absence resulted in significant alkene formation in a competing elimination process. This chemistry was compatible with secondary alkyl halides (entry 13) and base-sensitive substrates (entries 7, 12 and 13). High yields were also obtained from biologically active (entries 8 and 9) and other complex substrates (entries 10 and 11). The utility of this chemistry was exemplified by the successful hydroxylation of an extremely base-sensitive Fmoc-containing alkyl chloride in excellent yield (entry 12).

**Table 16:** Hydroxylation of various alkyl halides using APS-HEGBIL

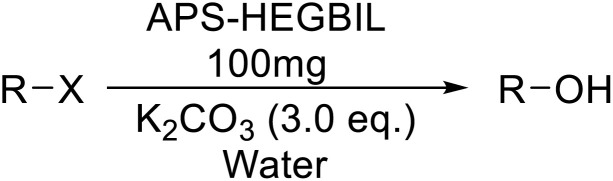					
Entry	R	X	Temp. (°C)	Time (min)	Yield
1	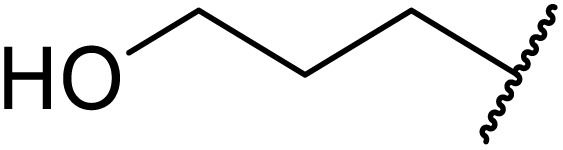	Br	90	30	96%
2	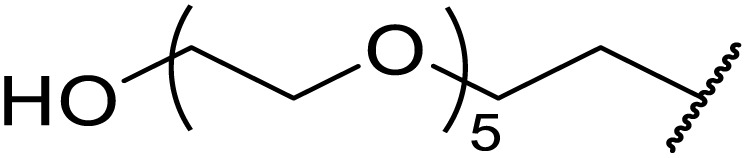	Cl	90	42	98%
3	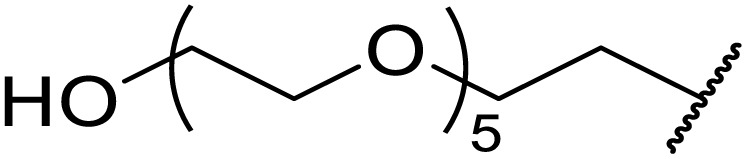	I	90	38	97%
4	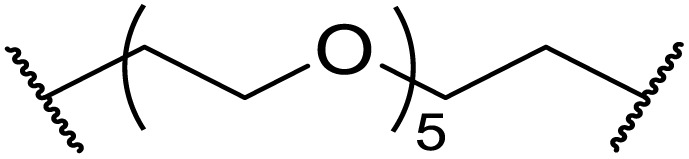	Br	90	30	96%
5	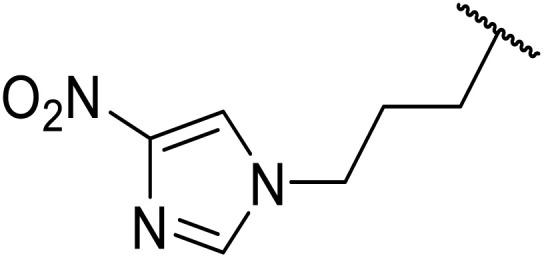	Br	90	40	97%
6	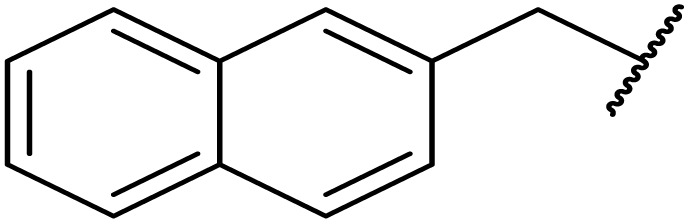	Br	100	35	98%
7	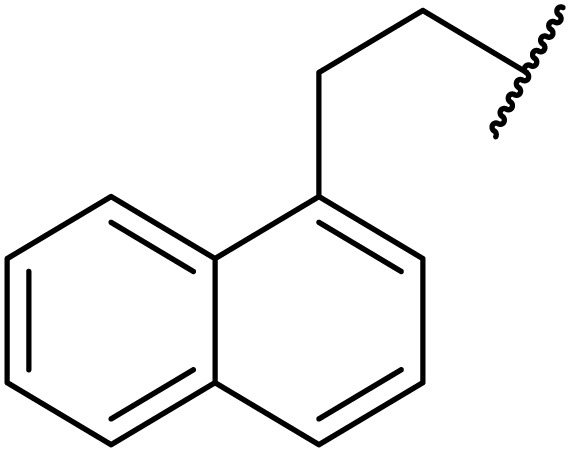	Br	110	250	96%
8	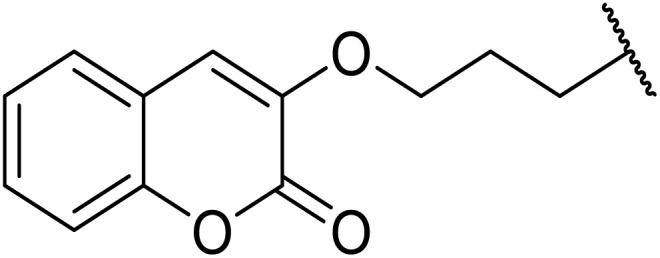	Br	90	95	96%
9	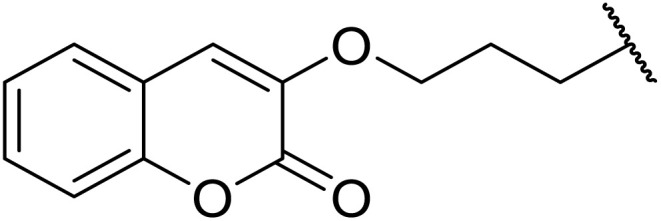	Cl	110	105	95%
10	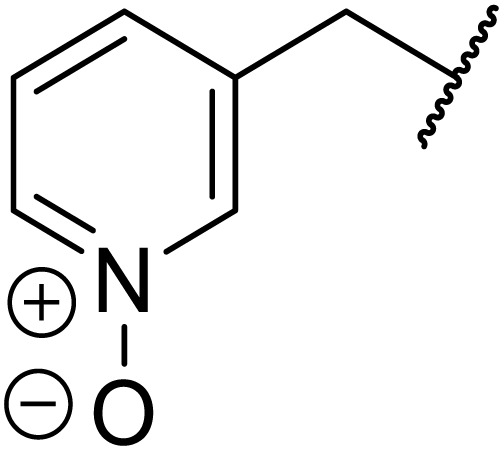	Cl	110	60	96%
11	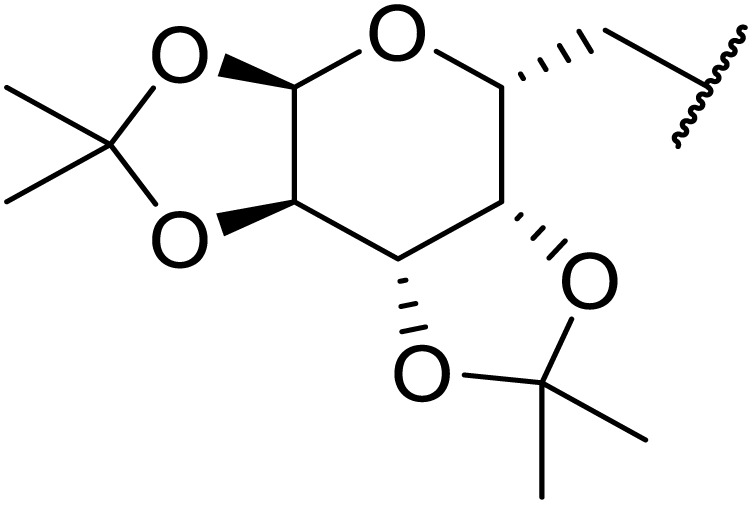	Br	90	180	95%
12	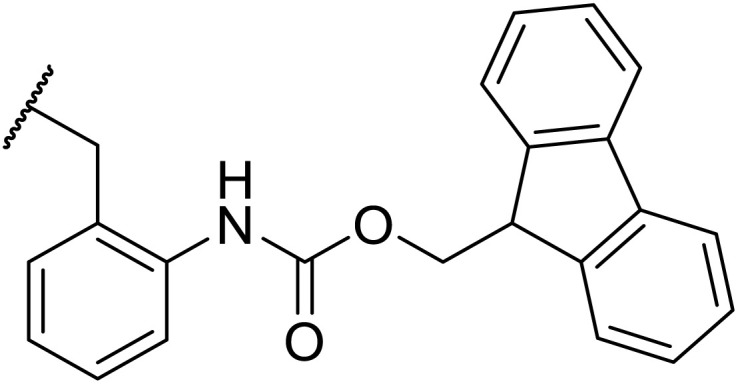	Cl	110	120	92%
13	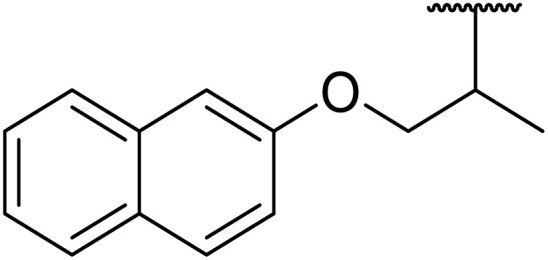	Br	110	200	94%

### 2.6 Mercury-assisted hydroxylations

Despite its high toxicity,^90,91^ mercury and its compounds have found several uses in organic chemistry, such as mercury(ii)-mediated cyclisations of unsaturated compounds,^92^ generation of organometallics *via* metal exchange reactions,^93^ and Markovnikov hydration of alkenes.^94^ For aliphatic halide to alcohol transformations, mercury(ii) oxide is the main mercury salt employed.^95^ Although mercury(ii) oxide can typically convert alkyl bromides to alcohols in good to excellent yields, literature precedent for other halides is limited. Additionally, this mercury salt is incompatible with sensitive functional groups such as nitriles,^96^ although it has been demonstrated that secondary and tertiary bromides are amenable to transformation.^96,97^

McKillop and Ford successfully employed mercury(ii) perchlorate for the preparation of alcohols from alkyl halides.^96^ As this salt can readily decompose when heated,^98^ aqueous solutions were prepared by dissolving mercury(ii) oxide in a mixture of 60% perchloric acid and 1,2-dimethoxyethane to avoid direct handling. Using this approach, a variety of primary alkyl bromides were successfully hydrolysed to the corresponding alcohols in mostly high yields (Table 17, entries 1–14). Nitrile groups did not withstand these conditions as 4-bromobutyronitrile yielded γ-butyrolactone (entry 3). The transformation of secondary alkyl bromides proceeded in similarly high yields (entries 15–19). Finally, tertiary alcohols were recovered in good to high yields from their alkyl bromide equivalents (entries 20 and 21). The authors noted that cyclic bromides were not compatible with this chemistry and gave a complex mixture of products.

**Table 17:** Mercury(ii)-mediated conversion of alkyl bromides to alcohols

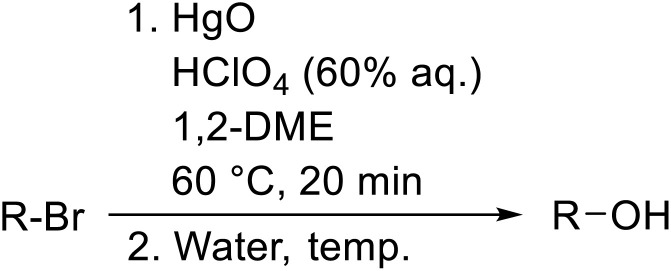				
Entry	R–Br	Temp. (°C)	Time (h)	Yield
1	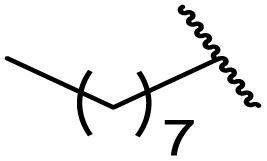	25	3	98%
2	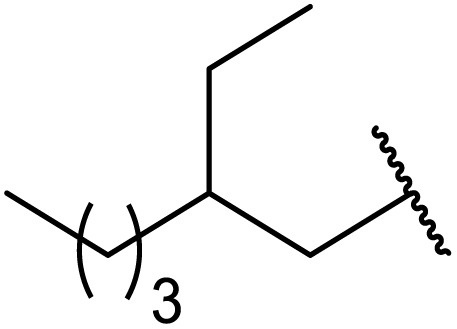	25	1.5	94%
3	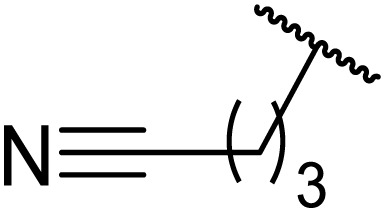	25	3	0%
4	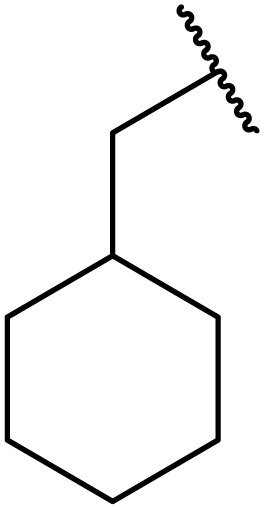	25	1	78%
5	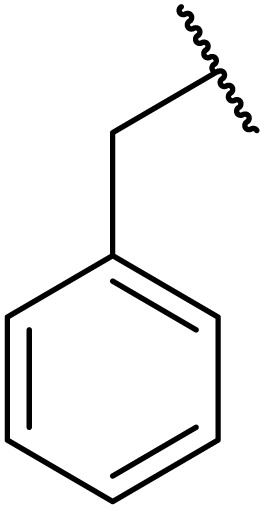	25	0.5	97%
6	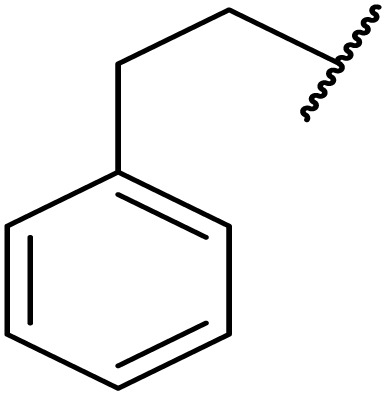	85	0.5	98%
7	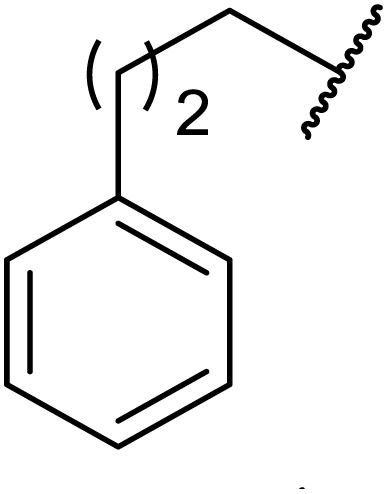	25	4	96%
8	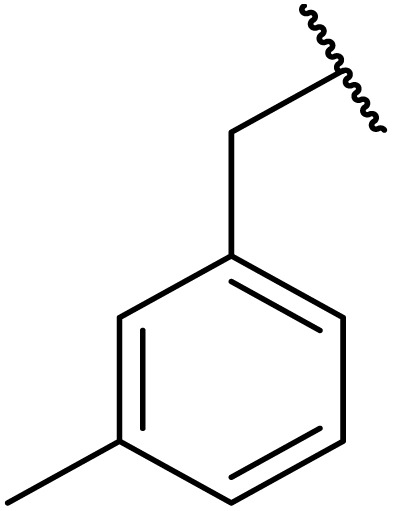	25	0.5	89%
9	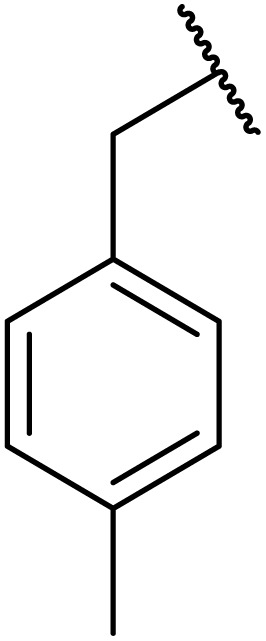	25	0.5	82%
10	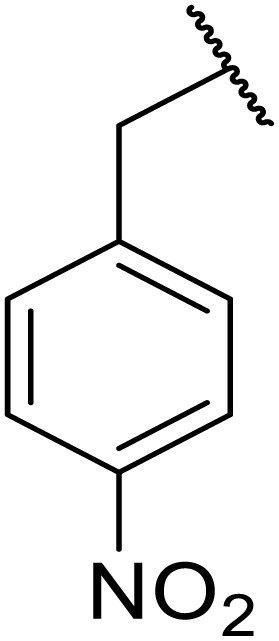	25	0.5	92%
11	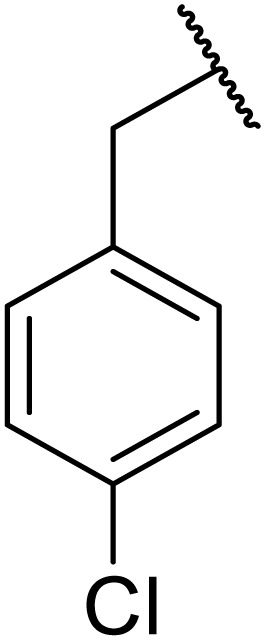	25	0.5	74%
12	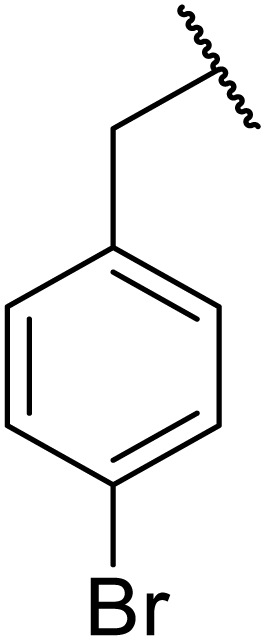	25	0.5	64%
13	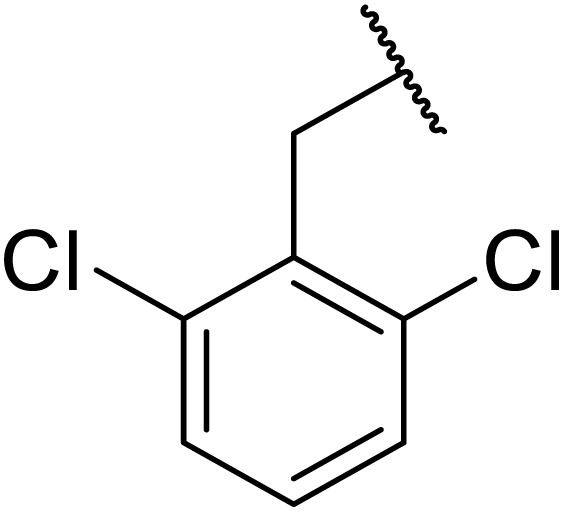	25	0.5	96%
14	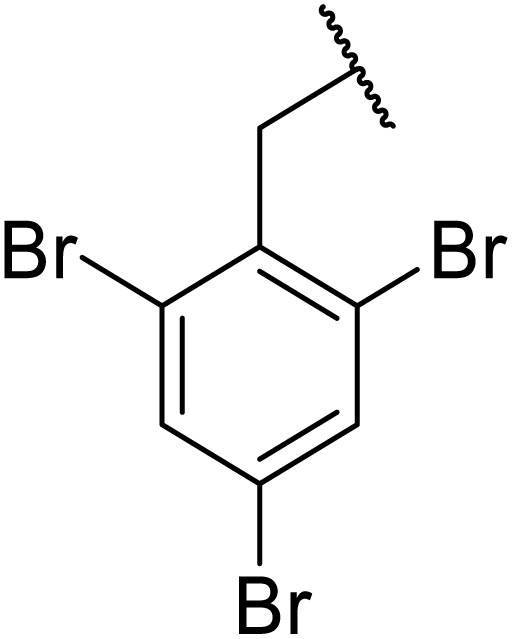	25	0.5	71%
15	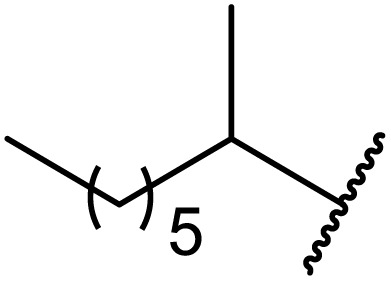	25	1	88%
16	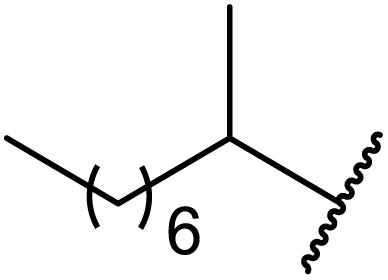	25	1	91%
17	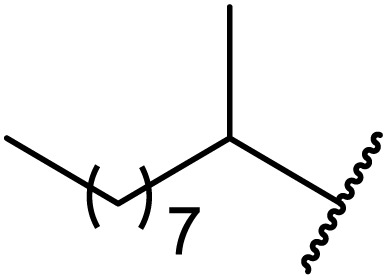	85	1	79%
18	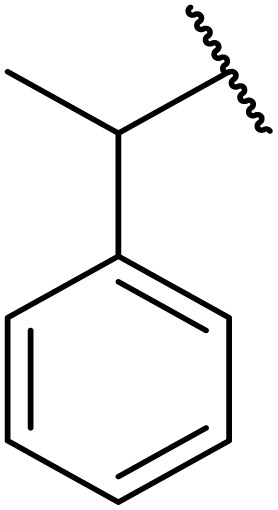	25	0.5	98%
19	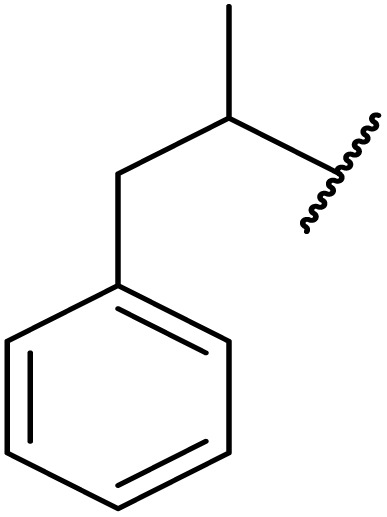	25	1	60%
20	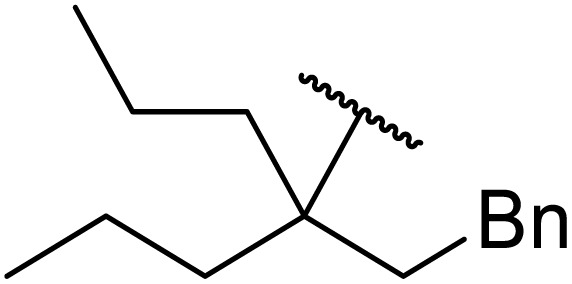	25	1	60%
21	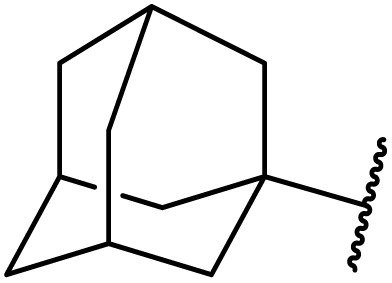	25	3	92%

Barluenga and co-workers developed a unique system composed of mercury(ii) oxide and tetrafluoroboric acid (TFBA) which combines a highly electrophilic mercury(ii) salt with an anion of low nucleophilicity (Table 18).^97^ Good to high yields of primary alcohols were obtained from the corresponding alkyl bromides in either dichloromethane for low molecular weight substrates (entry 1) or dioxane/THF for heavier molecules (entries 2–9). Secondary (entries 6–7) and tertiary (entries 8–9) alkyl bromides were transformed to the desired alcohols in good yields. Mercury(ii) oxide could be quantitatively recovered following alkaline work-up and treatment with 35% aqueous tetrafluoroboric acid. The authors further described how the same catalyst system could be applied to the preparation of ethers from alkyl bromides and alcohols.

**Table 18:** Hydroxylation of alkyl bromides using HgO/TFBA/water

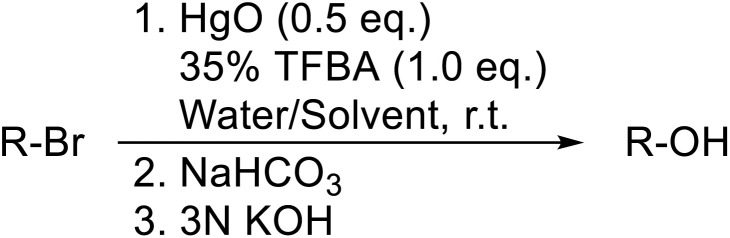				
Entry	R	Solvent	Time (h)	Yield
1	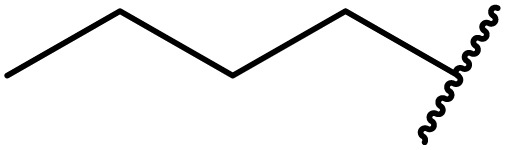	CH_2_Cl_2_	2	67%
2	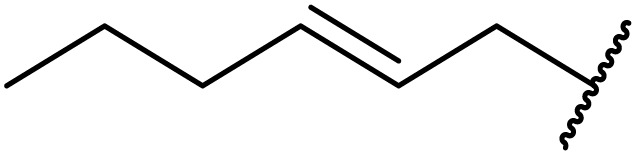	1,4-Dioxane	2	65%
3	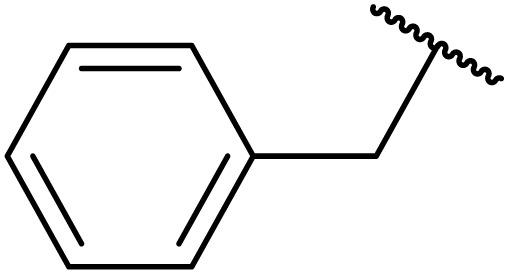	THF	3	79%
4	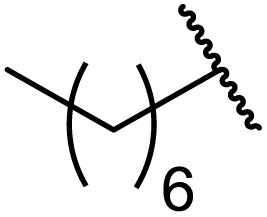	THF	3	93%
5	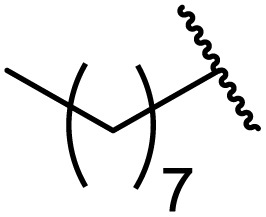	1,4-Dioxane	3	95%
6	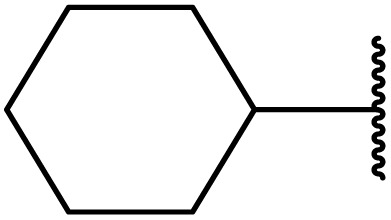	THF	3	72%
7	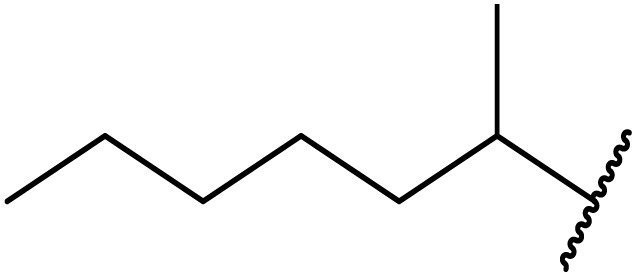	THF	1	77%
8	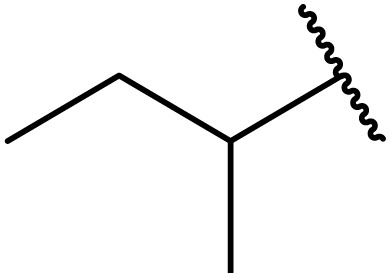	THF	1	69%
9	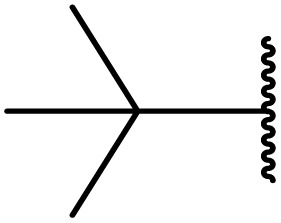	THF	2	81%

### 2.7 Reactions with salts

Salts are often employed to install oxygen-containing functional groups.^35^ Many methods involve harsh conditions which would be unsuitable for substrates with sensitive functional groups. Typically, a hydrolysable ester intermediate is first formed which is then cleaved in a subsequent step. This can be shortened, however, by recourse to anion exchange resins.^99,100^ The most commonly used oxyanion-based salts are those of formic acid. Formate salts possess several distinct advantages, such their compatibility with less reactive halides and labile functional groups. In addition, the hydrolysis step typically requires very mild conditions and often proceeds in excellent yields. Formic acid is corrosive, however, and can cause optic nerve damage.^101^ More recent alternatives include the squarate dianion, which forms a squarate monoester, but this still necessitates hydrolysis of the intermediate under basic conditions.^102^

Harris and Bull developed a mild method for the synthesis of alcohols by adapting an esterification procedure from Larock.^103^ Following initial generation of a formate ester *via* reaction of an alkyl halide with sodium formate in hexamethylphosphoramide, the desired alcohol could be obtained by twice passing the ester through neutral alumina using a dichloromethane/methanol eluent system (Table 19).^104^ Two primary halides were efficiently converted to the target alcohols in high yields using this method (entries 1 and 2).

**Table 19:** Mild conversion of alkyl halides to alcohols

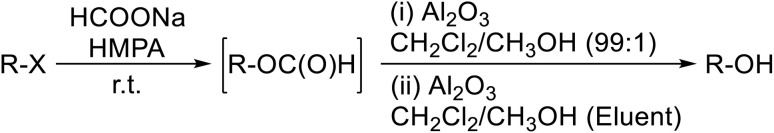					
Entry	R–X	X	Time (h)	Eluent	Yield
1	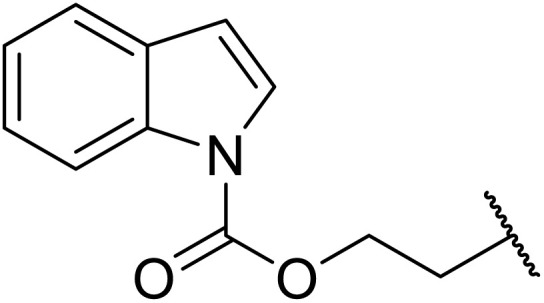	Br	39	95 : 5	96%
2	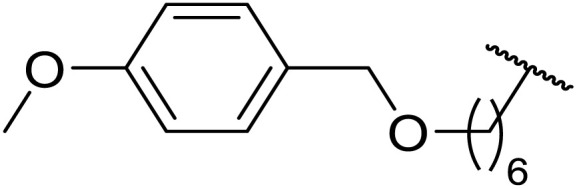	I	27	98 : 2	99%

A one-pot conversion of primary alkyl chlorides/dichlorides to their alcohols/diols was reported by Zahalka and Sasson.^105^ This approach uses phase-transfer catalysis to accelerate the formation of formate esters from alkyl chlorides and their subsequent hydrolysis.^106^ Reactions were conducted under solvent-free conditions in the presence of 5 mol% tetrabutylammonium bromide (TBAB). Conversion of benzylic (Table 20, entry 1), primary alkyl (entry 2), dihalogenated (entries 3–6) and allylic (entry 6) substrates all proceeded in excellent yields. By contrast, the authors noted that alkyl bromides reacted more slowly and afforded lower yields.

**Table 20:** Hydroxylation of primary chlorides/dichlorides under phase transfer catalysis

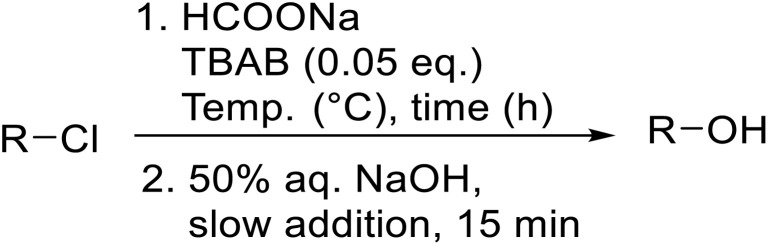						
Entry	R	HCOONa eq.	Temp. (°C)	Time (h)	50% aq. NaOH eq.	Yield
1	Bn	2.0	125	0.5	1.10	94%
2	*n*-Octyl	2.0	125	1.5	1.10	96%
3	–(CH_2_)_3_–	4.0	115	1.5	2.20	94%
4	–(CH_2_)_5_–	4.0	115	1.5	2.20	91%
5	–(CH_2_)_6_–	4.0	115	1.5	2.20	96%
6	–CH_2_CH=CHCH_2_–	4.0	70	3	2.20	97%

Alexander *et al.* adopted a similar approach when they generated a series of formate esters using triethylammonium formate, followed by acid- or base-catalysed hydrolysis (Table 21).^107^ A 3- to 5-fold excess of formic acid and triethylamine was required for non-activated alkyl halides, whereas a 10% molar excess of formic acid to triethylamine was employed for base-sensitive substrates. While reactions were typically conducted solvent-free (entries 1–2 and 4–9), α-bromoketones were rapidly converted to their α-formyl esters in acetonitrile at room temperature (entries 11–13). Yields for the acid- or base-catalysed hydrolysis are not recorded.

**Table 21:** Two-step synthesis of alcohols from alkyl halides using triethylammonium formate

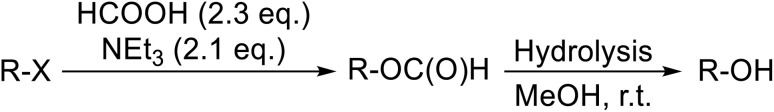						
Entry	R	X	Solvent	Time (h)	Temp. (°C)	Yield of ester
1	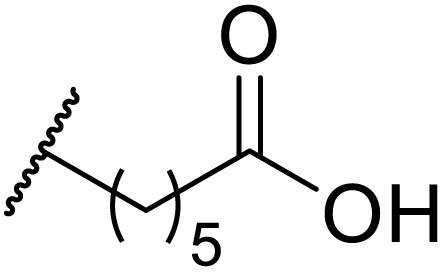	Br	—	18	r.t.	80%
2	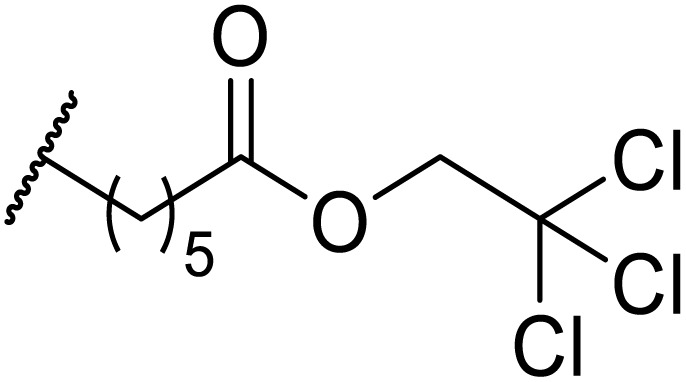	Br	—	16	50	83%
3	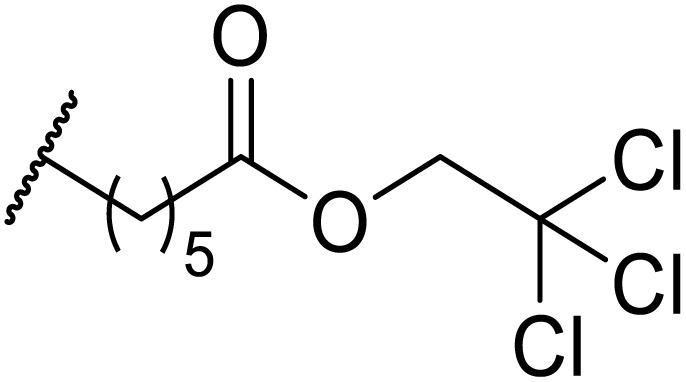	Br	MeCN	8	Reflux	92%
4	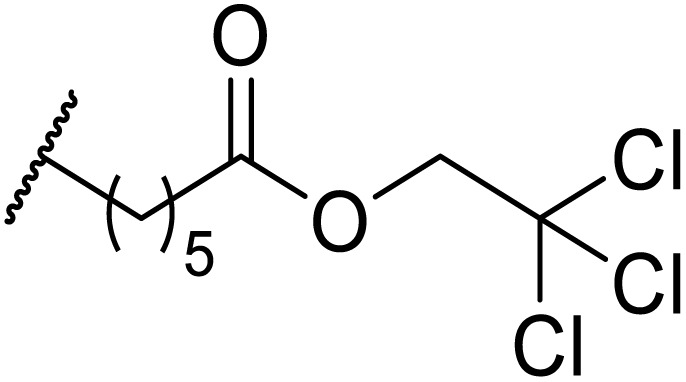	I	—	18	r.t.	76%
5	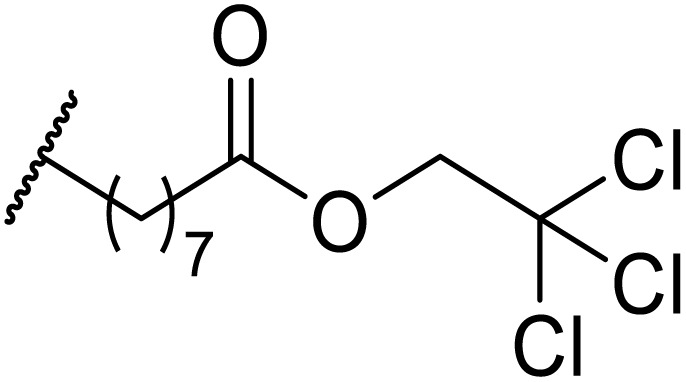	I	—	18	r.t.	79%
6	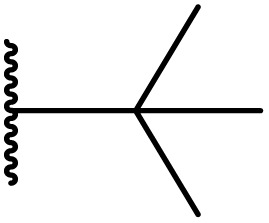	I	—	24	r.t.	0%
7	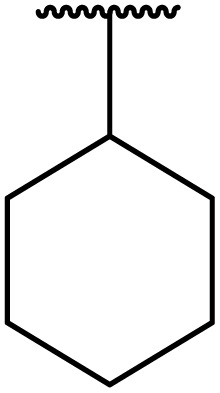	Br	—	18	50	0%
8	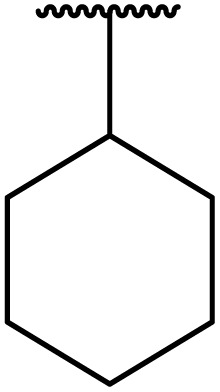	I	—	18	50	0%
9	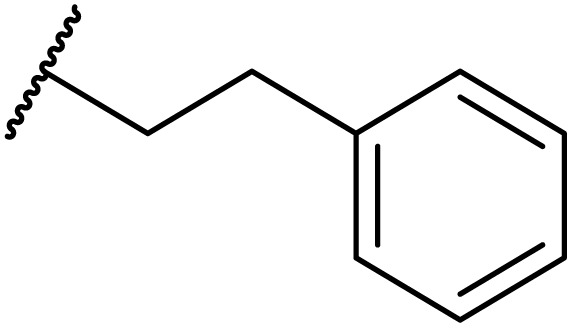	Br	—	12	r.t.	95%
10	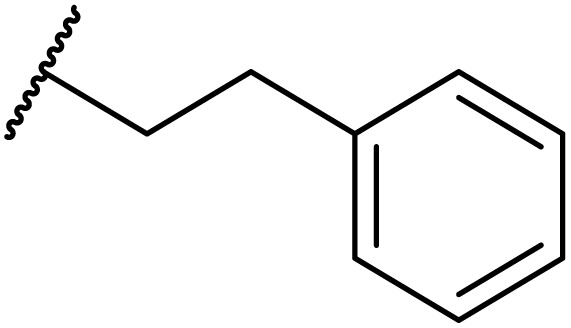	Br	MeCN	24	Reflux	20%
11	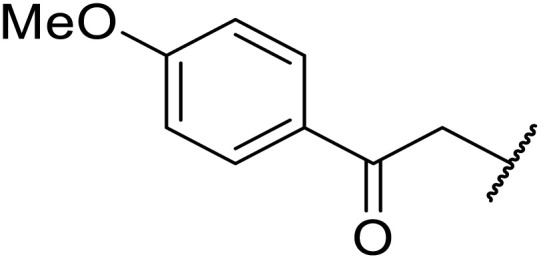	Br	MeCN	2	r.t.	97%
12	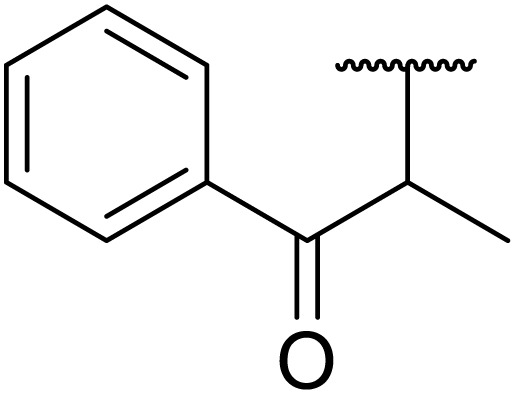	Br	MeCN	2	r.t.	98%
13	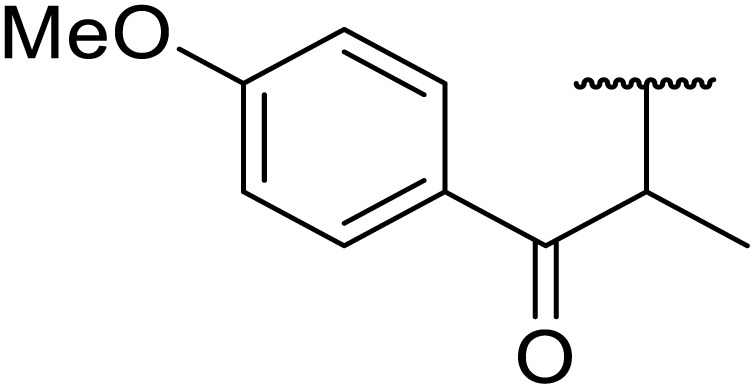	Br	MeCN	1.5	r.t.	96%
14	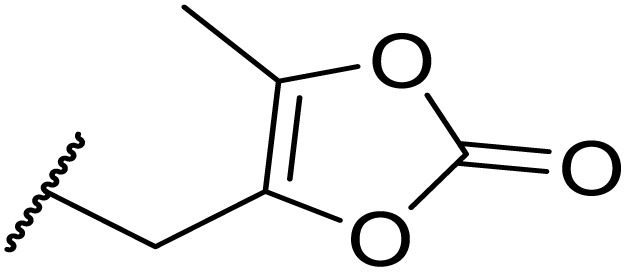	Br	MeCN	1	r.t.	80%
15	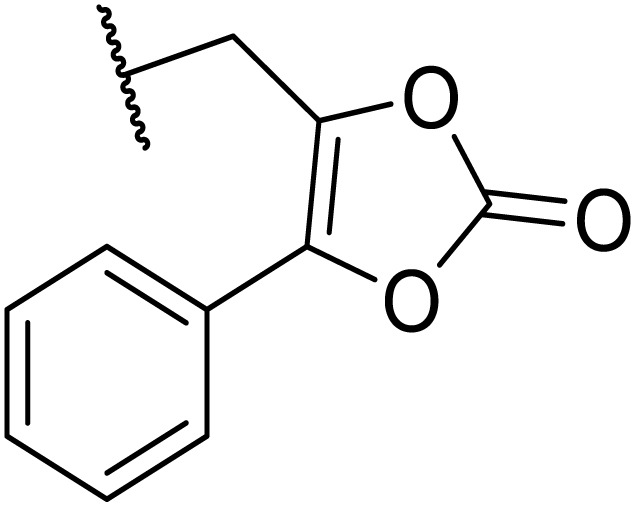	Br	MeCN	1.5	r.t.	92%

A mild methodology for the hydrolysis of alkyl halides using the carbonate form of Amberlyst A-26 anion exchange resin (AER) was developed by Cardillo and co-workers.^99^ Although THF and benzene proved suitable solvents, the higher boiling point of benzene allowed for faster conversions and higher yields. Transformation of chlorides (Table 22, entry 1), bromides (entry 2) and iodides (entry 3) proceeded smoothly, with the former requiring longer reaction times as expected. Elimination products predominated when hydrolysis of secondary alkyl halides was attempted.

**Table 22:** Hydrolysis of alkyl halides using carbonate AER

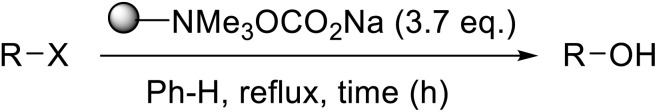				
Entry	R	X	Time (h)	Yield
1	*n*-Octyl	Cl	7	90%
2	*n*-Octyl	Br	4	90%
3	*n*-Octyl	I	4	95%
4^a^	(Me)_2_C=CHCH_2_	Br	1	95%
5	Bn	Cl	2	90%
6	Bn	Br	2	95%
7	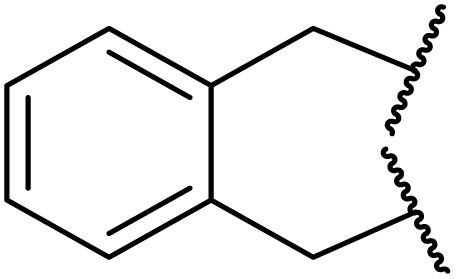	Br	2	85%

a Reaction conducted in THF.

Hodge and colleagues adapted Cardillo *et al.*'s approach in an effort to broaden the substrate scope.^99,100^ The first AER investigated was a formate resin (Table 23, Method A), whereby the halide was converted to the intermediate formate ester. This resin worked well for primary (entries 1–3), cyclopentyl (entry 5), allyl (entry 7) and arylmethyl halides (8–9) but not for secondary (entry 4) or tertiary halides (entry 6). A one-pot procedure was also examined, using a combination of the formate AER with acidic Amberlyst 15 resin (Method B), which gave direct access to the alcohols (entries 10–11). The most effective strategy was Method C, using a bicarbonate AER and an iodide resin as catalyst. The bicarbonate resin is stable at higher temperatures, allowing for higher boiling point solvents and shorter reaction times (entries 12–24). This method was compatible with secondary halides (entry 16) and somewhat successful with alkyl chlorides (entry 12) and tertiary alkyl bromides (entry 18). An important advantage is the ability to conduct reactions without cleaving ester or amide functionalities (entries 22–24).

**Table 23:** Conversion of halides to alcohols with different anion exchange resins

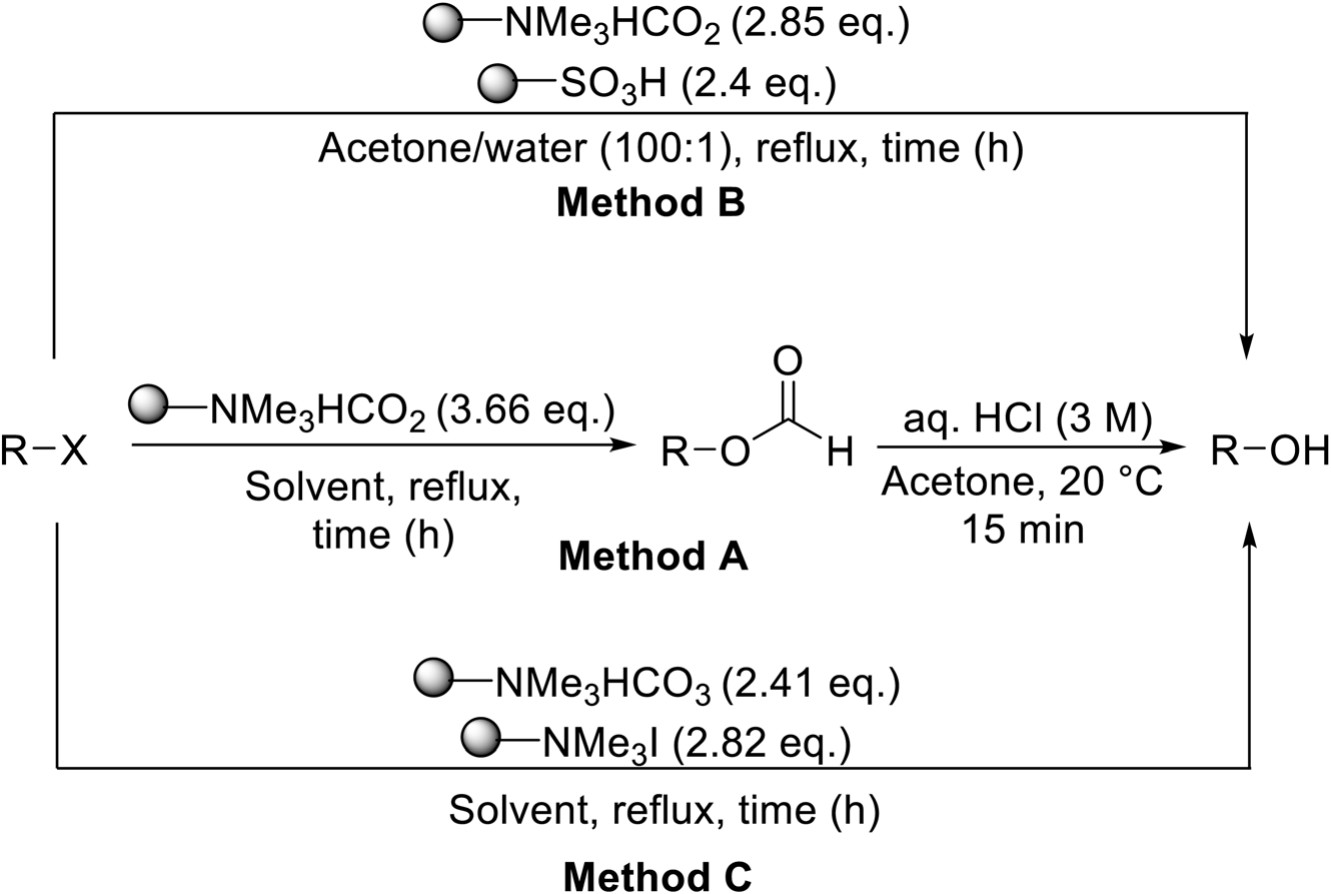							
Entry	R	X	Method	Solvent	Time (h)	Yield (formate)	Yield (alcohol)
1	*n*-Bu	Br	A	Acetone	72	46%	50%
2	*n*-Octyl	Br	A	THF	72	76%	n/a
3	*n*-Dodecyl	Br	A	Acetone	72	100%	100%
4	Me(CH_2_)_6_CHMe	Br	A	THF	72	3%	n/a
5	Cyclopentyl	Br	A	THF	72	72%	n/a
6	*t*-Bu	Cl	A	THF	72	17%	n/a
7	Cinnamyl	Br	A	Acetone	72	78%	88%
8	Bn	Cl	A	Acetone	72	100%	n/a
9	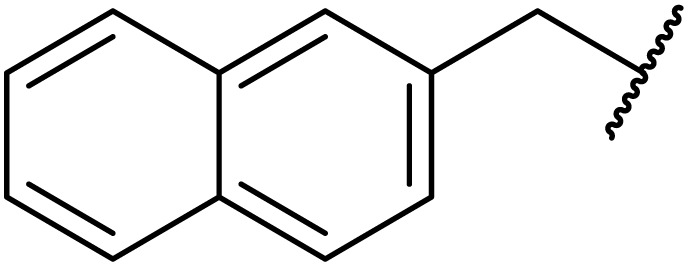	Br	A	Acetone	72	70%	a90%
10	*n*-Octyl	Br	B	n/a	72	n/a	76%
11	Bn	Br	B	n/a	24	n/a	100%
12	*n*-Undecyl	Cl	C	Dioxane	24	n/a	26%
13	*n*-Undecyl	Br	C	Dioxane	24	n/a	68%
14	*n*-Undecyl	I	C	Dioxane	24	n/a	68%
15	Ph(CH_2_)_2_	Br	C	THP	6	n/a	66%
16	Me(CH_2_)_6_CHMe	Br	C	Dioxane	24	n/a	47%
17	Cyclopentyl	Br	C	Dioxane	24	n/a	54%
18	*t-*Bu	Br	C	Acetone	24	n/a	a22%
19	Cinnamyl	Br	C	THP	3	n/a	64%
20	4-BrC_6_H_4_CH_2_	Br	C	THP	2	n/a	71%
21	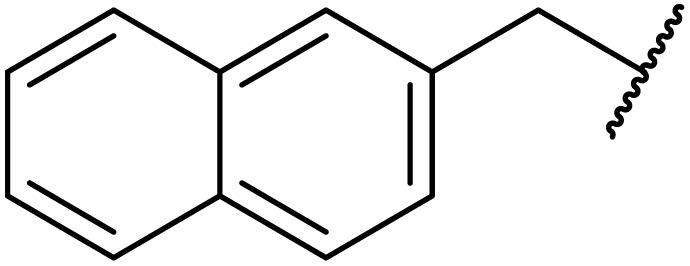	Br	C	THP	2	n/a	81%
22^b^	MeO_2_C(CH_2_)_10_	Br	C	Dioxane	24	n/a	77%
23^c^	*n*-BuHNOC(CH_2_)_10_	Br	C	THP	24	n/a	80%
24	MeO_2_CC_6_H_4_CH_2_	Cl	C	THP	3	n/a	80%

a 1 M methanolic HCl used.

b 3.01 eq. bicarbonate AER and no iodide resin.

c 2.91 eq. bicarbonate AER and no iodide resin.

The squarate dianion (Sq^2−^) was investigated by Tanino and co-workers as a “soft”, non-basic oxygen nucleophile for the transformation of alkyl halides into alcohols.^102^ Initial *in situ* deprotonation of squaric acid (H_2_Sq) affords the Sq^2−^ dianion, which undergoes S_N_2 substitution of the halide to generate the squarate monoester. The monoester is then subjected to basic hydrolysis to the desired alcohol in a one-pot procedure (Table 24). These conditions afforded excellent yields from primary halides (entry 1) and good yields from secondary halides (entries 2–4). The reaction of benzyl halides was not majorly influenced by ring substituents (entries 5–10). This procedure demonstrated good chemoselectivity, with multihalogenated substrates converted to their monohydroxy products *via* substitution of the more reactive halogen (entries 15–16). Increasing the equivalents of the dianion provided access to diols in high yields (entries 17–18).

**Table 24:** Preparation of alcohols *via* squarate intermediates

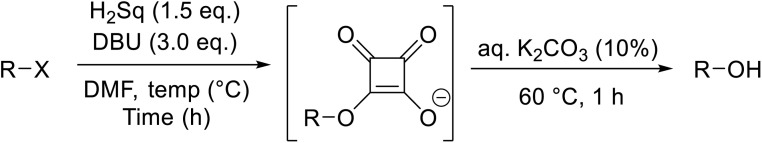						
Entry	R	X	Temp (°C)	Time (h)	Product	Yield
1	BnO(CH_2_)_3_	Cl	80	2	BnO(CH_2_)_3_OH	91%
2	BnO(CH_2_)_2_CHMe	Br	80	2	BnO(CH_2_)_2_CHOH(Me)	76%
3	BnO(CH_2_)_2_CHMe	Cl	90	3	BnO(CH_2_)_2_CHOH(Me)	70%
4	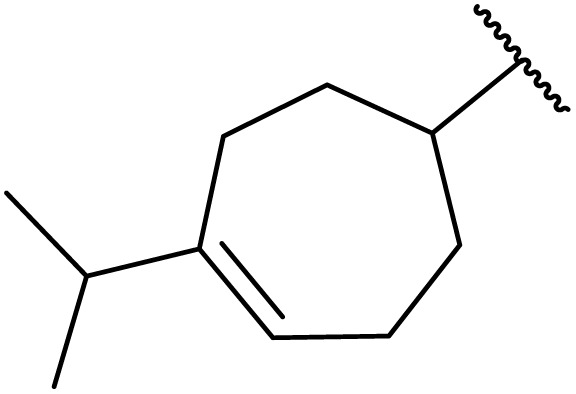	Cl	120	6	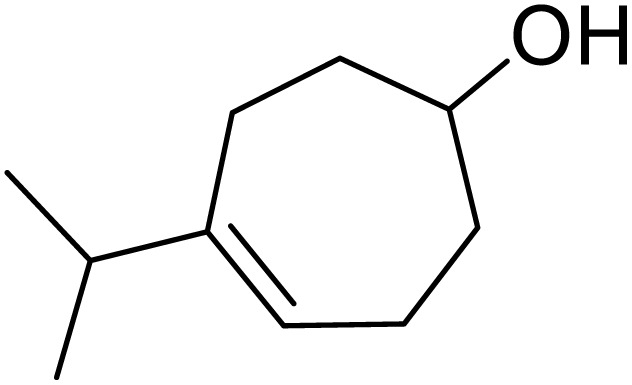	64%
5	Bn	Br	60	0.5	BnOH	71%
6	4-MeOC_6_H_4_CH_2_	Cl	60	0.5	4-MeOC_6_H_4_CH_2_OH	96%
7	4-MeC_6_H_4_CH_2_	Br	60	0.5	4-MeC_6_H_4_CH_2_OH	69%
8	4-BrBn	Br	60	0.5	4-BrBnOH	74%
9	4-NCC_6_H_4_CH_2_	Br	60	0.5	4-NCC_6_H_4_CH_2_OH	87%
10	O_2_NC_6_H_4_CH_2_	Br	60	0.5	O_2_NC_6_H_4_CH_2_OH	66%
11	Cinnamyl	Br	60	0.7	Cinnamyl alcohol	57%
12	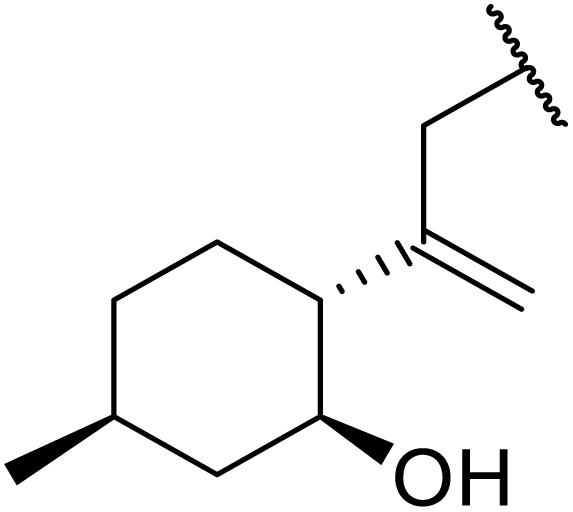	Cl	80	2	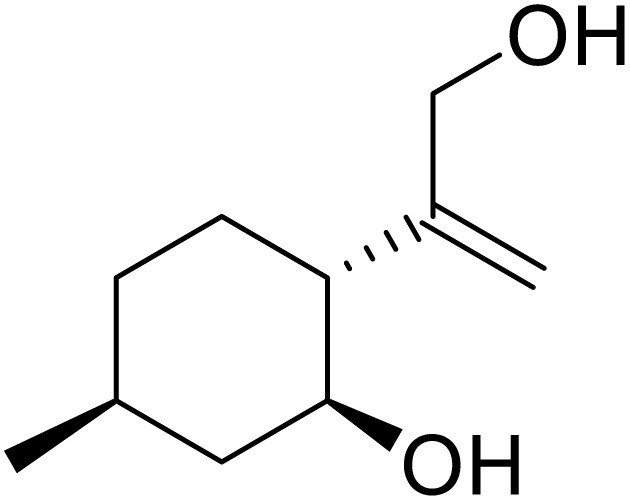	79%
13	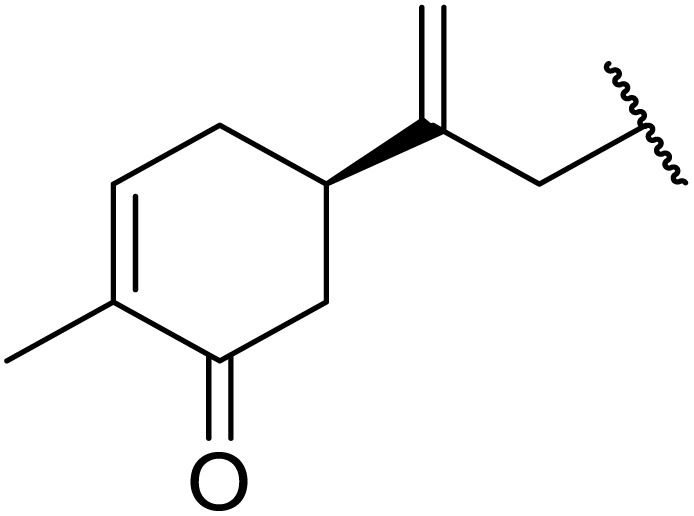	Cl	80	2	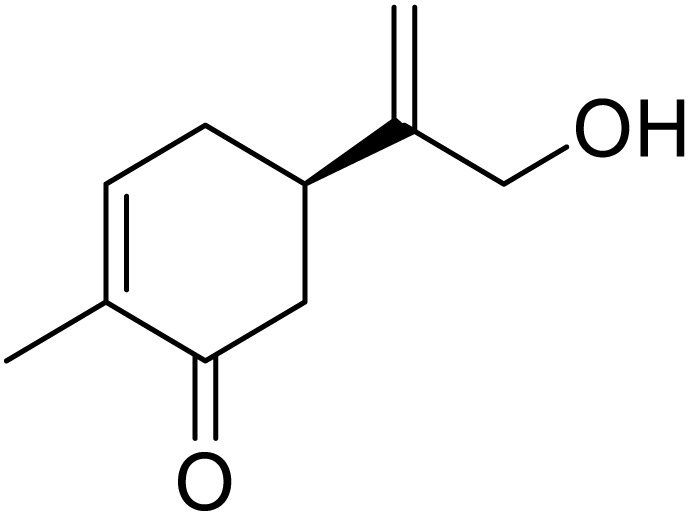	71%
14	Me(CH_2_)_4_C≡CCH_2_	Cl	80	1	Me(CH_2_)_4_C≡CCH_2_OH	86%
15	Cl(CH_2_)_6_	I	r.t.	2	Cl(CH_2_)_6_OH	87%
16	Cl(CH_2_)_7_	Br	r.t.	6	Cl(CH_2_)_7_OH	73%
17^a^	Cl(CH_2_)_7_	Br	80	2	HO(CH_2_)_7_OH	85%
18^a^	TsO(CH_2_)_7_–	Br	60	1	HO(CH_2_)_7_OH	83%
19^b^	Me(CH_2_)_5_CH(Br)CH_2_	Br	120	2	Me(CH_2_)_5_CH(OH)CH_2_OH	59%

a 3.0 eq. H_2_Sq, 6.0 eq. DBU.

b 2.4 eq. H_2_Sq, 2.4 eq. DBU.

Linear fluorinated diols were obtained from their diiodide precursors by Baum and Malik following treatment with fuming sulfuric acid and subsequent hydrolysis of the intermediate sulfate esters.^108^ This procedure draws on previous work by Mares and Oxenrider.^109^ The target diols were isolated in high yields (Table 25, entries 1–3).

**Table 25:** Hydroxylation of perfluoroalkyl iodides with fuming sulfuric acid and subsequent hydrolysis

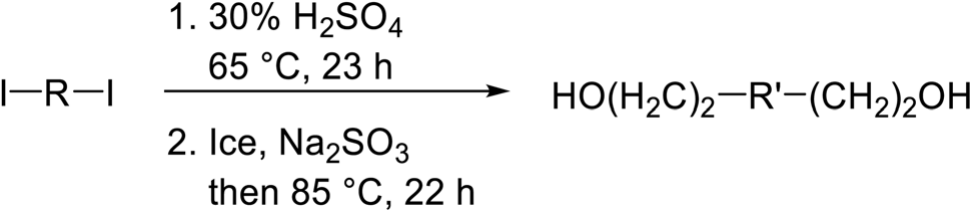			
Entry	R	R′	Yield
1	–(CH_2_)_2_(CF_2_)_5_CF(CF_3_)(CH_2_)_2_–	–(CF_2_)_5_CF(CF_3_)–	83%
2	–(CH_2_)_2_[CF(CF_3_)CF_2_]_2_(CF_2_)_4_[CF(CF_3_)CF_2_]_2_(CH_2_)_2_–	–[CF(CF_3_)CF_2_]_2_(CF_2_)_4_[CF(CF_3_)CF_2_]_2_–	76%
3	–(CH_2_)_2_[CF(CF_3_)CF_2_]_3_(CF_2_)_4_[CF(CF_3_)CF_2_]_3_(CH_2_)_2_–	–[CF(CF_3_)CF_2_]_3_(CF_2_)_4_[CF(CF_3_)CF_2_]_2_–	73%

**Scheme 8:**
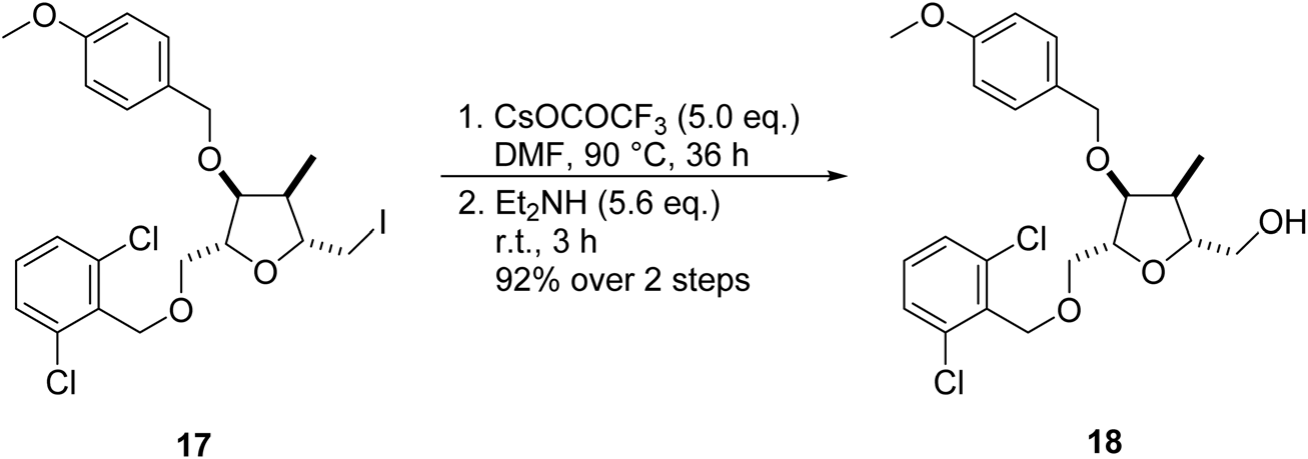
Use of caesium trifluoracetate in the synthesis of gymnodimine.

An application of the hydrolysis of intermediate esters to access alcohols can be seen in the synthesis of gymnodimine, a naturally-occurring cytotoxic compound.^110^ The iodide in 17 was first displaced by trifluoroacetate which was subsequently cleaved by diethylamine to furnish alcohol 18 in 92% yield (Scheme 8). Wang *et al.* successfully adopted a similar strategy in their synthesis of iriomoteolide-2a, a marine macrolide.^111^

### 2.8 Photochemical methods

Photochemical methods are often characterised by excellent atom efficiency, high sustainability and versatile scalability.^112–115^ Many newly developed methods display wide functional group tolerance, utilise mild reaction conditions and completely avoid hazardous or toxic reagents. Advances in LED technology and flow chemistry have further driven the explosion of interest in photochemical techniques.^116,117^ Although many examples of photo-oxygenation are reported in the literature,^118,119^ only a limited number of photocatalytic transformations of alkyl halides to alcohols have been published to date. These often proceed in poor to moderate yields in the case of oxygen radical-driven processes^120^ or have limited substrate scope, as in the case of organoantimony radical-driven processes.^121^

**Scheme 9:**
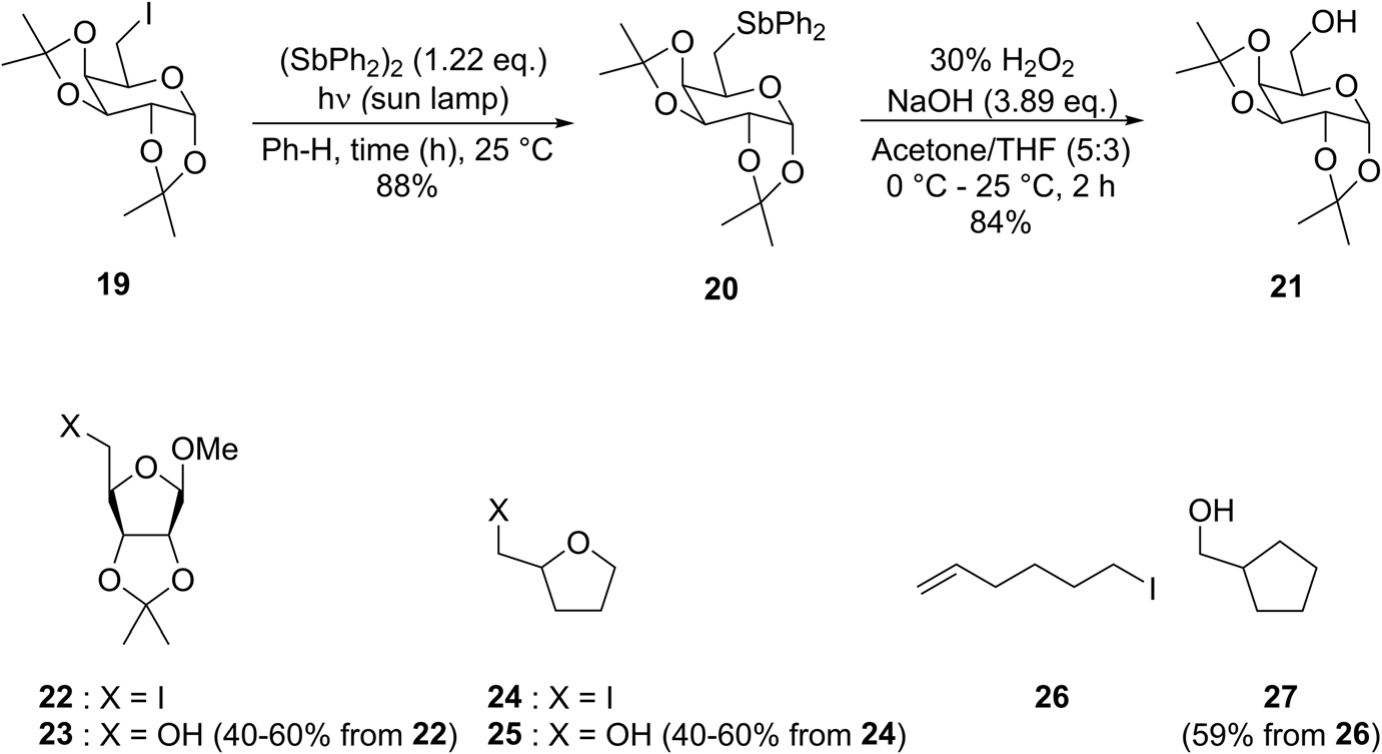
Photochemical conversion of alkyl iodides using tetraphenyldistibine as a radical initiator.

Barrett and Melcher investigated tetraphenyldistibine as a radical initiator for the conversion of heterocyclic alkyl iodides to their corresponding alcohols.^121^ Irradiation of tetraphenyldistibine produced the diphenylantimony radical, which reacted with alkyl iodide 19*via* a radical process to afford intermediate alkyl(diphenyl)stibine 20 (Scheme 9). Peroxide-mediated oxidation under basic conditions furnished alcohol 21 in 84% yield. Conversion of tetrahydrofuran 22 to the alkyl(diphenyl)stibine intermediates was more sluggish, but afforded alcohol 23 by simple air oxidation. Tetrahydrofuran 24 reacted in a similar manner. This approach also facilitated more complex chemistry, such as the cyclisation of unsaturated alkyl iodide 26 to alcohol 27.

The photoinduced hydroxylation of alkyl halides under mild conditions was examined by Zhou and co-workers.^120^ Although their primary focus was on aryl halides, several alkyl halides were converted to their corresponding alcohols in modest to good yields (Table 26). Hydroxylations were conducted in acetonitrile under an oxygen atmosphere and UV irradiation. The presence of catalytic sodium iodide was crucial, leading to a doubling of yields. Both primary alkyl iodides (entry 1) and bromides (entry 2) underwent hydroxylation in good yields. The efficiency of the reaction decreased significantly, however, in the case of secondary (entries 3–5) or tertiary (entries 6–8) substrates, while benzyl halides were moderately reactive (entries 9–10).

**Table 26:** Photoinduced hydroxylation of alkyl halides

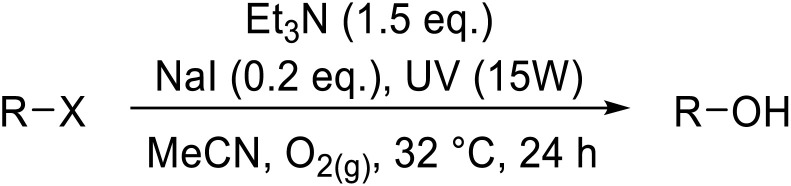			
Entry	R	X	Yield
1^a^	*n*-Decyl	I	66%
2	*n*-Dodecyl	Br	50%
3	Me(CH_2_)_6_CHMe	I	26%
4	Me(CH_2_)_6_CHMe	Br	15%
5	Me(CH_2_)_6_CHMe	Cl	Trace
6	Me(CH_2_)_6_C(Me)_2_	I	6%
7	Me(CH_2_)_6_C(Me)_2_	Br	11%
8	Me(CH_2_)_6_C(Me)_2_	Cl	Trace
9	Bn	I	41%
10	Bn	Br	35%

a Reaction conducted for 5 h in absence of NaI.

### 2.9 Metallation–oxidation methods

Metallation–oxidation typically involves halogen–metal exchange of an alkyl halide to generate an organometallic intermediate, which is subsequently oxidised to an alcohol. Formation of the reactive intermediate is typically conducted using magnesium metal or *n*-butyllithium, the latter being notoriously pyrophoric which can cause handling difficulties.^122^ Reaction conditions for this chemistry demand cryogenic temperatures,^123,124^ although yields are generally good.

**Scheme 10:**
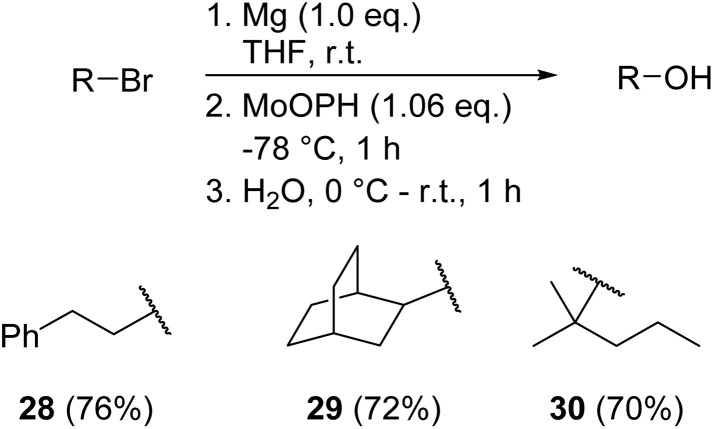
Conversion of alkyl bromides to alcohols *via* a Grignard intermediate.

The conversion of alkyl halides to alcohols *via* an organomagnesium intermediate was explored by Lewis and Gabhe.^123^ A Grignard intermediate was initially generated from the bromide precursor using magnesium turnings (Scheme 10). The reactive species was next treated with powdered molybdenum/pyridine/HMPA, also known as oxodiperoxymolybdenum(pyridine)-(hexamethylphosphoric triamide) or MoOPH, acting as an electrophilic oxidant.^125^ This system facilitated the preparation of primary, secondary and tertiary alcohols in good yields.

**Scheme 11:**
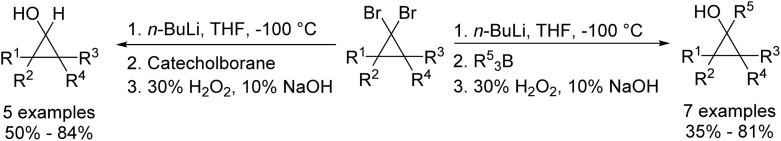
Preparation of alcohols *via* halogen–metal exchange.

Danheiser and Savoca adopted a somewhat similar strategy to access substituted cyclopropanols (Scheme 11).^124^ Low-temperature, halogen–metal exchange with 1,1-dibromocyclopropyl substrates using *n*-butyllithium afforded *gem*-lithiobromopropanes. Addition of cathecholborane resulted in an ate complex, which underwent Matteson-Pasto rearrangement upon warming to generate a cyclopropylborane.^126,127^ Alkaline peroxide-mediated oxidation of the cyclopropylborane derivative provided the desired alcohol.

Finally, Table 27 provides a high-level summary of the chemistry discussed in this section, capturing the key characteristics, advantages and disadvantages of the different strategies.

**Table 27:** Summary of approaches for the preparation of aliphatic alcohols from aliphatic halides

Approach	Characteristics	Advantages	Disadvantages
Reactions in water	Polar aprotic solvent increases nucleophilicity	Green solvent; compatible with RBr and RCl; 2° and 3° RX are suitable	Addition of polar aprotic solvent increases environmental impact; elevated temperatures required
Silver catalysis	High affinity for halides	High yielding; suitable for RCl and RBr; polyfunctional substrate-compatible	Silver salts often environmentally harmful; high loadings often required
Reactions with *m*CPBA	Rapid reaction times	High yielding for simple substrates	Abundant side product formation; only for RI
Oxygen-mediated reactions	Oxygen or superoxide	O_2_ is green and renewable oxidant; 2° and 3° RI conversions possible	Most examples use R_3_SnH; mostly for RI; no examples of RCl
Ionic liquid catalysis	Designer solvents	Compatible RBr and RCl; suitable for complex substrates; high yielding	Elevated temperatures
Mercury-assisted hydroxylations	HgO modified *in situ*	Mild/moderate temperatures; 2° and 3° RBr-compatible	Highly toxic; environmental hazard
Reactions with salts	Ester intermediate formed	High-yielding; RCl and RBr-compatible; sensitive functionality-compatible	Hydrolysis step generally required
Photochemical methods	Radical-driven	Green chemistry approach	Not suitable for RCl; low yielding
Metallation–oxidation methods	Halogen–metal exchange	3° RX-compatible; good yields	Cryogenic temperatures; pyrophoric reagents

## 3. Aliphatic thiols

The conversion of alkyl halides to aliphatic thiols is described in this section which has been further divided into the following subsections: 3.1 Inorganic sulfur sources, 3.2 Thioacetates and thioureas, 3.3 Thiocarbonic acid derivatives, 3.4 Heteroaryl thiones, and 3.5 Silylated sulfur sources.

### 3.1 Inorganic sulfur sources

Inorganic sulfur sources constitute the largest category of reagents employed for the conversion of alkyl halides to thiols, although the nature and structure of the sulfur source can vary widely. While hydrogen sulfide (H_2_S) or hydrosulfide-metal (MSH) salts are most commonly used, other effective reagents include thiocyanate,^128^ metal sulfides,^129^ sulfurated sodium borohydride,^130^ thiophosphate^131^ and carbon disulfide.^132^ Hydrogen sulfide is a highly toxic, flammable gas^133^ and has been largely superseded by safer alternatives such as thioacetate, thiourea and thiocarbonic acid derivatives. Additionally, hydrosulfide reagents tend to induce sulfide formation due to overalkylation.^134^ More recent innovations include solid support hydrosulfide exchange resins which produce thiols in high yields from the aliphatic halide precursors.^135,136^

**Scheme 12:**
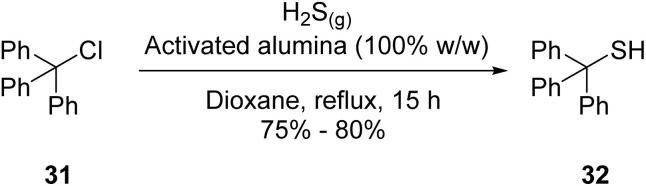
Alumina-assisted thiolation.

Building upon earlier precedent by Vorländer and Mittag,^137^ Kharasch and Williams investigated the hydrogen sulfide-mediated thiolation of triphenylmethyl chloride, but obtained yields of 50–60% at most.^138^ They subsequently achieved greater success by employing equal amounts of an activated alumina catalyst (Alcoa F-20) and starting material in anhydrous dioxane, affording the target thiol in 75–80% yield (Scheme 12).

In order to minimise sulfide formation, Bittell and Speier investigated the preparation of thiols and dithiols using hydrogen sulfide and ammonia or simple amines.^134^ This approach was compatible with both primary alkyl chlorides and bromides (Table 28, entries 1–5). Secondary substrates were also tolerated, although elimination to form cyclohexene was favoured over substitution in a 2.5 : 1 ratio (entry 7).

**Table 28:** Synthesis of thiols from alkyl halides, hydrogen sulfide and ammonia/amines

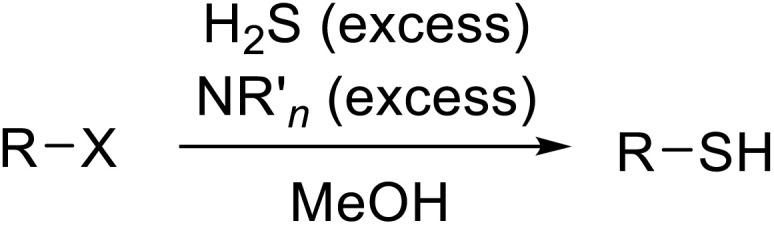						
Entry	Substrate		Base	Temp. (°C)	Time (h)	Yield
	R	X				
1	*n*-Dodecyl	Cl	NH_3_	120	22	81%
2	(MeO)_3_Si(CH_2_)_3_	Cl	NH_3_	100	18.5	88%
3	(MeO)_2_(Me)Si(CH_2_)_3_	Cl	NH_3_	100	4	90%
4	*n*-Hexyl	Cl	Et_3_N	75	6	79%
5	–(CH_2_)_2_–	Br	*n*-Pr_2_NH	r.t.	4	95%
6	Bn	Cl	NH_3_	0	1	92%
7	Cy	Br	Et_3_N	75	24	Not reported

A series of di- and trithiols were prepared by Simpson as potential antidotes to arsenic poisoning (Table 29).^139^ Two different hydrosulfide salts were used to effect the halide to thiol transformation, namely sodium hydrosulfide (Method A) or ammonium hydrosulfide (Method B) in ethanol. In general, the former proved superior to the latter (entries 2–5, 9). Reactions involving alkyl chlorides necessitated longer times or more forcing conditions (entries 5–8).

**Table 29:** Synthesis of di- and trithiols from alkyl halides

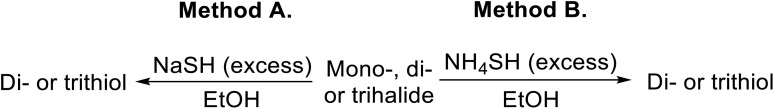						
Entry	Substrate	Method	Temp. (°C)	Time (h)	Product	Yield
1	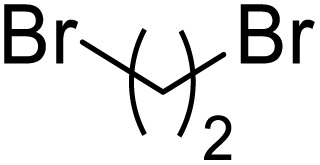	A	30–60	172	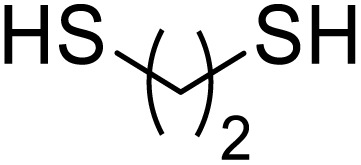	17%
2	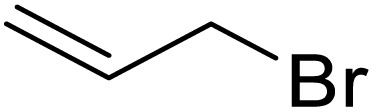	A	25	120	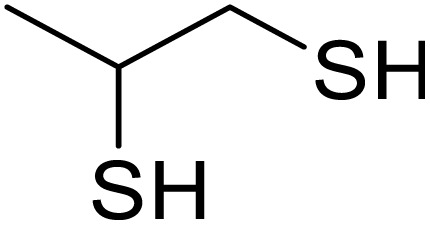	32%
		B	80	8		30%
3	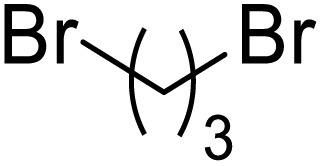	A	30	168	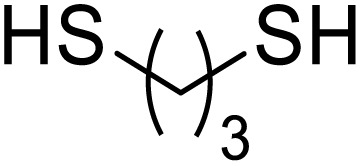	53%
		B	70	8		20%
4	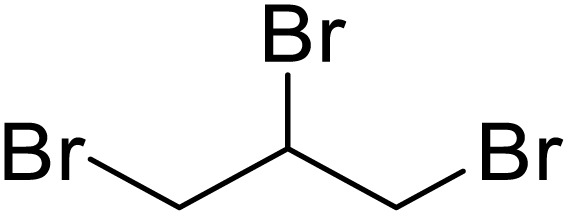	A	60	6	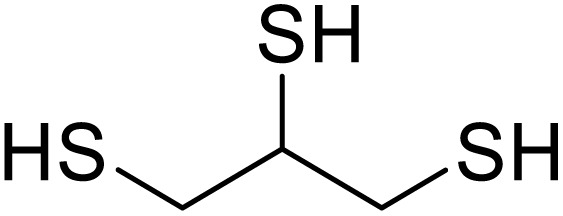	43%
		B	60	12		10%
5	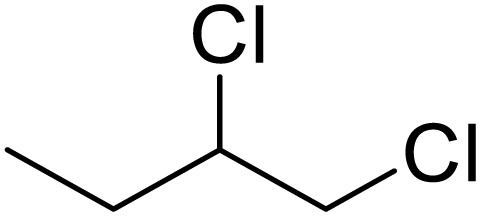	A	80	12	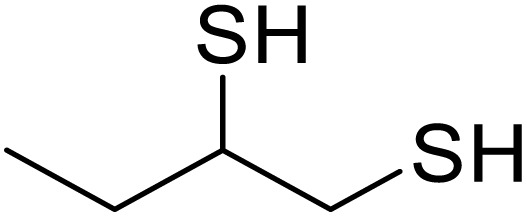	25%
		B	25–30	168		22%
6	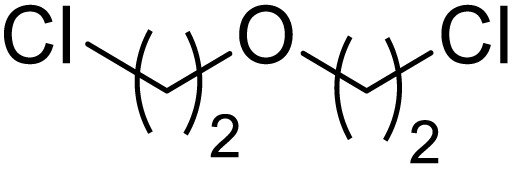	A	30	168	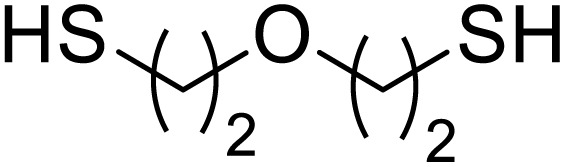	20%
		B	80	12		35%
7	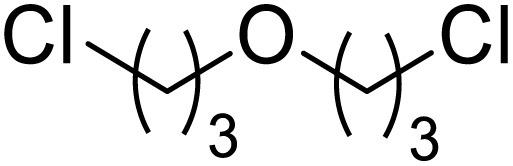	B	70	5	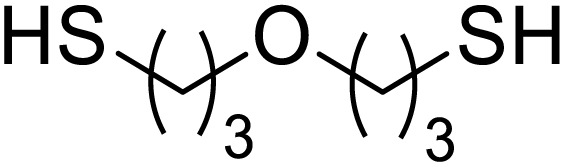	47%
8	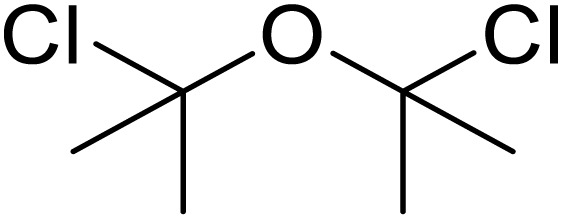	A	30–60	342	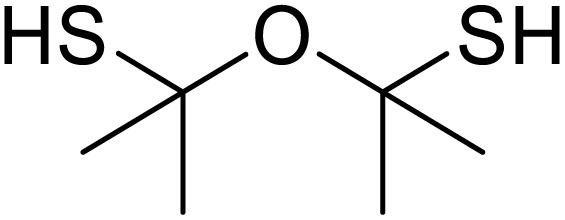	11%
9	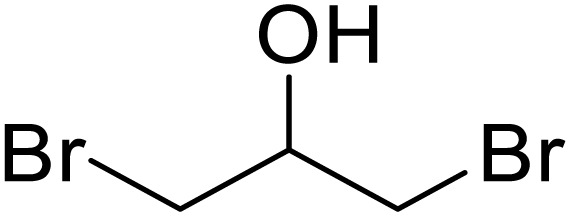	A	30–60	74	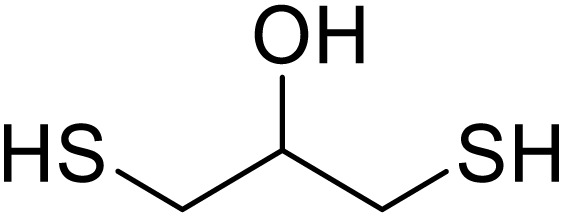	40%
		B	80	12		26%

**Scheme 13:**
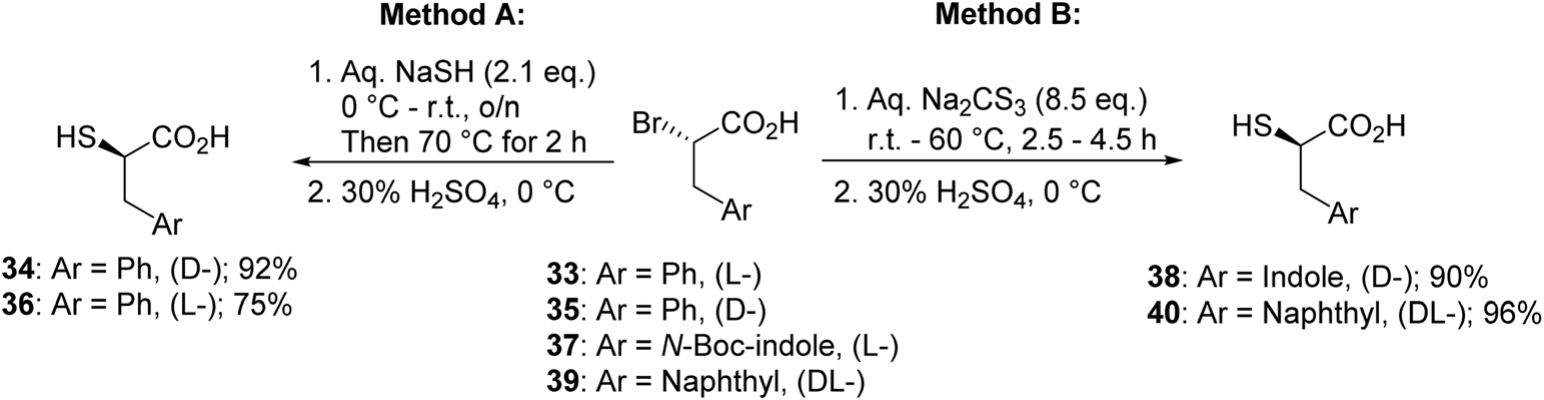
Preparation of chiral α-thio acids.

Samuels and Sevrioukova developed a facile approach for the preparation of α-thio acids from aromatic amino acids.^140^ These compounds represent key intermediates in the synthesis of cytochrome P450 inhibitors.^141^ Following initial diazotisation and subsequent bromination, the intermediate α-bromocarboxylic acids were converted to their respective α-thio acids in mostly excellent yields using aqueous sodium hydrosulfide (Scheme 13). For certain substrates, the use of more nucleophilic sodium trithiocarbonate, followed by acidic hydrolysis, afforded better outcomes. The reactions typically proceeded with inversion at the α-position.

Advancing prior work by Noller and Post,^142^ Cooper found that organosilicon mercaptans could be prepared from silylated alkyl chlorides and potassium hydrosulfide in ethanol.^143^ The sulfur transfer reagent was initially prepared from hydrogen sulfide and potassium hydroxide. The appropriate alkyl halide was then added over 20 min while hydrogen sulfide was continually passed through the reaction mixture to minimise sulfide formation. Thiol products were recovered in moderate yields (Table 30).

**Table 30:** Preparation of organosilicon mercaptans from alkyl chlorides

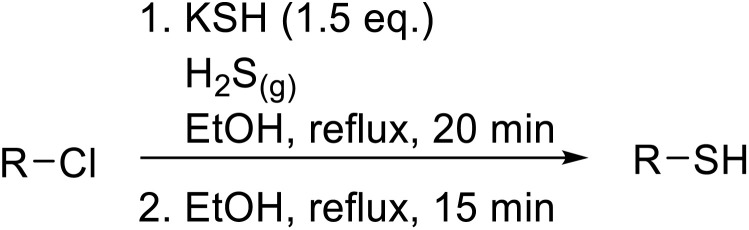		
Entry	R	Yield
1	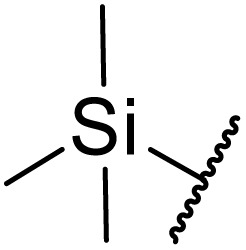	42%
2	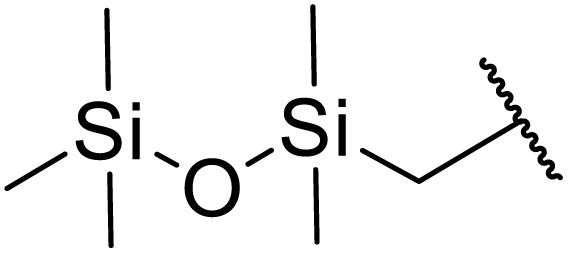	51%
3	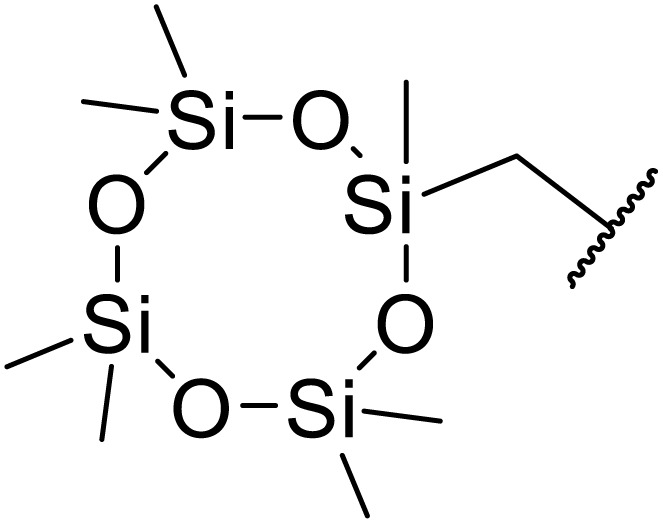	59%

**Scheme 14:**
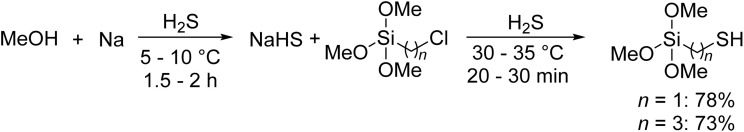
Use of sodium hydrosulfide in the synthesis of sulfurated organosilanes.

Voronkov *et al.* also studied the transformation of various organosilane substrates to sulfurated organosilanes.^144^ Sodium hydrosulfide was generated *in situ* by passing hydrogen sulfide through a solution of sodium in methanol (Scheme 14). Following addition of the appropriate trimethoxysilylalkyl chloride, the thiol was subsequently recovered in good yield. It was observed that insufficient hydrogen sulfide resulted in formation of bis(trimethoxysilylalkyl) sulfides as the major product.

Choi and Yoon sought to develop a new direct method for the preparation of thiols using hydrosulfide exchange resin.^135^ The resin is prepared by reaction of Amberlite IRA-400 chloride anion exchange resin and sodium hydrosulfide (Table 31). Addition of an equimolar amount of triethylammonium chloride was necessary to avoid unwanted sulfide formation. Unactivated primary alkyl bromides and iodides reacted smoothly (entries 1–2). Conversion of tosylates or secondary bromides required higher temperatures and equimolar sodium iodide (entries 3–5). Activated chlorides and halides were similarly compatible with this chemistry (entries 6–13). By contrast, aryl iodides were inert even with extended reaction times (entry 14).

**Table 31:** Direct synthesis of thiols using hydrosulfide exchange resin

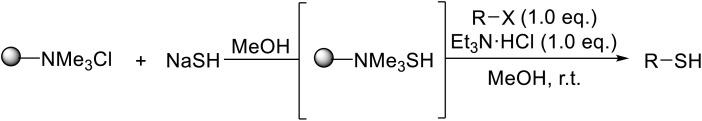				
Entry	R	X	Time (h)	Yield
1	*n*-Octyl	Br	6	85%
2	*n*-Octyl	I	1	87%
3	*n*-Octyl	OTs	3	a ^,^ b94%
4	*n*-BuCH(Me)	Br	6	b68%
5	Me(CH_2_)_5_CH(Me)	OTs	6	b70%
6	Bn	Cl	1	87%
8	Bn	Br	1	88%
9	PhCH=CHCH_2_	Cl	1	91%
10	PhCOCH_2_	Br	1	90%
11	EtO_2_CCH_2_	Cl	1	a97%
12	EtO_2_CCH(Me)	Cl	3	92%
13	H_2_NOCCH_2_	Cl	1	c90%
14	Ph	I	6	c0%

a Yield determined by gas chromatography.

b 50 °C in the presence of equimolar NaI.

c 50 °C.

A similar strategy was adopted by Bandgar and Pawar using Tulsion A-27 exchange resin treated with sodium hydrosulfide in methanol.^136^ The resulting hydrosulfide exchange resin proved more nucleophilic than sodium hydrosulfide, allowing for thiolation of alkyl halides at 25 °C (Table 32). The products were isolated in excellent yields following filtration and evaporation (entries 1–8). Dithiols were also accessible from the corresponding alkyl dibromides (entry 9). The resin was readily recycled after regeneration using dilute hydrochloric acid.

**Table 32:** Thiolation of alkyl halides using hydrosulfide exchange resin

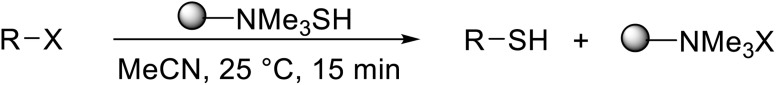			
Entry	Substrate		Yield
	R	X	
1	*n*-Bu	Br	98%
2^a^	*n*-Bu	I	93%
3	*t*-Bu	Br	96%
4	*t*-Bu	Cl	95%
5	Bn	Cl	98%
6	4-ClC_6_H_4_CH_2_	Cl	93%
7	2,4-Cl_2_C_6_H_3_CH_2_	Cl	97%
8	4-O_2_NC_6_H_4_CH_2_	Cl	97%
9	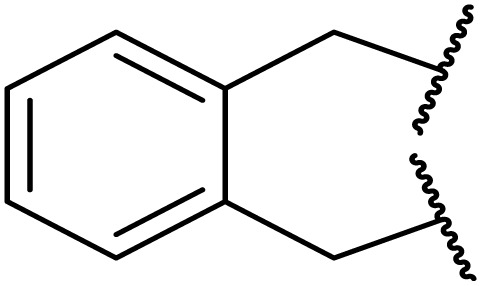	Br	96%

a Reaction conducted at 0 °C.

**Scheme 15:**
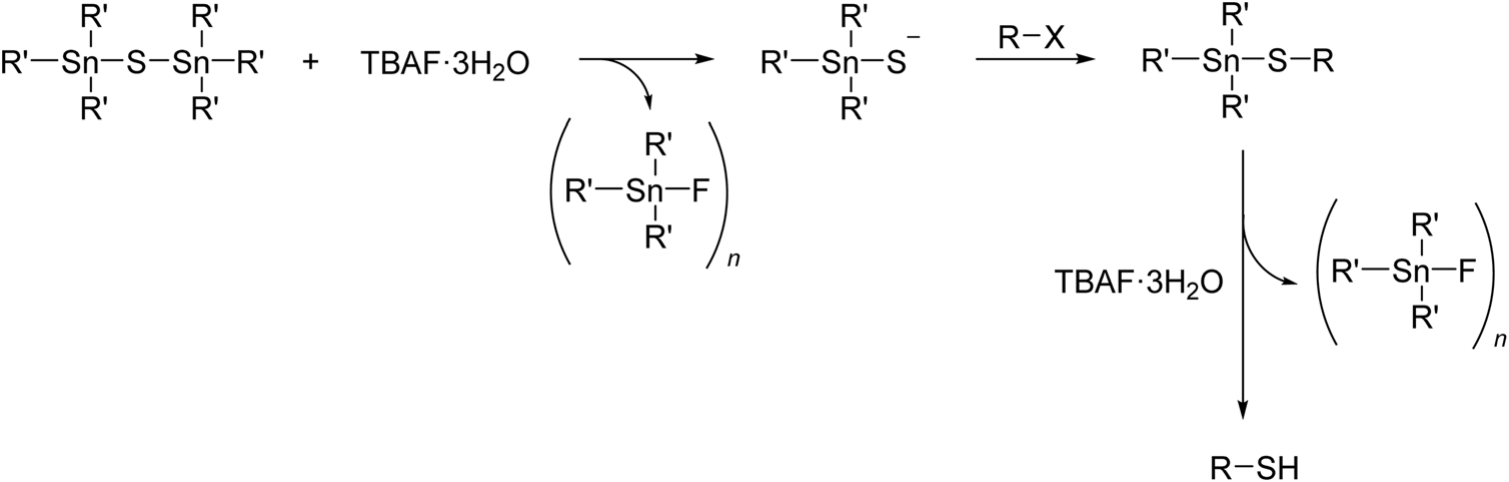
Mechanism of tin-mediated thiolation.

Harpp and Gingras prepared thiols using triorganotin mercaptan intermediates as sulfur transfer agents.^129^ Initial fluorodestannylation of a bis(organotin) sulfide with tetrabutylammonium fluoride (TBAF) generates a thiolate which reacts with an alkyl halide to produce an organotin sulfide (Scheme 15). A second fluorodestannylation cleaves the sulfur–tin bond to afford the alkyl thiol.

Various alkyl bromides were subjected to this one-pot methodology with the target thiols recovered in low to good yields (Table 33, entries 1–4). Small amounts of thioether formation were observed but they were subsequently removed by flash chromatography. Destannylation with tetrabutylammonium fluoride was superior to caesium fluoride/tetrabutylammonium iodide (entry 1 *vs.* 2).

**Table 33:** Synthesis of alkyl thiols using bis(triorganotin) sulfides

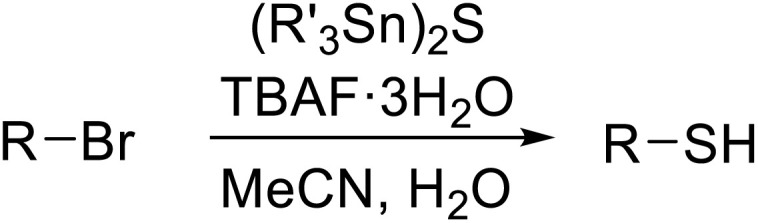					
Entry	R	R′	Temp. (°C)	Time (h)	Yield
1^a^	*n*-Hexyl	Ph	50	2.5	36%
2	*n*-Octyl	Bu	40	21	59%
3^b^	*n*-Octyl	Bu	20	20	71%
4	BnCH_2_	Bu	20	40	82%

a Destannylation performed using 1.51 eq. of CsF/TBAI.

b Substrate added *via* syringe pump over 4 h.

Brindle and Liard discovered that sodium borohydride sulfide, or sulfurated sodium borohydride, reacts with benzylic and aliphatic halides to produce high yields of α-toluyl/aliphatic thiols (Table 34).^145^ This reagent can be prepared by addition of sulfur powder to sodium borohydride.^130^ Good to excellent yields were generally obtained from benzylic and aliphatic halides (entries 1–5, 7) and this extended to dihalogenated substrates (entry 9). However, benzylic halides with strongly electron-withdrawing substituents saw poor conversion to the corresponding thiols (entries 10, 11). Increasing steric bulk was found to significantly hinder the reaction (entries 6, 8).

**Table 34:** Synthesis of thiols using sodium borohydride sulfide

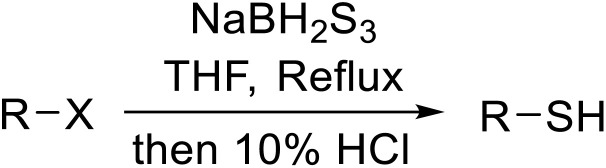						
Entry	Substrate		NaBH_2_S_3_ (eq.)	Temp. (°C)	Time (h)	R–SH yield
	R	X				
1	Bn	Br	4	20	16	75%
2	Bn	F	4	65	16	99%
3	Bn	Cl	4	65	16	99%
4	Bn	I	4	65	16	95%
5	Me	I	1	20	64	70%
6	*t*-Bu	Cl	4	65	16	Complex mixture
7	BnCH_2_	Cl	4	65	16	90%
8	PhCH(Me)	Br	4	65	16	30%
9	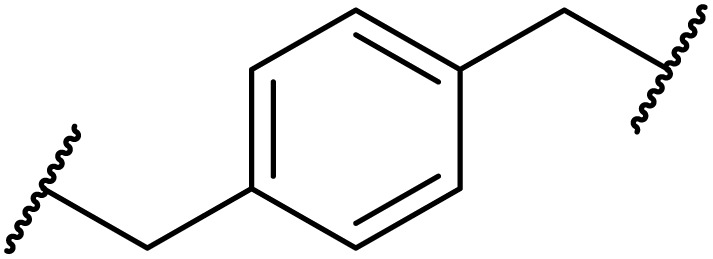	Cl	8	65	16	80%
10	4-O_2_NC_6_H_4_CH_2_	Cl	1	20	16	53%
11	4-O_2_NC_6_H_4_CH_2_	Br	1	20	16	Trace

Sodium thiophosphate was identified by Bieniarz and Cornwell as a useful sulfur source which facilitated thiol formation under benign conditions.^131^ Reaction with alkyl halides generated *S*-alkyl phosphorothioates which could be converted to thiols under mild acidic hydrolysis (Method A) or methanolysis (Method B) (Table 35). Conversion of both alkyl bromides (entries 1–5) and chlorides (entries 10–11) was successful, in addition to activated aryl and heteroaryl bromides (entries 6–7). The introduction of dibrominated substrates provided access to heterocyclic products (entries 8–9). More recently, Gupta *et al.* have adapted this chemistry to the preparation of polystyrene thiol resins.^146^

**Table 35:** Sodium thiophosphate as a sulfur source

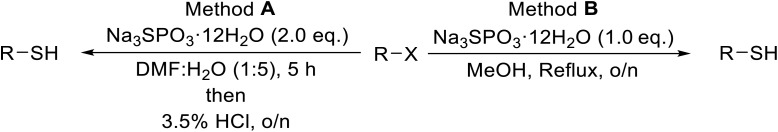					
Entry	R	X	Method	Product	Yield
1	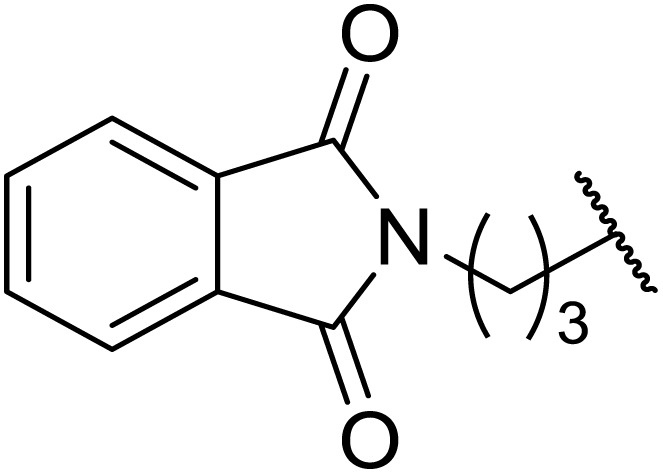	Br	A	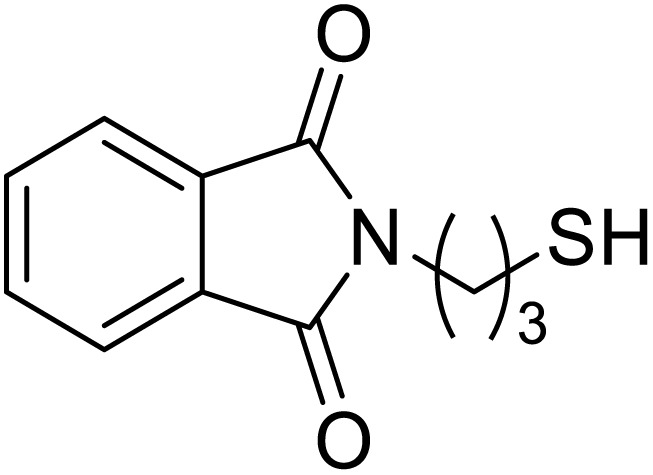	100%
2	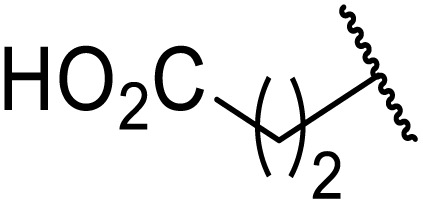	Br	A	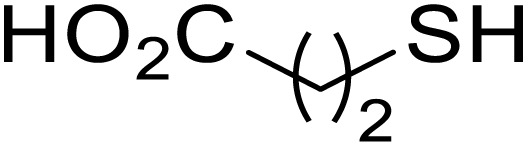	100%
3	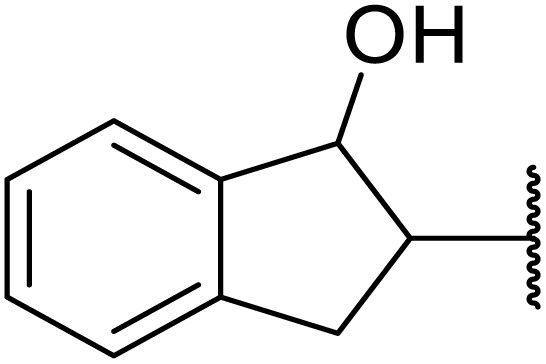	Br	A	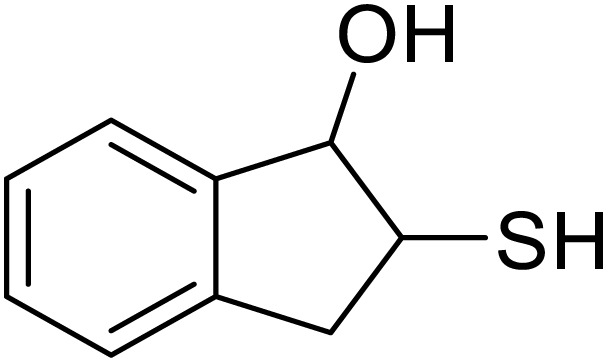	97%
4	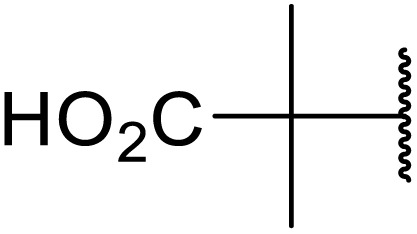	Br	A	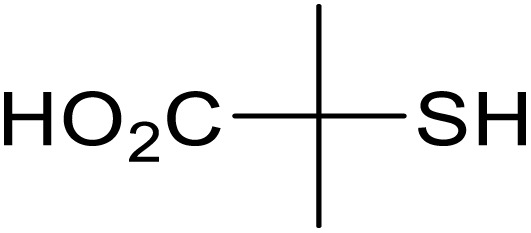	22%
5	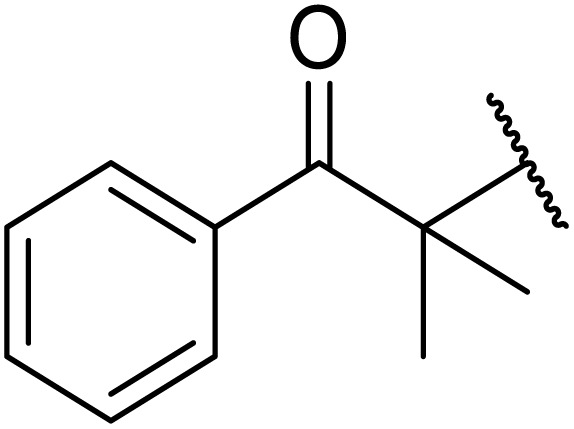	Br	A	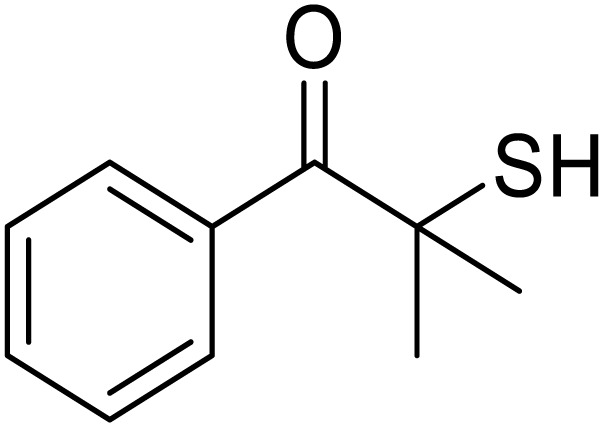	78%
6	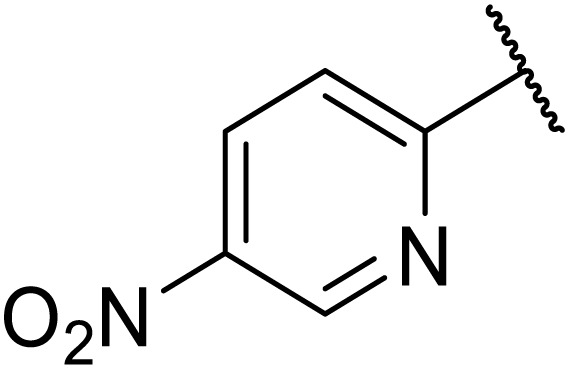	Br	B	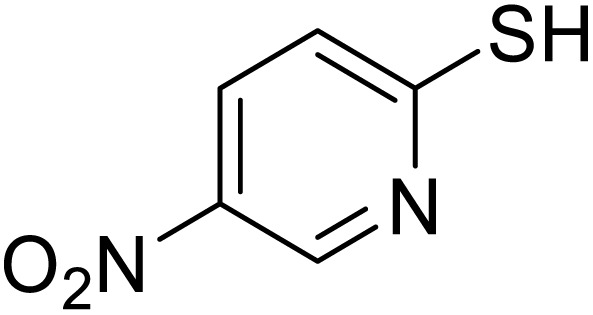	94%
7	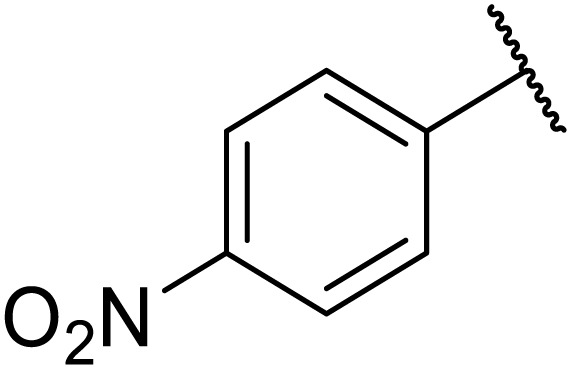	Br	B	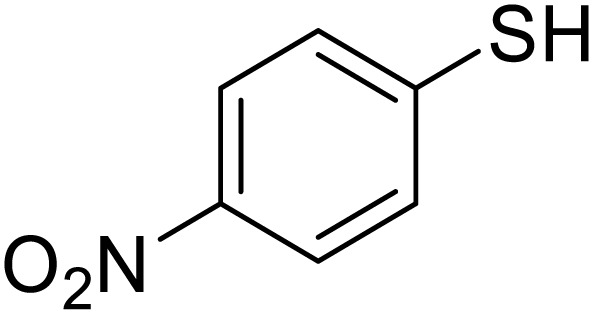	80%
8	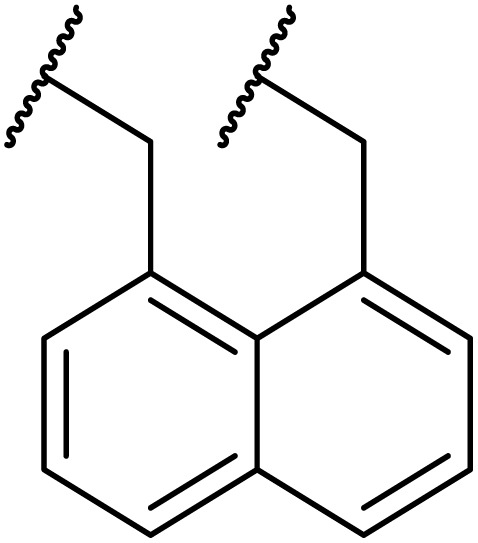	Br	B	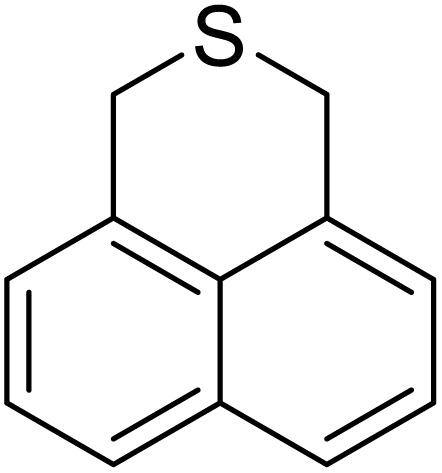	95%
9	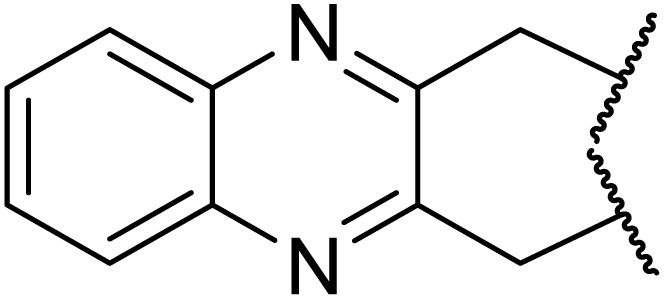	Br	B	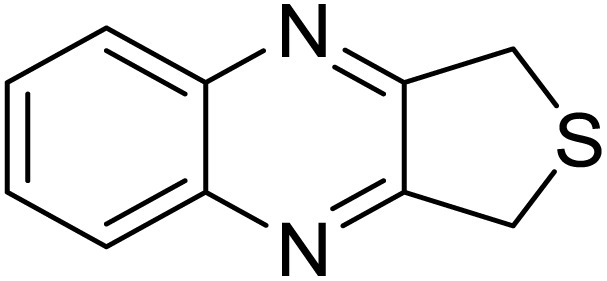	34%
10	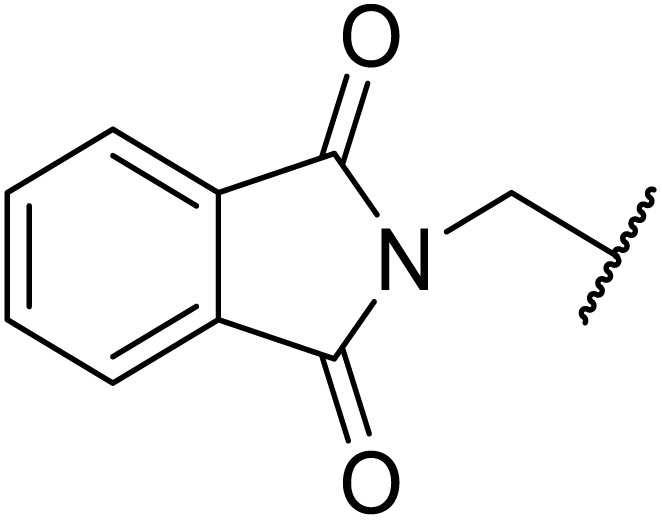	Cl	A	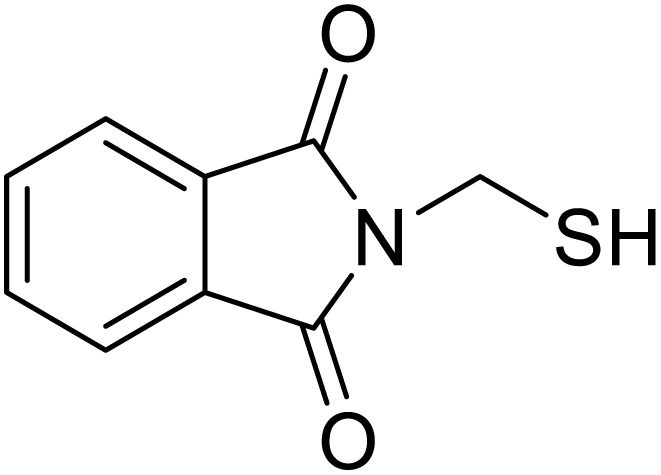	91%
11	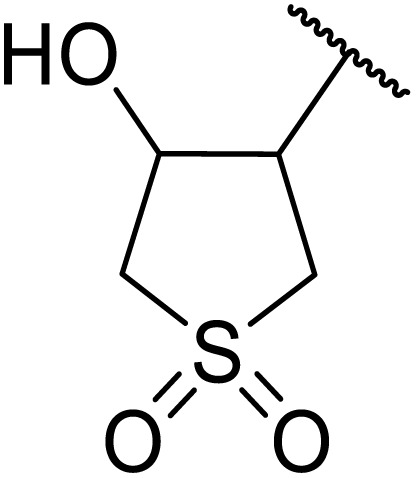	Cl	A	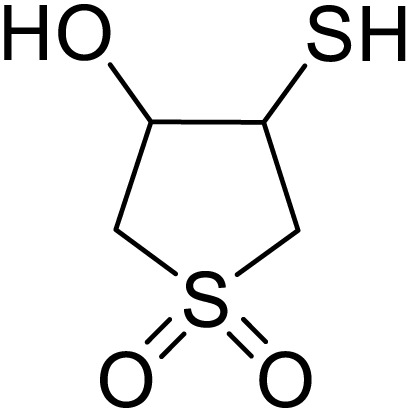	28%

Mironov and Pogonkina exploited thiocyanate salts for the preparation of organosilicon thiols from alkyl halide precursors.^128^ Organosilicon alkyl chlorides were first treated with sodium thiocyanate to afford the corresponding alkyl thiocyanate intermediates (Table 36). Following reaction with iso-propylmagnesium bromide generated *in situ*, the target organosilicon thiols were obtained in generally good yields.

**Table 36:** Preparation of organosilicon alkyl thiols *via* thiocyanate intermediates

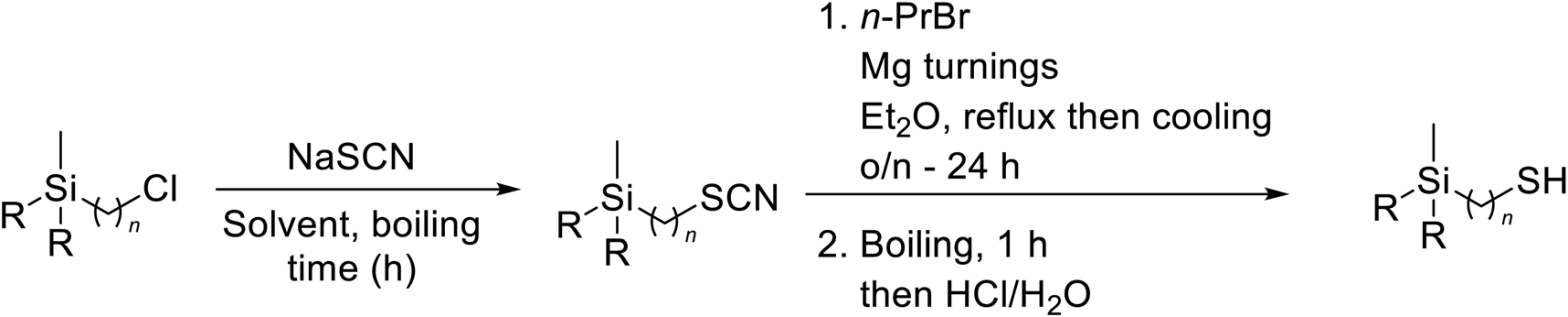									
Entry	R	*n*	NaSCN eq.	Solvent	Time (h)	Thiocyanate yield	*n*-PrBr eq.	Mg eq.	Thiol yield
1	Me	1	1.2 eq.	EtOH	10	84%	3.0	3.0	72%
2	Et	1	1.2 eq.	EtOH	35	89%	3.0	3.0	73%
3	*n*-Pr	1	1.2 eq.	Acetone	70	60%	3.0	3.0	68%
4	*n*-Bu	1	1.2 eq.	Acetone	105	66%	3.0	3.0	87%
5	Me	3	1.1 eq.	EtOH	52	72%	3.3	2.7	39%

**Scheme 16:**
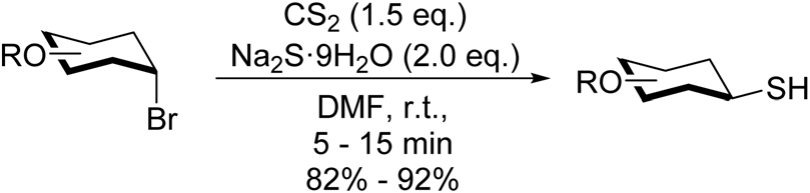
Preparation of β-glycosyl thiols.

An efficient protocol for the stereoselective preparation of β-glycosyl thiols from their α-glycosyl bromide precursors was reported by Misra and Jana (Scheme 16).^132^ The reaction proceeds *via* S_N_2 substitution of the bromide with a sodium carbonotrithioate species generated *in situ* from the reaction of sodium sulfide and carbon disulfide. DMF was found to be the optimal solvent. Additionally, an excess of carbon disulfide was necessary to prevent bis-glycosyl sulfide side product formation.

### 3.2 Thioacetates and thioureas

Thioacetates and thioureas represent valuable thiol-transfer reagents, and are widely employed in novel synthetic methodologies and in the total synthesis of complex, highly functionalised targets.^147–151^ Typically, an alkyl halide substrate is treated with the thioacetate/thiourea and converted to the intermediate thioester/isothiouronium salt, usually at mild to moderate temperatures.^152^ A hydrolysis step is then required to furnish the desired thiol. The conditions required to effect the hydrolysis can be modified to meet functional group compatibility or other requirements. While a larger number of synthetic steps is generally considered undesirable, the second step is usually almost quantitative and does not entail long reaction times. Although the majority of examples focus on the conversion of primary alkyl/benzyl halides, secondary alkyl halides have been successfully converted to their corresponding thiols in high yields.^153,154^

The transformation of benzylic bromides to their corresponding thiols *via* thioester intermediates has been studied by Han and Balakumar.^155^ Two slightly different methods were developed which afforded comparable yields (Table 37). In Method A, thioacetic acid and equimolar base were first added to the benzyl bromide, followed by a second equivalent of base to effect thioester hydrolysis (entries 1–4). For Method B, 2.2 equivalents of base were added at the start of the reaction, allowing for single step conversion to the thiol (entries 5–8). Methanol was subsequently added to initiate the de-esterification process. In general, Method B was suitable for benzylic halides having limited solubility in methanol. The preparation of dithiols was feasible with this approach (entries 4, 8). Some applications of this chemistry include solid-phase synthesis,^156^ preparation of sirtuin inhibitors,^147^ and polymer science.^148^

**Table 37:** Synthesis of benzyl thiols from benzyl bromides and thioacetate

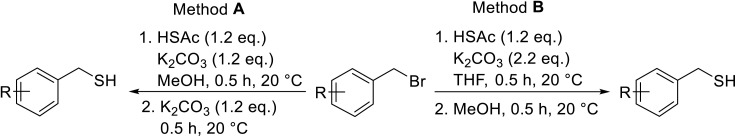				
Entry	R (substrate)	Method	R (product)	Yield
1	H	A	H	96%
2	2-Ph	A	2-Ph	98%
3	4-Br	A	4-Br	99%
4^a^	4-BrCH_2_	A	4-HSCH_2_	97%
5	H	B	H	94%
6	2-Ph	B	2-Ph	99%
7	4-Br	B	4-Br	97%
8^b^	4-BrCH_2_	B	4-HSCH_2_	94%

a 4.8 eq. of base.

b 4.4 eq. of base.

Richardson and co-workers likewise achieved the conversion of halides to the corresponding thioesters under mild conditions (Table 38).^153^ Yields are provided for individual thioesters (entries 1–29), but not for the corresponding thiols. However, the authors do state that thioester hydrolysis proceeds in yields of 80–95%. Aliphatic bromides and chlorides readily react with potassium thioacetate in DMF in high yields (entries 1–7). For reactions conducted in acetone, primary bromides (entries 12, 14–19, 21, 22), primary iodides (entries 23, 29), secondary bromides (entry 20), benzylic halides (entries 10, 25), allylic (entry 24) and α-halocarbonyl substrates (11, 26–28) afford good to excellent yields of the target thioacetate ester. Conversely, primary chlorides in acetone are not fully converted to the corresponding thioester and so DMF should be utilised as the reaction solvent (entries 1 *vs.* 8). Interestingly, this strategy is useful for selectively thiolating the more reactive halide in a multihalogenated substrate, as per entries 21–23.

**Table 38:** Preparation of thioesters as thiol precursors

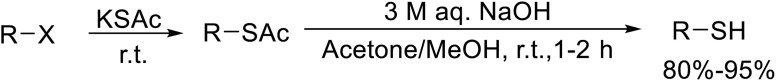					
Entry	R	X	Solvent	Time (h)	Yield (RSAc)
1	*n*-Octyl	Cl	DMF	96	84%
2	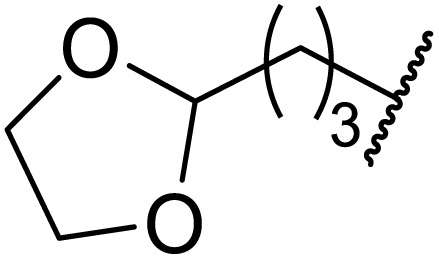	Cl	DMF	72	92%
3	*n*-Octyl	Br	DMF	17	88%
4	BnCH_2_	Br	DMF	16	92%
5	NC(CH_2_)_2_	Br	DMF	3	96%
6	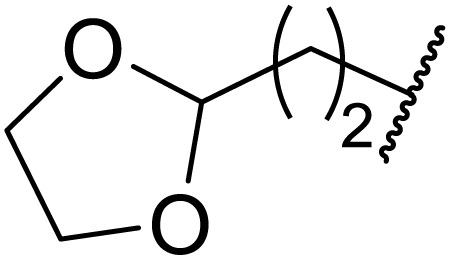	Br	DMF	46	86%
7	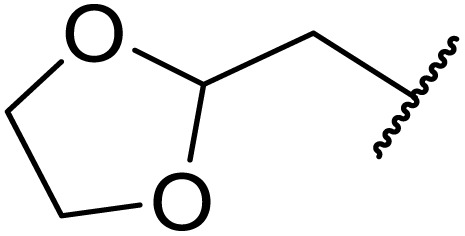	Br	DMF	67	77%
8	*n*-Octyl	Cl	Acetone	24	64%
9	*n*-Octyl	Cl	Acetonitrile	63	42%
10	Bn	Cl	Acetone	17	94%
11	MeCOCH(Me)	Cl	Acetone	20	95%
12	*n*-Decyl	Br	Acetone	26	92%
13	*n*-Decyl	Br	Methanol	69	88%
14	HO(CH_2_)_3_	Br	Acetone	14	91%
15	MeO(CH_2_)_2_	Br	Acetone	40	94%
16	–(CH_2_)_2_–	Br	Acetone	24	92%
17	EtO_2_C(CH_2_)_4_	Br	Acetone	40	96%
18	HO_2_C(CH_2_)_2_	Br	Acetone	7	93%
19	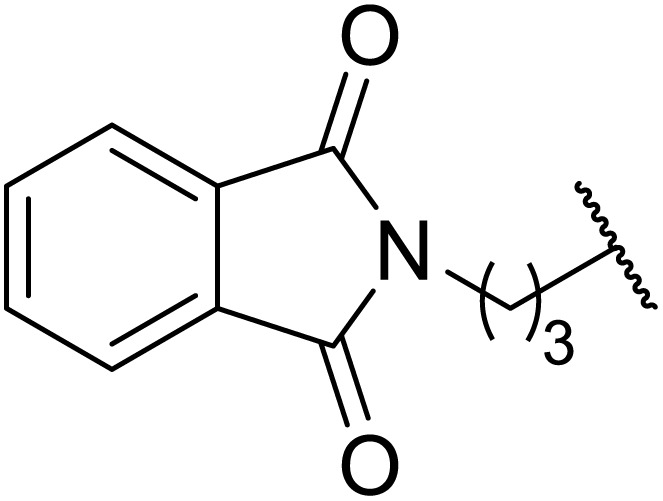	Br	Acetone	25	90%
20	EtCH(Me)	Br	Acetone	66	86%
21	Me(Br)CH(CH_2_)_3_	Br	Acetone	21	88%
22	ClCH_2_(Me)CHCH_2_	Br	Acetone	24	89%
23	Cl(CH_2_)_3_	I	Acetone	22	92%
24	H_2_C=CHCH_2_	Br	Acetone	18	90%
25	Ph(Me)CH	Br	Acetone	24	91%
26	PhCOCH_2_	Br	Acetone	17	96%
27	HO_2_CCH(*n*-Bu)	Br	Acetone	9	92%
28	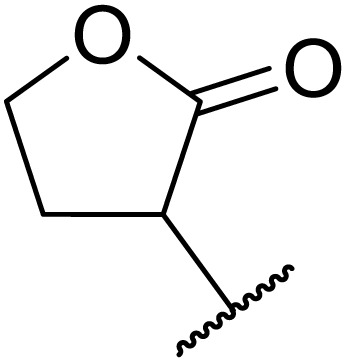	Br	Acetone	22	93%
29	*n*-Heptyl	I	Acetone	24	94%

**Scheme 17:**
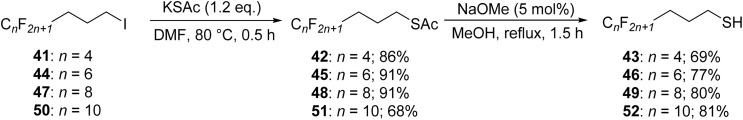
Potassium thioacetate as a thiolating agent.

Potassium acetate was employed by Rábai *et al.* as the thiolating agent for the preparation of (perfluoroalkyl)alkane thiols.^157^ (Perfluoroalkyl)alkane thiols, otherwise known as hemifluorinated alkyl mercaptanes, possess interesting physicochemical properties and have applications in nano- and materials chemistry.^158–160^ (Perfluoroalkyl)alkane iodides were initially treated with potassium acetate and a subsequent modified Zémplen deacetylation, using boiling methanol, furnished the target thiols in good yields (Scheme 17).^161^ Reactions generally proceeded smoothly and the thioacetylated intermediates were obtained in good yields with short reaction times.

Choi and Yoon developed a one-pot, two-step methodology for the conversion of alkyl halides to thiols.^154^ Following initial reaction of the alkyl halide with thioacetate exchange resin, subsequent Pd-catalysed methanolysis of the intermediate thioacetate affords the target thiol in excellent yields (Table 39). The addition of borohydride exchange resin accelerated the Pd-mediated methanolysis while also preventing disulfide formation. Whereas primary alkyl chlorides required extended reaction times (entry 1), primary alkyl bromides (entry 2), iodides (entry 3) and tosylates (entry 4) reached full conversion under four hours. By contrast, activated alkyl chlorides (entries 5, 6, 10) were readily converted to their thiols. The transformation of secondary alkyl bromides (entry 7), tosylates (entry 8) and iodides (entry 9) was also possible, albeit with longer reaction times.

**Table 39:** One-pot synthesis of thiols from alkyl halides under Pd-catalysis

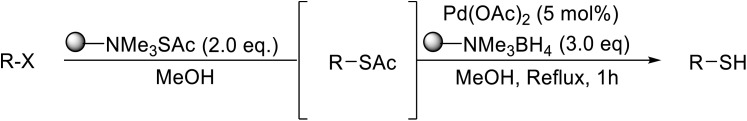					
Entry	Substrate		Temp. (°C)	Time (h)	Yield
	R	X			
1	*n*-Octyl	Cl	65	24	a87%
2	*n*-Octyl	Br	65	3	96%
3	*n*-Octyl	I	25	3	95%
4	*n*-Octyl	OTs	65	3	98%
5	Bn	Cl	25	1	94%
6	PhCH=CHCH_2_	Cl	25	1	97%
7	2-Hexyl	Br	65	6	91%
8	2-Octyl	OTs	65	6	93%
9	Cy	I	65	18	94%
10	EtO_2_CCH(Me)	Cl	65	1	94%

a 5 eq. of exchange resin used.

**Scheme 18:**
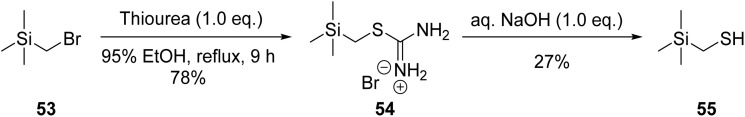
Incorporation of thiols into organosilicon substrates.

In their studies of sulfurated organosilicon compounds, Noller and Post developed a method for preparing trimethylsilylmethyl mercaptan employing thiourea as the sulfurating agent (Scheme 18).^142^ Trimethylsilyl methyl bromide was treated with equimolar thiourea and heated to reflux in absolute alcohol for nine hours, which afforded the intermediated crystalline thiouronium salt in 78% yield. Hydrolysis of this salt with sodium hydroxide solution afforded the thiol product in 27% yield.

Another early report on the conversion of mono- and dibromoalkanes to thiols *via* their isothiouronium salts was described by Cossar and colleagues.^152^ High boiling point solvents and bases allowed for easy distillation of the more volatile thiol products. Accordingly, reaction of *n*-alkyl bromides with thiourea in triethylene glycol was followed by treatment with tetraethylenepentamine to afford thiols in good yields (Table 40, entries 1–6). This approach was also applicable to dibromoalkanes, producing dithiols in yields of 58–80% (entries 7–9). The same methodology was later exploited by Otto and colleagues in the synthesis of β-sultams,^149^ while similar strategies were utilised in the development of other antibacterial^150^ and antifungal compounds.^162^

**Table 40:** Synthesis of alkane(di)thiols from alkyl halides and thiourea with basic hydrolysis

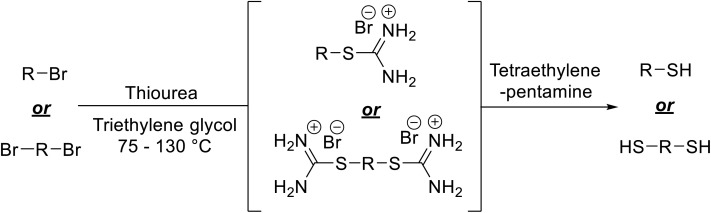				
Entry	R	Thiourea (eq.)	Base (eq.)	Yield
1	Et	1.1	1.0	68%
2	*n*-Pr	1.1	1.0	79%
3	*n*-Bu	1.1	1.0	77%
4	*n*-pentyl	1.1	1.0	75%
5	*n*-octyl	1.1	0.5	84%
6	*n*-decyl	1.1	0.5	87%
7	–(CH_2_)_3_–	2.2	1.0	58%
8	–(CH_2_)_4_–	2.2	1.0	78%
9	–(CH_2_)_5_–	2.2	1.0	80%

A mild, sodium pyrosulfite-mediated isothiouronium hydrolysis was employed by Sureshbabu *et al.* for the preparation of *N*^β^-Fmoc/Cbz-amino alkyl thiols (Table 41).^151^ This approach avoids strongly basic conditions which would normally lead to cleavage of amine protecting groups. Following initial isolation of the isothiouronium salts, treatment with a dichloromethane/water solution of sodium pyrosulfite at reflux effected complete hydrolysis after 30 minutes. Both Fmoc- (entries 1–8) and Cbz-protected (entries 9–12) substrates were compatible with these conditions, making this a suitable method for converting protected substrates.^163^ An attempted one-pot procedure, which avoids isolation of the isothiouronium intermediate, resulted in significantly lower overall yields. The utility of sodium pyrosulfite has been demonstrated separately by Pinto^164^ and Davis^165^ in their work on thiol-containing sugars.

**Table 41:** Synthesis of *N*^β^-Fmoc/Cbz-amino thiols using sodium pyrosulfite-mediated hydrolysis

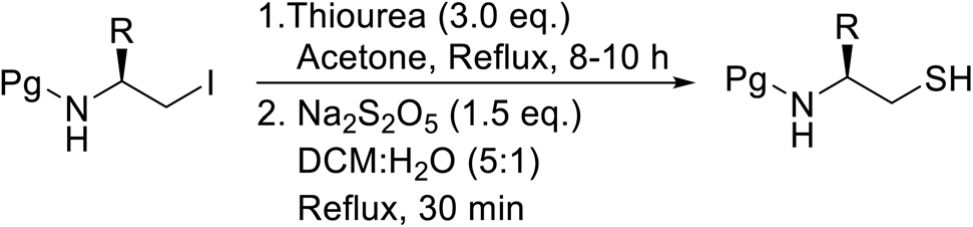				
Entry	Substrate		Yield	
	R	Pg	Isothiouronium salt	Thiol
1	H	Fmoc	92%	86%
2	Me	Fmoc	94%	92%
3	Bn	Fmoc	91%	88%
4	i-Pr	Fmoc	94%	91%
5	i-Bu	Fmoc	88%	84%
6	(CH_2_)_4_NHCbz	Fmoc	94%	88%
7	(CH_2_)_2_CO_2_Bn	Fmoc	93%	94%
8	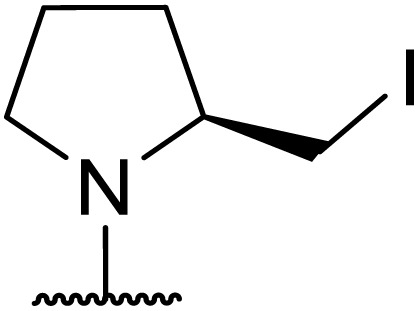	Fmoc	91%	79%
9	Bn	Cbz	92%	91%
10	*s*-Bu	Cbz	87%	88%
11	CH_2_CO_2_Bn	Cbz	93%	85%
12	Ph	Cbz	94%	90%

**Scheme 19:**
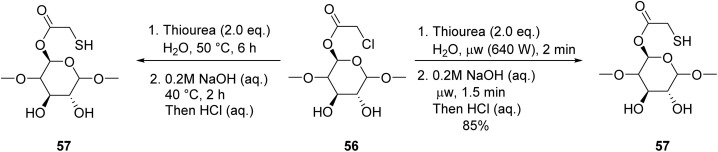
Thiolation of starch substrates.

Chauhan and colleagues successfully demonstrated the application of thiourea to the incorporation of a thiol functionality into complex substrates, such as starch.^166^ Sulfurisation of starch was observed under both standard thermal conditions and microwave irradiation (Scheme 19). Initial chloroacetylation of starch was followed by conversion to the thiouronium intermediate and, finally, hydrolysis to the thiol product.^166,167^ The authors noted that switching to microwave irradiation returned a high yield of 85%. Other successful applications of thiourea as a sulfur source include solid-phase synthesis,^168^ polymer chemistry,^169,170^ and the development of thiol-functionalised porphyrin-based nanoparticles.^171^

### 3.3 Thiocarbonic acid derivatives

For the purposes of this review, xanthates (ROCS_2_M) and trithiocarbonates (M_2_CS_3_) are both categorised as thiocarbonic acid derivatives. These reagents are generally less prevalent in the literature than thioacetates and thiourea, but react similarly with alkyl halides to afford stable intermediates which are converted to the target thiols using a variety of methods. Additionally, these reagents are compatible with sensitive substrates,^163^ and may even be employed as chiral auxiliaries where control of stereochemistry is important.^172^ Akin to thioacetates and thioureas, most literature precedent confirms their ability to effect the halide to thiol transformation for primary aliphatic substrates. Literature examples also exist for aliphatic halides with higher degrees of substitution.^172–174^

Xanthates constitute a useful sulfur source for the preparation of thiols. An early method was described by Djerassi *et al.* who treated aliphatic bromides with potassium ethyl xanthate or sodium benzyl xanthate (Table 42).^173^ In most cases, the xanthate was initially prepared and subsequently reduced with lithium aluminium hydride, although the thiol could be obtained directly without isolation of the salt (entries 2, 5, 8, 10, 12). This also proved to be a convenient route to β-mercaptoalcohol products from α-halocarbonyls (entries 4–9, 11, 12).

**Table 42:** Synthesis of aliphatic thiols from aliphatic halides and xanthates

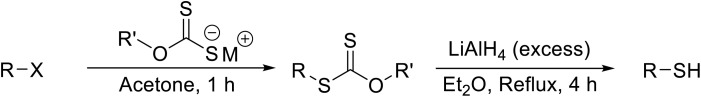					
Entry	Substrate	Xanthate		Thiol	
	R–X	R′	Yield	R	Yield
1	BnBr	Et	70%	Bn	>80%
2	BnBr	Bn	n/a^a^	Bn	>80%
3	BnCH_2_Br	Et	83%	BnCH_2_	88%
4	PhCOCH_2_Br	Bn	75%	PhCH(OH)CH_2_	85%
5	PhCOCH_2_Br	Et	n/a^a^	PhCH(OH)CH_2_	87%
6	PhCOCH(Ph)Br	Bn	60%	PhCH(OH)CH(Ph)	34%
7	BnCOCH_2_Br	Bn	66%	BnCH(OH)CH_2_	n/a^a^
8	BnCOCH_2_Br	Et	n/a^a^	BnCH(OH)CH_2_	75%
9	HO_2_CCH(Ph)Cl	Et	71%	HOCH_2_CH(Ph)	70%
10	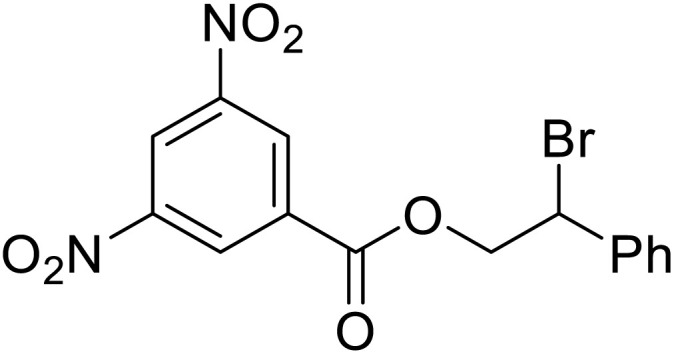	Et	n/a^a^	HOCH_2_CH(Ph)	39%
11	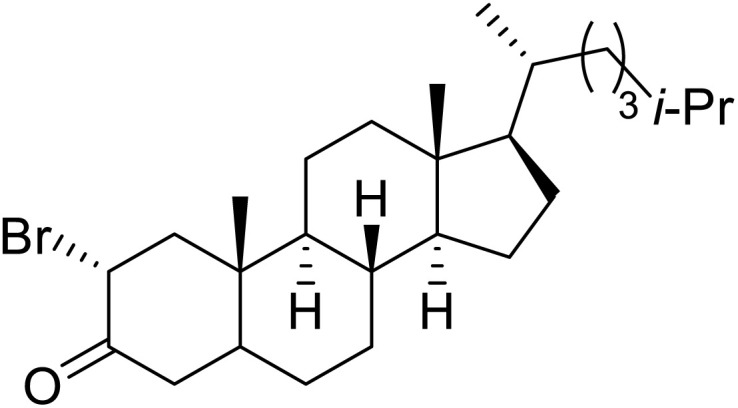	Et	73%	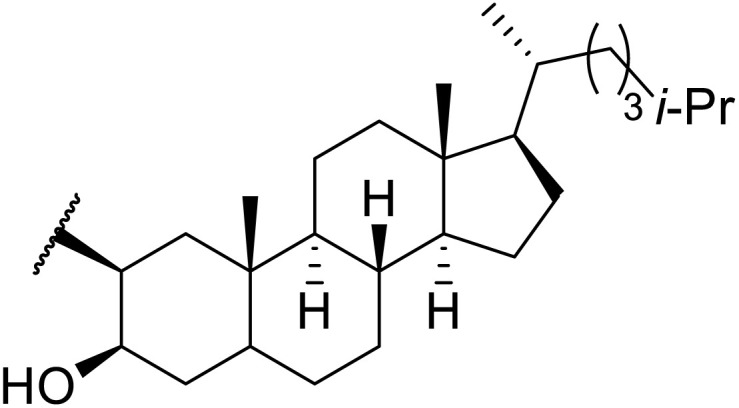	n/a^a^
12	(Ph)_2_CHCOCH_2_Br	Bn	n/a^a^	(Ph)_2_CHCH(OH)CH_2_	71%

a Not isolated.

Isola and co-workers have shown how xanthates can provide access to chiral thiols.^172^ (−)-Sodium *O*-menthyl xanthate, prepared from (−)-menthol,^175^ acts as a chiral sulfur transfer agent (Table 43). Reaction of racemic benzhydryl halides with the (−)-sodium *O*-menthyl xanthate afforded a diastereomeric mixture of xanthate esters. Subsequent fractional crystallisation afforded a diastereomerically pure xanthate ester which was morpholinolysed to the optically pure thiol. Although yields are modest, this represents one of the few preparative methods which avoids racemisation of the chiral centre. Xanthates have been similarly employed in the synthesis of chiral amino acids,^176^ as well as chiral muscarinic receptor agonists.^177^

**Table 43:** Synthesis of optically active thiols using a chiral sulfur transfer reagent

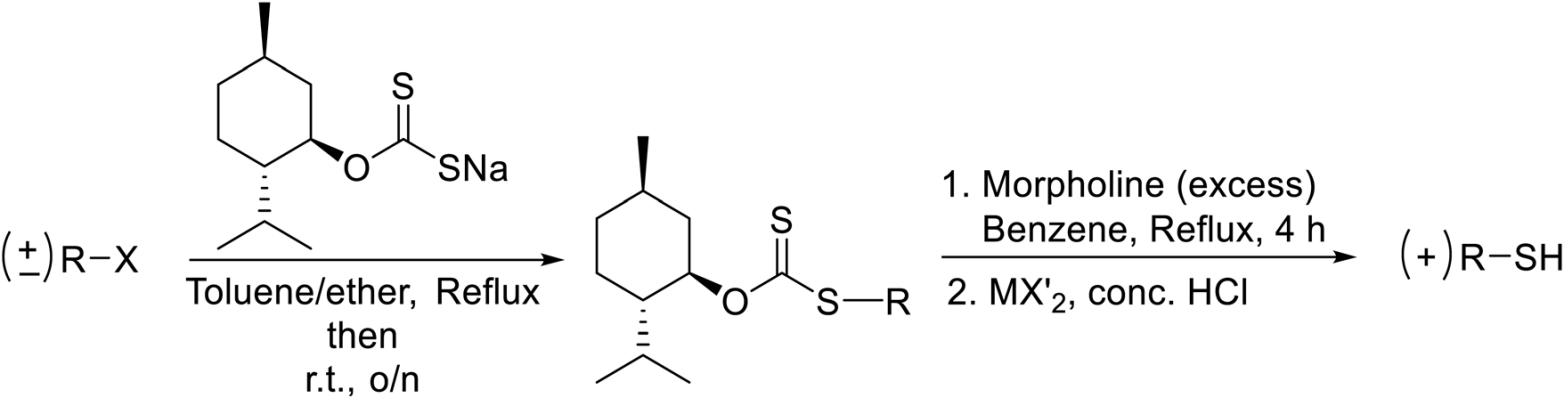							
Entry	R	X	Time (h)	Yield (xanthate)	Morpholine (eq.)	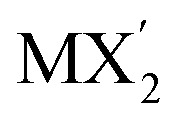	Yield ((+)-thiol)
1	PhCH(Me)	Br	4	31%	7.7	HgCl_2_	48%
2	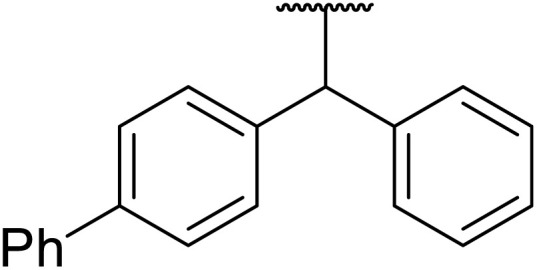	Cl	4	27%	14.6	Pb(OAc)_2_	51%
3	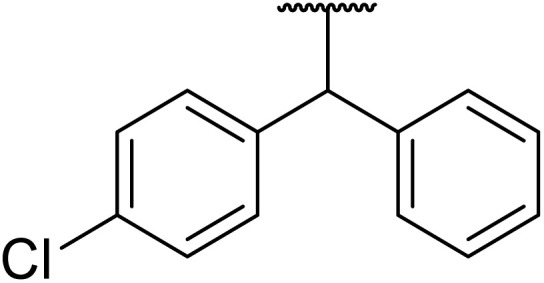	Cl	4	14%	2.2	HgCl_2_	47%
4^a^	2-Octyl	Br	12	37%			
5^a^	2-Bu	Br	12	38%			
6^a^	PhCH_2_CH(Me)	Br	12	38%			

a Not converted to thiol.

Trithiocarbonates, also known as thioxanthates, represent a similar class of sulfur transfer reagent. Martin and Greco developed a one-step preparation of dithiols and thiols using sodium trithiocarbonate,^178^ which can be readily prepared by the reaction of sodium sulfide with carbon disulfide (Table 44).^179^ Addition of sodium trithiocarbonate to various alkyl dichlorides generated the corresponding bis(trithiocarbonic) acids upon acidification, which spontaneously decomposed to dithiols (entries 1–3). Monothiols could also be accessed in this manner, albeit in low yields (entry 4) – no explanation for this outcome is provided.

**Table 44:** Sodium trithiocarbonate as a sulfur transfer reagent

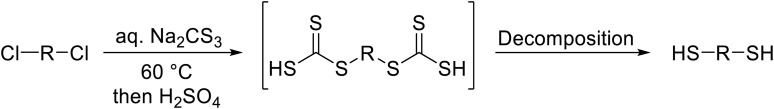					
Entry	Halide	Na_2_CS_3_ (eq.)	Time (min)	Product	Yield
1	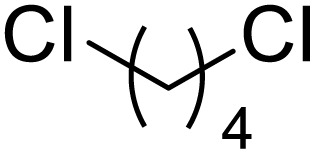	3.0	325	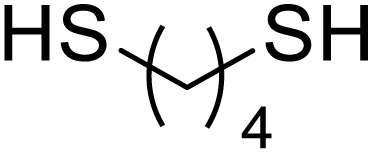	61%
2	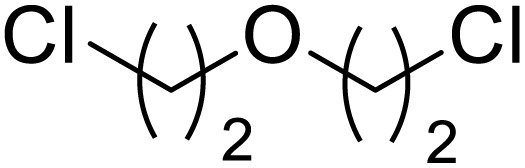	3.0	150	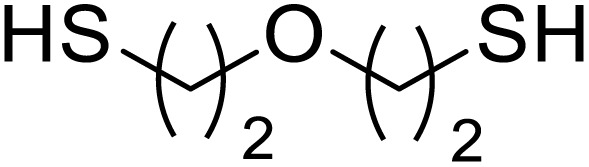	66%
3	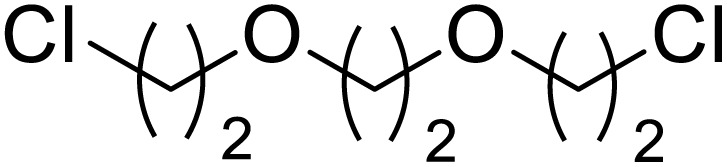	2.9	315	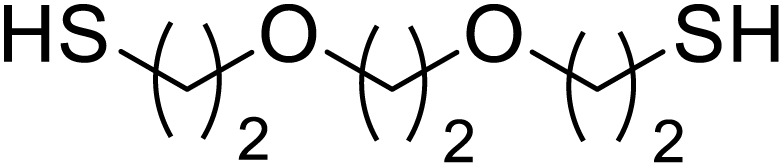	77%
4	BnCl	1.5	81	BnSH	25%

A mild methodology for the preparation of chiral β-amino alkyl thiols was reported by Sureshbabu and co-workers.^163^ Various amino alkyl iodides were initially treated with sodium trithiocarbonate in dioxane at 50 °C and then acidified to furnish the thiol (Table 45). Cbz- (entries 1–5) and Boc-protected (entries 6–9) substrates were transformed in good yields. By contrast, Fmoc-protected amino alkyl thiols were prone to cleavage of the protecting group, resulting in lower yields (entries 10–11).

**Table 45:** Synthesis of *N*-protected chiral β-amino alkyl thiols using sodium trithiocarbonate

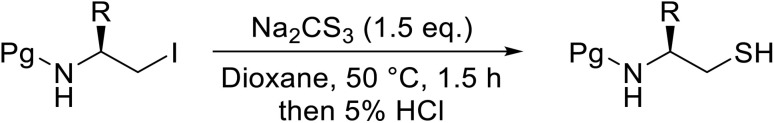			
Entry	R	Pg	Yield
1	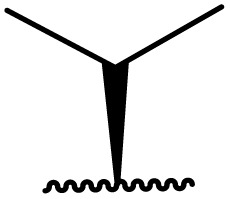	Cbz	85%
2	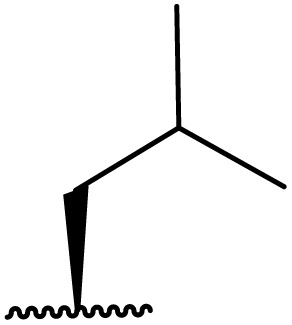	Cbz	86%
3	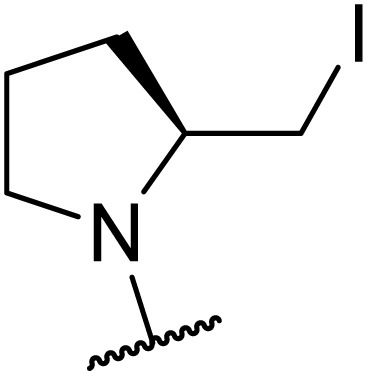	Cbz	78%
4	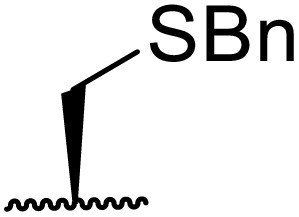	Cbz	75%
5	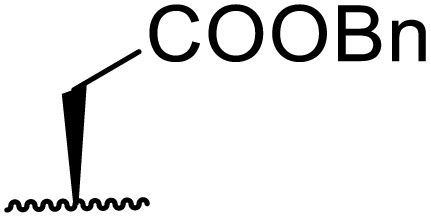	Cbz	62%
6	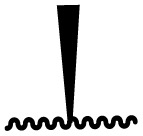	Boc	76%
7	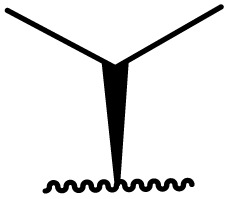	Boc	67%
8	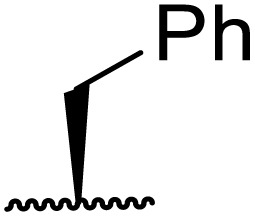	Boc	70%
9	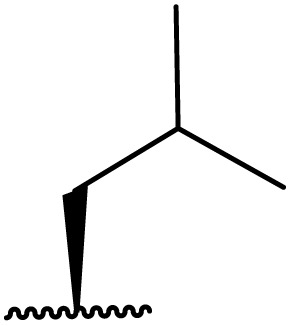	Boc	65%
10	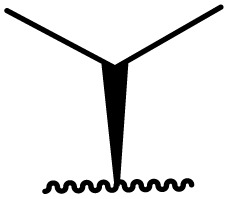	Fmoc	48%
11	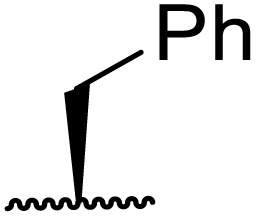	Fmoc	55%

**Scheme 20:**
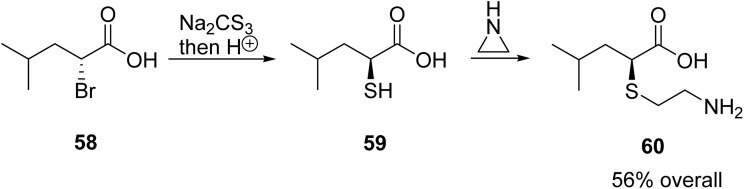
Application of sodium trithiocarbonate in the synthesis of chiral thioethers.

Yankeelov and colleagues also exploited this methodology for the synthesis of chiral thioether-linked dipeptide analogues.^174^ Nucleophilic substitution of (*R*)-α-bromocarboxylic acid 58 with sodium trithiocarbonate proceeded with complete inversion to afford (*S*)-α-thiocarboxylic acid 59 following acidification (Scheme 20). Addition of ethylene imine produced thioether 60 in 56% overall yield from 58.

### 3.4 Heteroaryl thiones

Heteroaryl thiones belong to a niche class of sulfur transfer reagent.^180,181^ Treatment of the halide with the heteroaryl thione typically results in the formation of an *S*-alkylated salt which can be isolated as a crystalline, odourless and “protected” version of the desired thiol, or can be readily hydrolysed to the synthetic target. Modification of the heteroaryl thione structure can also influence the reaction mechanism, as in the case of the work described by Katritzky, where the intermediate salt undergoes an intramolecular displacement reaction, thus obviating a specific hydrolysis step.^182^ These reactions most commonly proceed in high yields and are compatible with α-halocarbonyl substrates,^183^ unlike thiourea which results in thiazole formation.^184^ The halide to thiol transformation normally occurs at mild to moderate temperatures, although elevated temperatures have been used in some cases *e.g.* solvent-free preparations of the intermediate salt.^185^

Takamoto pioneered the use of pyridothiones, specifically *N*-methyl-2(1*H*)-pyridothione, as sulfur transfer reagents (Table 46).^183^ Primary (entries 1–2) and secondary (entry 3) alkyl bromides, in addition to α-chlorocarbonyls (entries 4–5), proved suitable substrates. Hydrolysis of the thiopyridinium salts was conducted *in situ* with sodium hydroxide over 30 minutes. Albright subsequently exploited this reagent for the preparation of novel angiotensin-converting enzyme inhibitors for hypertension management.^180^

**Table 46:** Preparation of alkyl thiols using *N*-methyl pyridine-2-thione

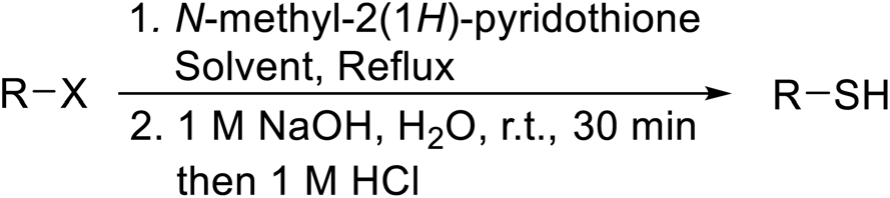						
Entry	Substrate		Solvent	Time (h)	Yield	
	R	X			Salt	Thiol
1	Bn	Br	MeCN	0.5	83%	90%
2	BnCH_2_	Br	EtOH	4	84%	82%
3	Cy	Br	*n*-PrOH	16	n/a^a^	70%
4	PhCOCH_2_	Cl	EtOH	4	81%	72%
5	EtO_2_CCH_2_	Cl	EtOH	4	83%	65%
6	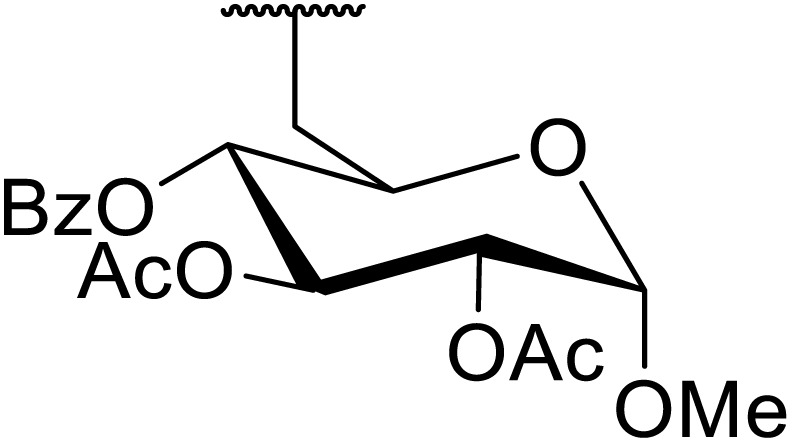	Br	*n*-PrOH	8	n/a^a^	71%

a Too hygroscopic to isolate.

**Scheme 21:**
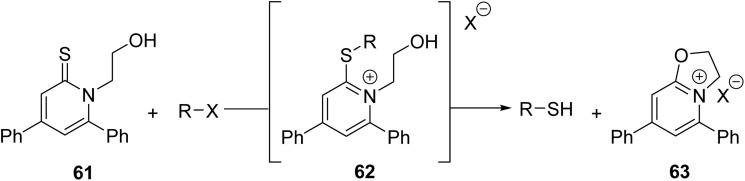
1-(2-Hydroxyethyl)-4,6-diphenylpyridine-2-thione as a thiolating agent.

Katritzky and co-workers developed 1-(2-hydroxyethyl)-4,6-diphenylpyridine-2-thione (61) for the one-pot conversion of *n*-alkyl halides under neutral conditions (Scheme 21).^182^ The key feature of this reagent is the rapid intramolecular displacement step which liberates the thiol product. Reaction of 4,6-diphenyl-2-pyrone with phosphorus pentasulfide generates 4,6-diphenylpyran-2-thione which is transformed into 61 on addition of ethanolamine.

In benzene at 20 °C, benzylic iodides (Table 47, entry 1) and bromides (entries 2–5) reacted with 61 to furnish thiols in good yields. While alkyl iodides (entry 6) were compatible substrates, alkyl bromides and chlorides were inert under these conditions. However, switching to acetonitrile at room temperature, with tetraethylammonium iodide as a halide exchange agent (Method B), was met with success (entries 8–12). Both methods were also selective for aliphatic over aromatic halides (entries 1, 4, 7, 8).

**Table 47:** Synthesis of thiols from alkyl halides using pyridine-2-thione 61

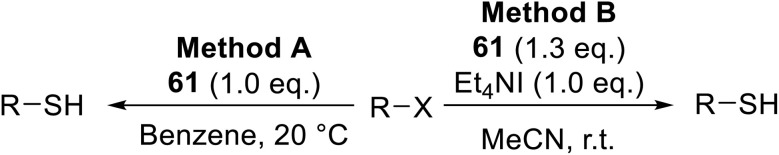					
Entry	Substrate		Time (h)	Method	Yield
	R	X			
1	4-ClC_6_H_4_CH_2_	I	3	A	89%
2	4-MeC_6_H_4_CH_2_	Br	10	A	76%
3	Bn	Br	20	A	80%
4	4-BrC_6_H_4_CH_2_	Br	20	A	74%
5	4-O_2_NC_6_H_4_CH_2_	Br	20	A	67%
6	*n*-Hexyl	I	24	A	69%
7	4-BrC_6_H_4_COCH_2_	Br	10	A	a72%
8	4-ClC_6_H_4_CH_2_	Cl	24	B	72%
9	*n*-Bu	Cl	28	B	65%
10	*n*-Pentyl	Cl	26	B	61%
11	*n*-Hexyl	Br	24	B	66%
12	*n*-Heptyl	Br	26	B	65%

a Product isolated as the disulfide.

**Scheme 22:**
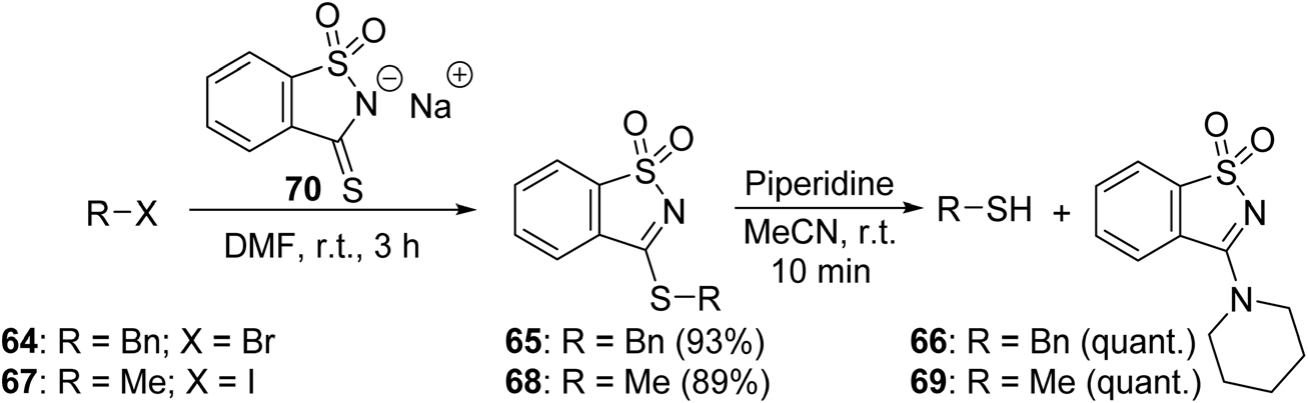
Sodium thiosaccharin as a thiolating agent.

In their search for novel herbicidal and fungicidal agents, Kotake *et al.* utilised 3-substituted 1,2-benzisothiazole 1,1-dioxides 65 and 68 as a safer alternative to thiols (Scheme 22).^181^ Benzyl bromide (64) and methyl iodide (67) reacted with sodium thiosaccharin (70) to generate the corresponding crystalline and odourless surrogates. Subsequent treatment with piperidine produced the target thiols in quantitative yields after 10 minutes.

Andreoli investigated 3-(2-aminophenyl)-4-methyl-1,3-thiazole-2(3*H*)-thione (71) as a means of converting alkyl iodides and alkyl diiodides into thiols and dithiols respectively under mild conditions (Table 48).^185^ Neat reaction of 71 with excess alkyl iodide produces the thiazolium salts almost quantitatively (entries 1–5). Subsequent reflux in methanol affords thiols in excellent overall yields. Gratifyingly, no disulfide formation was observed. The thiazolium salts are stable and odourless compounds which can be stored indefinitely at 3–4 °C. Similarly, alkyl diiodides were readily converted to bisthiazolium salts using two equivalents of thione (entries 6–8). The corresponding dithiols were subsequently obtained in excellent overall yields *via* refluxing methanolysis.

**Table 48:** Conversion of alkyl iodides to thiols under neutral conditions

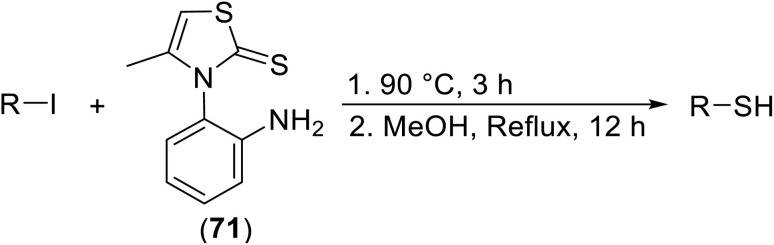				
Entry	R–I	R–I (eq.)	Salt yield	Thiol yield
1	CH_3_(CH_2_)_6_I	10.8	96%	90%
2	CH_3_(CH_2_)_8_I	10.5	96%	92%
3	CH_3_(CH_2_)_9_I	9.7	94%	94%
4	CH_3_(CH_2_)_11_I	8.4	93%	91%
5	CH_3_(CH_2_)_17_I	7.4	98%	92%
6^a^	I(CH_2_)_3_I	0.5	94%	91%
7^a^	I(CH_2_)_4_I	0.5	95%	90%
8^b^	I(CH_2_)_5_I	0.5	89%	92%

a Heated to reflux in CHCl_3_ for 24 h.

b Heated to reflux in CHCl_3_ for 48 h.

### 3.5 Silylated sulfur sources

Organosilane thiolates and sulfides represent another important class of sulfurating agents. Upon reaction of the halide substrate with the organosilane thiolate/sulfide, an intermediate organosilane thioether is generated. Similar to other methods described in this review, this silyl ether acts as a “protected form” of the desired thiol. The intermediate is normally converted to the thiol *via* fluoride-mediated desilylation, providing good compatibility with sensitive functional groups.^186^ Several studies have highlighted the utility of these reagents in converting vinyl (and aryl) halides to thiols in high yields.^187,188^ Vinyl halide substrates are rarely encountered in other methodologies.

Soderquist and colleagues developed an effective thiolating reagent prepared from lithium sulfanide and triisopropylsilyl chloride (TIPSCl) in 98% yield.^187^ The potassium salt of triisopropylsilanethiol (KSTIPS) was found to be stable and crystalline, and particularly effective in subsequent thiolations (Table 49).^188^ Reaction of KSTIPS with an excess of the alkyl or benzyl halide afforded a protected form of the corresponding (di)thiol in good to excellent yield. Control of KSTIPS stoichiometry selectively delivered the mono- or dithiolated product (entries 8 and 9). Additionally, the reagent displayed a preference for thiolation of primary alkyl halides over secondary alkyl halides (entry 10). Dithiolation was more prevalent with DMF as sovlent (entry 11).

**Table 49:** Application of KSTIPS as a thiolating agent

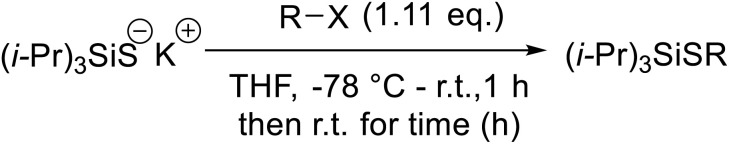				
Entry	R	X	Time (h)	Yield ((i-Pr)_3_SiSR)
1	Et	Br	6	91%
2	Et	I	5	85%
3^a^	*n*-Bu	Br	3	90%
4	*n*-Octyl	Br	6	91%
5	Bn	Cl	1	83%
6	Bn	Br	0.25	88%
7	CH_2_=CHCH_2_	Br	1	86%
8^b^	Br–(CH_2_)_2_–	Br	8	73%
9^c^	–(CH_2_)_2_–	Br	4	82%
10^d^	MeCHBr(CH_2_)_2_–	Br	3	92%
11^e^	–CHMe(CH_2_)_2_–	Br	3	95%

a *n*-BuBr added at 25 °C.

b 2.0 eq. of 1,2-dibromoethane used and monosulfurated product obtained.

c 2.4 eq. of (i-Pr)_3_SiSK used and disulfurated product obtained.

d Monosulfurated product obtained.

e DMF used as solvent and disulfurated product obtained.

**Scheme 23:**
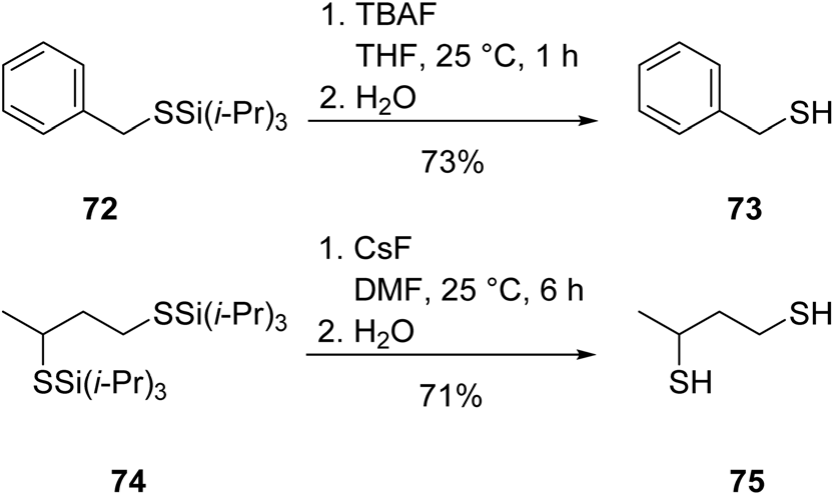
Desilylation of silyl thioethers.

These silica-stable silyl ether intermediates could be readily desilylated with CsF or TBAF, with examples from entries 5 and 11 illustrated in Scheme 23.

Given its hydrolytic stability and resistance to organometallic-induced degradation, the same group later investigated KSTIPS in palladium-catalysed cross-coupling reactions.^187,188^ Pd(PPh_3_)_4_-mediated coupling of vinyl and aryl halides gave good to excellent yields of the vinyl-STIPS or aryl-STIPS products (Table 50). Retention of stereochemistry was observed with respect to vinyl substrates (entries 2 and 3). Electron-withdrawing substituents on aryl substrates were found to increase the coupling rate (entry 5 *vs.* 7 *vs.* 8). The authors reported that the silyl ethers could be desilylated using a combination of caesium fluoride in DMF.

**Table 50:** Palladium-catalysed cross-coupling of aryl/vinyl halides with KSTIPS

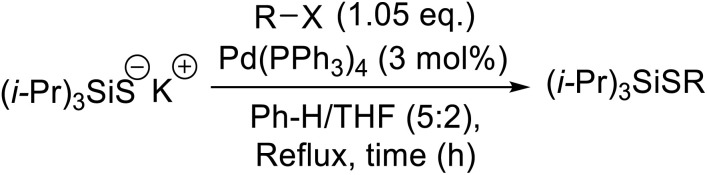				
Entry	R	X	Time (h)	Yield (RSTIPS)
1	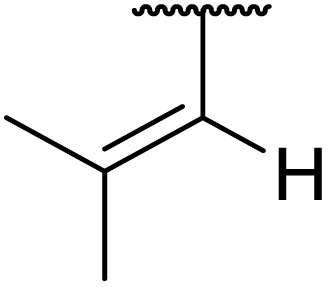	Br	36	84
2	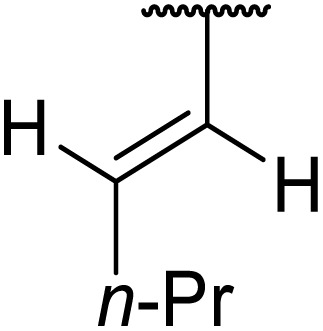	I	2	66
3	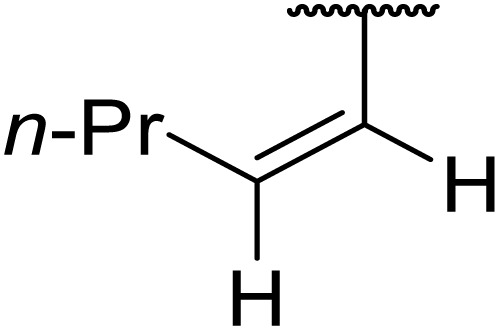	I	2	89
4	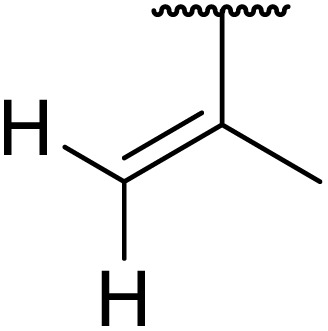	Br	1.5	92
5	Ph	Br	2	77
6	1-Naphthyl	Br	5	61
7^a^	4-O_2_NC_6_H_4_	Br	0.25	93
8^a^	4-MeOC_6_H_4_	Br	12	68
9^a^	2-Pyridyl	Br	12	79

a Aryl halide heated to reflux for one hour with catalyst prior to KSTIPS addition.

Hu and Fox investigated tetrabutylammonium trimethylsilylthiolate as a neutral thiolating agent which liberates thiols during aqueous workup or filtration through silica gel.^189^ The reagent is prepared by reaction of hexamethyldisilathiane with TBAF. TBAF appears to increase the reagent nucleophilicity, facilitating low temperature reactions. Furthermore, by eschewing basic hydrolysis conditions, the formation of disulfide and sulfonic acid side products is greatly reduced. Accordingly, transformation of longer chain alkyl bromides (Table 51, entries 1–4) and chlorides (entry 5) proceeded in high yields. This reagent avoids unwanted intramolecular single-electron transfer oxidations often observed during the synthesis of highly electron-deficient thiols. Sensitive thiols, which are prone to decomposition under basic conditions, were recovered in good yields (entries 7–9).

**Table 51:** Synthesis of thiols using tetrabutylammonium trimethylsilylthiolate under mild conditions

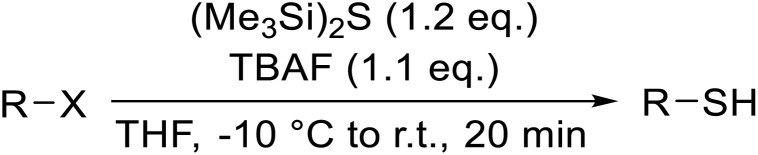			
Entry	R	X	Yield
1	*n*-Decyl	Br	a94%
2	HO(CH_2_)_6_	Br	a87%
3	Br(CH_2_)_4_	Br	a77%
4	Cl(CH_2_)_4_	Br	a82%
5	HO(CH_2_)_4_	Cl	a79%
6	Bn	Cl	a90%
7	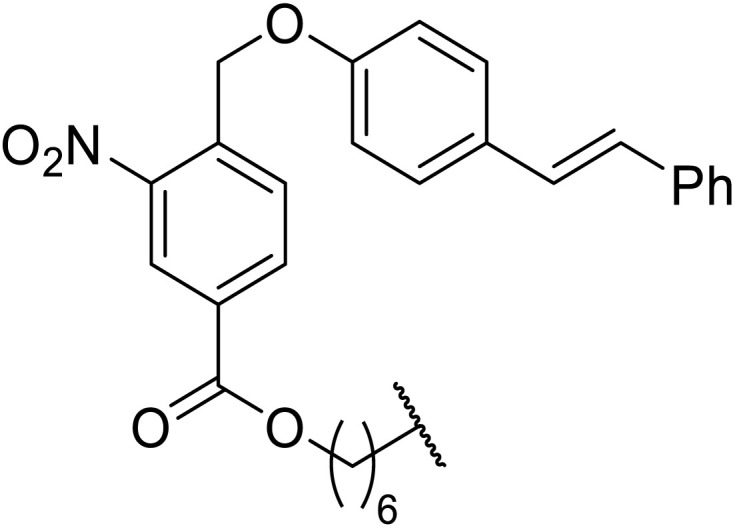	Br	b74%
8	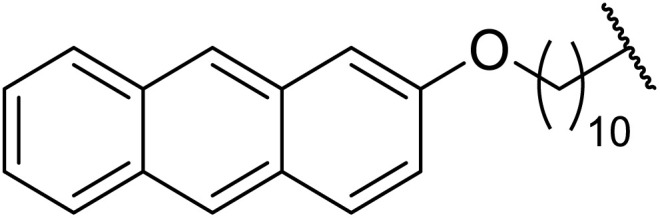	Br	b80%
9	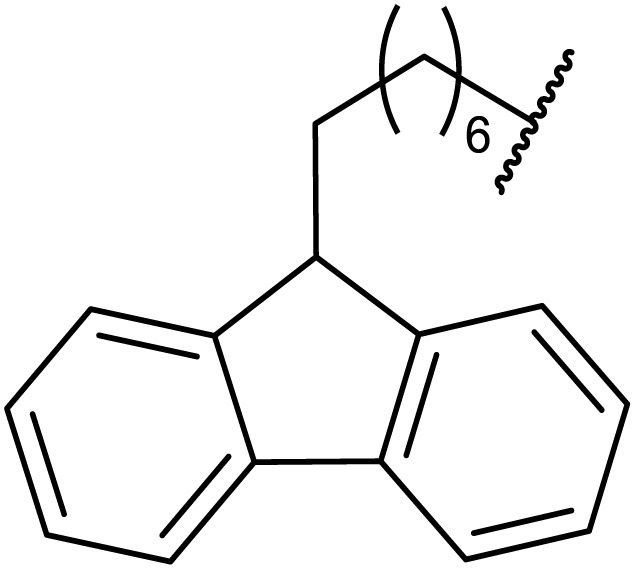	Br	b68%

a Yield obtained by gas chromatography.

b Isolated yield.

Harpp and Kobayashi successfully prepared several aliphatic and aryl thiols from α-trimethylsiloxyalkanethiols and bromide precursors (Table 52).^190^ α-Trimethylsiloxyalkanethiols were generated by the reaction of an appropriate aldehyde with hydrogen sulfide and trimethylsilyl chloride in pyridine.^191^ Treatment of the α-trimethylsiloxyalkanethiol with *n*-butyllithium, followed by alkylation with the relevant alkyl bromide, afforded the sulfide, desilylation of which produced thiols in high yields (entries 1–5).

**Table 52:** Thiol preparation using α-trimethylsiloxy alkanethiol synthons

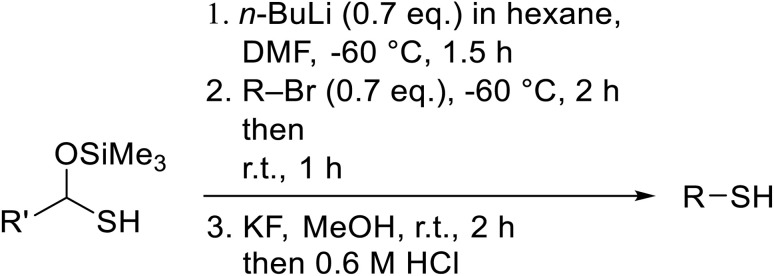			
Entry	R	R′	Yield
1	Ph	*n*-Pr	a84%
2	Bn	*n*-Pr	a86%
3	Bn	Et	a95%
4	Ph	i-Pr	77%
5	*n*-hexyl	*n*-Pr	69%

a Yield determined by GLC analysis.

A summary of the key features of the different methodologies for converting aliphatic halides to thiols is outlined in Table 53.

**Table 53:** Summary of approaches for the preparation of aliphatic thiols from aliphatic halides

Approach	Characteristics	Advantages	Disadvantages
Inorganic sulfur sources	Wide variety of reagents	Suitable for RCl, RBr and multihalogenated substrates	Often toxic/hazardous reagents
Thioacetates and thiourea	Intermediate hydrolysis step required	Good functional group compatibility	Limited examples of 2°/3° RX
Thiocarbonic acid derivatives	Conversion of intermediate required	High degree of stereocontrol	Limited examples of 2°/3° RX
Heteroaryl thiones	Isolatable intermediate formed	Intermediate can form thiol spontaneously; α-halocarbonyl compatibility	Elevated temperature required for solvent-free thiol preparation
Silylated sulfur sources	Fluoride-mediated desilylation of intermediate	Vinyl halide-compatible	Sub-0 °C temperatures often required

## 4. Aromatic thiols

This section describes a variety of methods for the preparation of aromatic thiols from aryl halides. The majority of routes reported to date employ metal catalysis. Although photochemical^192^ and electrochemical^193^ approaches have been explored, there are far fewer successful examples of these in the literature. Accordingly, this section has been mainly divided by the metal employed rather than the sulfur source as in the previous section. The different methodologies include: 4.1 Copper catalysis, 4.2 Palladium catalysis, 4.3 Nickel catalysis, and 4.4 Miscellaneous methods.

### 4.1 Copper catalysis

Copper catalysis has gained significant traction in recent years due to its applicability to a diverse range of chemical transformations, as well as the cost-effectiveness and sustainability of copper compared to other precious metals.^194,195^ Additionally, copper salts are generally tolerant of non-inert conditions and can be used in oxidative environments and protic solvents.^196^ Typical loadings fall in the range of 5–10 mol%, although this can be further reduced when copper nanoparticles are employed.^197^ Complex and expensive ligands are generally not required, with copper powder sometimes directly employed.^198^ In certain cases, elemental sulfur can act as the sulfur source to afford the target aryl thiols in excellent yields.^197,199^ Thiolations using copper are generally limited to aryl iodides, however, and transformations of other aryl halides are much rarer.

The coupling of *S*-thiobenzoic acid with aryl halides under copper catalysis to afford *S*-aryl thiobenzoates has been reported by Sawada and co-workers.^200^ 10 mol% copper iodide, with 1,10-phenanthroline as the ligand and Hünig's base in toluene, saw excellent conversions of substituted aryl iodides at 110 °C (Table 54, entries 1–8). Equally high yields were recorded for electron-poor (entries 2, 3), electron-rich (entries 5–7) and sterically hindered (entry 7) substrates. This chemistry was also compatible with heteroaryl iodides (entry 8) but failed in the case of aryl bromides (entry 9). The *S*-aryl thiobenzoate intermediates can be readily converted to the corresponding thiols by basic hydrolysis in quantitative yields.

**Table 54:** Synthesis of *S*-aryl thiobenzoates under CuI catalysis

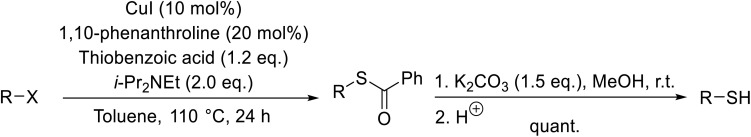			
Entry	Substrate		Yield (thioester)
	R	X	
1	4-AcC_6_H_4_	I	97%
2	3-O_2_NC_6_H_4_	I	99%
3	4-BrC_6_H_4_	I	94%
4	1-Naphthyl	I	97%
5	4-MeOC_6_H_4_	I	99%
6	4-Toluyl	I	100%
7	2-Toluyl	I	99%
8	3-Pyridyl	I	99%
9	Ph	Br	Trace

Inspired by previous findings by Taniguchi,^201^ Jiang *et al.* demonstrated the direct coupling of elemental sulfur with aryl iodides under copper catalysis to afford diaryl disulfides.^199^ By incorporating an *in situ* reduction of the disulfide, they ultimately created a one-pot methodology for producing aryl thiols (Table 55). The disulfide intermediates could be reduced using either sodium borohydride (Method A) or triphenylphosphine and hydrochloric acid (Method B). Substrates containing electron-donating (entries 1–6) and electron-withdrawing (entries 12–18) groups were well tolerated. Highly electron-deficient substrates reacted in the absence of a catalyst (entries 19–21) *via* nucleophilic aromatic substitution. However, even in these selected cases, the presence of a catalyst was associated with higher yields (entry 21 *vs.* 22). The authors suggest that polysulfide ions are the likely active species in these reactions.

**Table 55:** One-pot, copper-catalysed coupling/reduction methodology

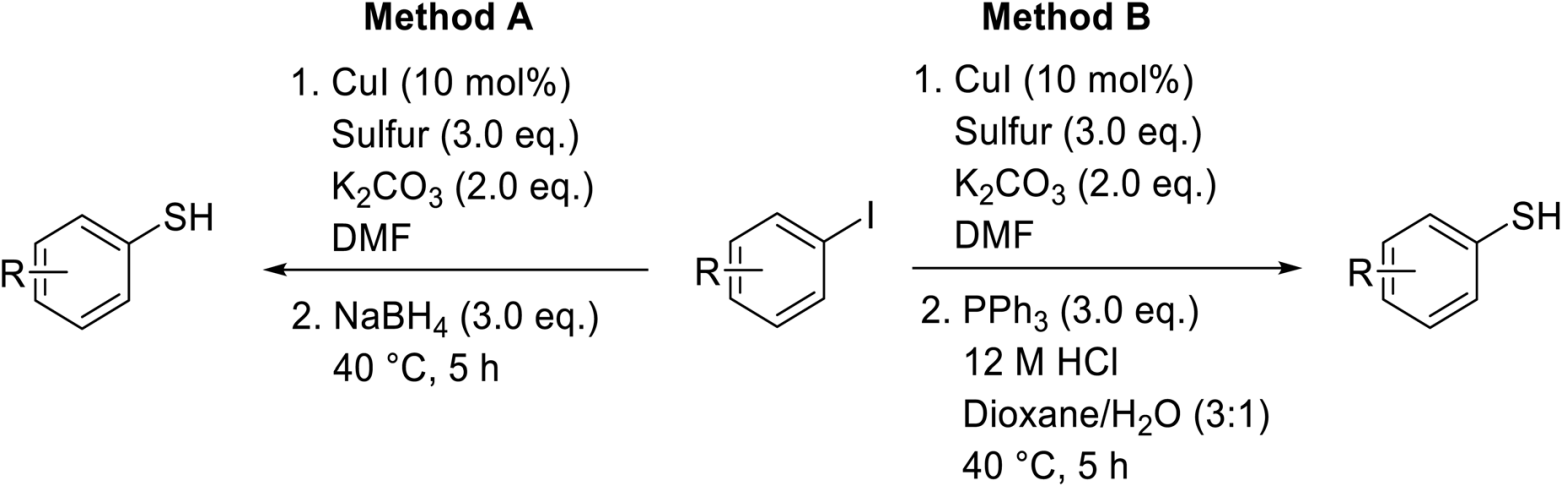					
Entry	R	Temp. (°C)	Time (h)	Method	Yield
1	2-MeO	90	12	A	90%
2	3-MeO	90	12	A	89%
3	2-Me	90	12	A	90%
4	3-Me	90	12	A	92%
5	4-Me	90	12	A	90%
6	3,5-Me	90	12	A	93%
7	4-Ph	90	12	A	84%
8	2-HO_2_CCH_2_	90	12	A	82%
9	4-AcHN	90	12	A	90%
10	4-HOCH_2_	90	12	A	89%
11	4-HO(Me)CH	90	12	A	88%
12	4-Br	90	12	A	90%
13	3-F	90	8	A	92%
14	4-F	90	8	A	88%
15	2-HO_2_C	90	12	A	88%
16	3-HO_2_C	90	12	A	91%
17	4-HO_2_C	90	16	A	87%
18	3-F_3_C	90	8	A	90%
19	4-Ac	60	12	B	a84%
20	4-O_2_N	60	4	B	a73%
21	3-O_2_N	90	12	B	a53%
22	3-O_2_N	90	5	B	84%
23	4-HO_2_C	90	12	A	a5%

a No addition of CuI.

Qi *et al.* utilised thiourea as a sulfur source to prepare aryl thiols from aryl iodides (Table 56).^202^ Optimisation of the reaction conditions led to the conclusion that copper iodide, l-proline, sodium *tert*-butoxide and DMSO were the most effective catalyst, ligand, base and solvent combination respectively. Hydrolysis of the isothiouronium iodide salt generates the desired thiol. *para*-Substituted aryl iodides (entry 3) reacted faster than their *ortho*-substituted counterparts (entry 1). Electron-deficient (entries 6–13), electron-rich (entries 14–19) and heteroaryl (entry 20) iodides were converted in high yields.

**Table 56:** Thiourea as a sulfur source for coupling with aryl iodides

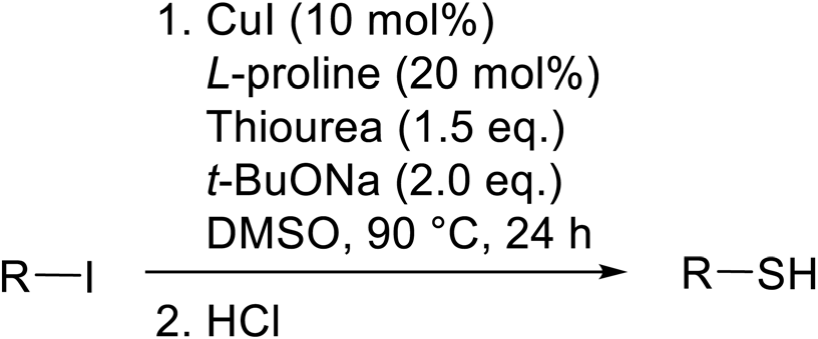		
Entry	R	Yield
1	2-Toluyl	86%
2	3-Toluyl	90%
3	4-Toluyl	90%
4	2,5-Me_2_C_6_H_3_	82%
5	2,6-Me_2_C_6_H_3_	83%
6	4-FC_6_H_4_	97%
7	4-ClC_6_H_4_	93%
8	4-BrC_6_H_4_	90%
9	4-O_2_NC_6_H_4_	95%
10	4-F_3_CC_6_H_4_	96%
11	4-NCC_6_H_4_	90%
12	4-HO_2_CC_6_H_4_	90%
13	4-AcC_6_H_4_	91%
14	4-MeOC_6_H_4_	86%
15	3-MeOC_6_H_4_	85%
16	4-H_2_NC_6_H_4_	81%
17	3-H_2_NC_6_H_4_	82%
18	4-PhC_6_H_4_	83%
19	1-Naphthyl	80%
20	3-Pyridyl	90%

**Scheme 24:**
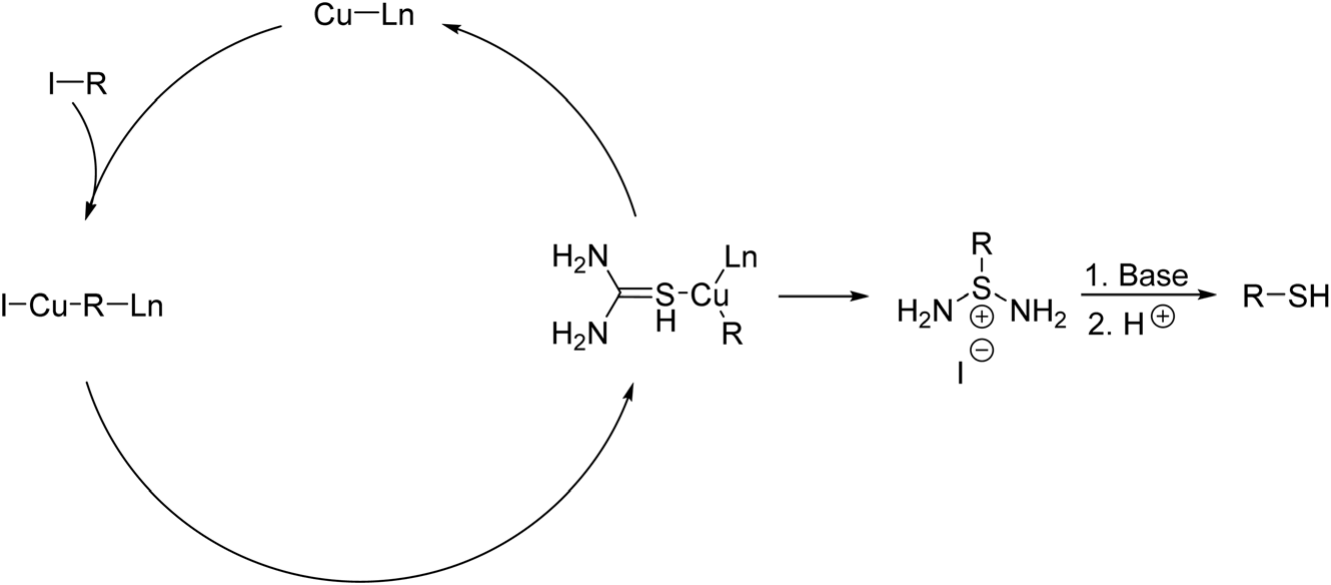
Mechanism for copper-mediated coupling of thiourea.

The mechanism proceeds *via* initial oxidative addition of the aryl iodide to give an activated aryl-copper complex which can then undergo reaction with thiourea (Scheme 24). Subsequent reductive elimination affords the C–S cross-coupling product and regenerates the catalyst. Hydrolysis of the *S*-isothiouronium intermediate with base, followed by acidification with hydrochloric acid, affords the aryl thiol.

Xu *et al.* employed copper iodide nanoparticles (NPs) which permits the use of ligand-free conditions for direct coupling with elemental sulfur.^197^ These nanoparticles exhibit “semi-heterogenous catalysis”, combining the recovery and recyclability of heterogenous catalysts with the good selectivity and low loadings typical of homogenous catalysts. This methodology employed a two-stage process, namely initial coupling with sulfur to form the disulfide, followed by zinc-mediated reduction to the thiol (Table 57). Conversion of electron-poor (entry 2), electron-rich (entries 3–6), and sterically hindered (entry 7) aryl iodides proceeded in high yields. Although coupling of aryl bromides with electron-withdrawing substituents (entries 8, 9) was observed, unactivated aryl bromides remained mostly unreacted (entries 10, 11). In competition studies, iodobenzene was selectively converted into thiophenol in the presence of aqueous ammonia and sulfur, confirming that *S*-arylation is favoured.

**Table 57:** Ligand-free coupling of aryl halides using CuI nanoparticles

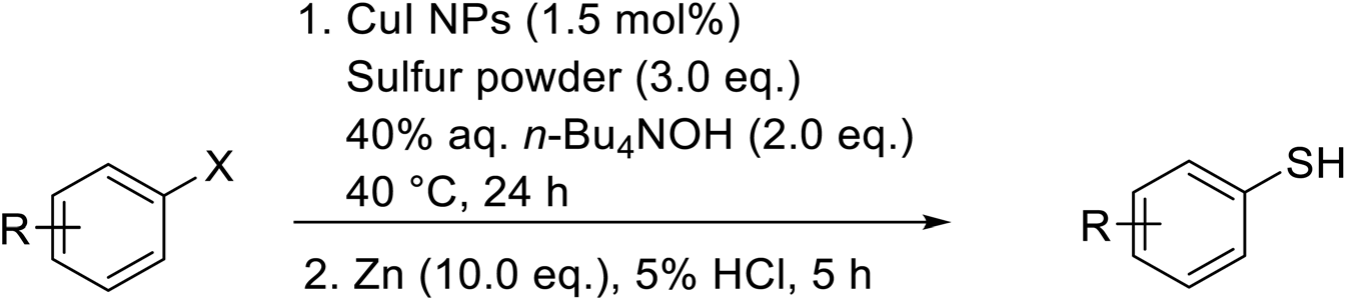			
Entry	Substrate		Yield
	R	X	
1	H	I	94% (a77%)
2	4-Cl	I	94%
3	4-Me	I	92%
4	4-MeO	I	91%
5	4-H_2_N	I	91%
6	2-H_2_N	I	89%
7	2,6-Me	I	86%
8	4-HO_2_C	Br	b87%
9	2-HO_2_C	Br	b84%
10	H	Br	trace^b^
11	4-Me	Br	trace^b^

a At 25 °C.

b 3.0 mol% CuI NPs at 80 °C for 48 h.

**Scheme 25:**
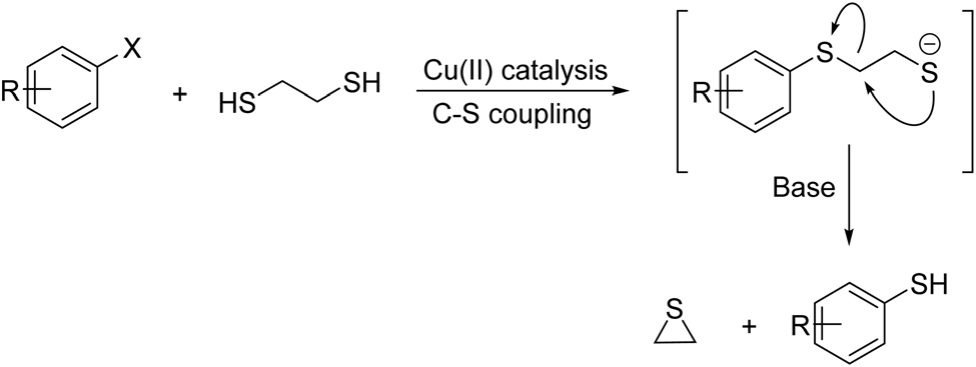
Ethanedithiol as a sulfur source.

An efficient one-step process for transforming aryl halides into thiols, which avoids an additional hydrolysis or reduction step, was developed by Chae.^203^ This method relies on 1,2-ethanedithiol to act as the sulfur source, generating an alkyl aryl sulfide, which then undergoes intramolecular S_N_2 attack to cleave the C–S bond, liberating the thiol product (Scheme 25).

Optimisation studies confirmed that CuSO_4_·5H_2_O and ethanedithiol in DMSO/water afforded the best yields. Aryl iodides were readily transformed in mostly excellent yield irrespective of electronic or steric effects (Table 58). Bromide substrates typically required elevated temperatures. Aryl thiols, which are often difficult to obtain *via* other routes, were obtained in good to excellent yields when caesium carbonate was used *in lieu* of potassium hydroxide to minimise hydrolysis of sensitive functional groups (entries 19–32, 41–43). Dihalogenated substrates (entries 36–38) were converted to their respective monothiols with substitution of the most reactive halogen in each case. Conversion of substrates bearing strongly electron-withdrawing groups proceeded in moderate to good yields in the absence of the catalyst (entries 39–43), although yields were noticeably improved upon catalyst addition.

**Table 58:** Direct synthesis of aryl thiols from aryl halides using 1,2-ethanedithiol

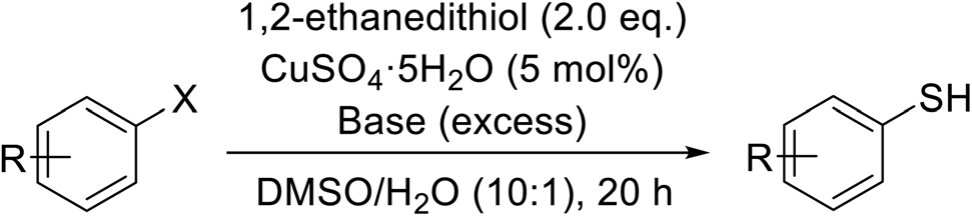					
Entry	Substrate		Base	Temp. (°C)	Yield
	R	X			
1	4-Me	I	KOH (5 eq.)	90	97%
2	3,5-Me	I	KOH (5 eq.)	90	91%
3	2-Me	I	KOH (5 eq.)	90	96%
4	4-Ph	I	KOH (5 eq.)	90	99%
5	4-Ph	Br	KOH (5 eq.)	110	85%
6	1-Naphthyl	I	KOH (5 eq.)	90	98%
7	1-Naphthyl	Br	KOH (5 eq.)	110	92%
8	2-Naphthyl	I	KOH (5 eq.)	90	92%
9	2-Naphthyl	Br	KOH (5 eq.)	110	81%
10	4-H_2_N	I	KOH (5 eq.)	90	92%
11	2-H_2_N	I	KOH (5 eq.)	90	99%
12^a^	4-HO	I	KOH (6 eq.)	90	93%
13	3-HO	I	KOH (6 eq.)	90	90%
14	2-HO	I	KOH (6 eq.)	90	95%
15	4-HO(Me)CH	I	KOH (5 eq.)	90	97%
16	4-MeO	I	KOH (5 eq.)	90	88%
17	2-MeO	I	KOH (5 eq.)	90	93%
18	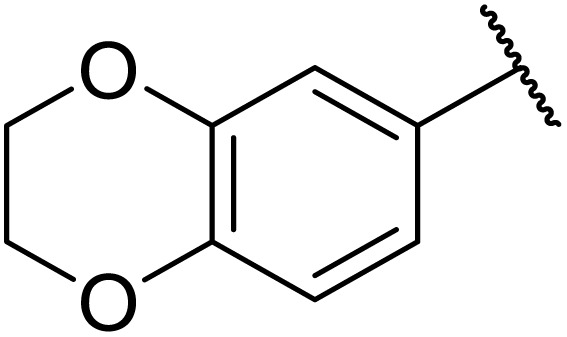	I	KOH (5 eq.)	90	89%
19	4-Ac	I	Cs_2_CO_3_ (5 eq.)^b^	90	95%
20	4-Ac	Br	Cs_2_CO_3_ (5 eq.)^b^	110	87%
21	3-Ac	I	Cs_2_CO_3_ (5 eq.)^b^	90	84%
22	3-Ac	Br	Cs_2_CO_3_ (5 eq.)^b^	110	82%
23	2-Ac	I	Cs_2_CO_3_ (5 eq.)^b^	90	80%
24	2-Ac	Br	Cs_2_CO_3_ (5 eq.)^b^	110	79%
25	2-Ac	Cl	Cs_2_CO_3_ (5 eq.)^b^	110	80%
26	4-HO_2_C	I	Cs_2_CO_3_ (5 eq.)^b^	90	91%
27	4-HO_2_C	Br	Cs_2_CO_3_ (5 eq.)^b^	110	93%
28	4-EtO_2_C	I	Cs_2_CO_3_ (5 eq.)^b^	90	85%
29	4-AcHN	I	Cs_2_CO_3_ (5 eq.)^b^	90	91%
30	4-NC	I	Cs_2_CO_3_ (5 eq.)^b^	90	93%
31	4-NC	Br	Cs_2_CO_3_ (5 eq.)^b^	110	87%
32	3-NC	Br	Cs_2_CO_3_ (5 eq.)^b^	110	95%
33	4-HO_2_C	I	KOH (6 eq.)	90	94%
34	4-HO_2_C	Br	KOH (6 eq.)	110	91%
35	3-HO_2_C	I	KOH (6 eq.)	90	94%
36	4-Br	I	KOH (5 eq.)	90	88%
37	2-Br	I	KOH (5 eq.)	90	91%
38	3-Cl	Br	KOH (5 eq.)	110	92%
39	2-F_3_C	Br	KOH (5 eq.)	90	87% (c75%)
40	2-F_3_C	Cl	KOH (5 eq.)	90	91% (c68%)
41	4-O_2_N	I	Cs_2_CO_3_ (5 eq.)^b^	60	95% (c95%)
42	4-O_2_N	Br	Cs_2_CO_3_ (5 eq.)^b^	60	92% (c93%)
43	4-O_2_N	Cl	Cs_2_CO_3_ (5 eq.)^b^	60	94% (c83%)
44	2-Quinolyl	Br	KOH (5 eq.)	110	94%
45	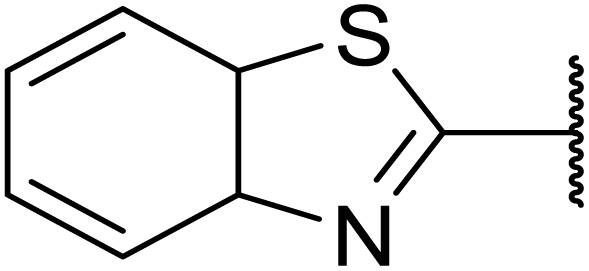	Br	KOH (5 eq.)	110	93%
46	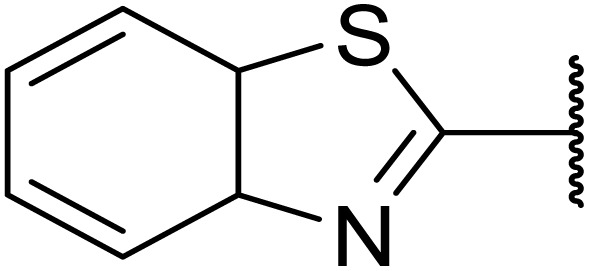	Cl	KOH (5 eq.)	110	84%

a Reaction time of 30 h.

b DMSO only.

c Yield obtained in absence of Cu(ii) catalyst.

The same group subsequently demonstrated how sodium sulfide and a catalytic amount of ethanedithiol could effect the same transformation without the requirement for a base.^198^ Surprisingly, copper powder was found to outperform both Cu(i)- and Cu(ii)-derived salts. This methodology was restricted to aryl iodides only, with good to excellent yields obtained in most cases (Table 59). Substrates possessing alkyl or aryl substituents afforded the corresponding thiols in excellent yields (entries 1–5). Electron-rich starting materials (entries 6–8, 10) returned excellent yields apart from 4-iodoaniline where the amphoteric product was difficult to isolate (entry 9). Thiolation of electron-poor substrates was similarly successful (entries 11–14) although lower yields were recorded for heteroaryl iodides (entry 15). The major reaction pathway mirrors Scheme 25 and involves Cu-catalysed coupling of the aryl iodide and 1,2-ethanedithiol, followed by intramolecular sulfide cleavage to afford the aryl thiol and thiirane by-product. Thiirane then reacts with sodium sulfide to regenerate the dithiolate.

**Table 59:** Thiolation of aryl iodides using sodium sulfide and catalytic 1,2-ethanedithiol

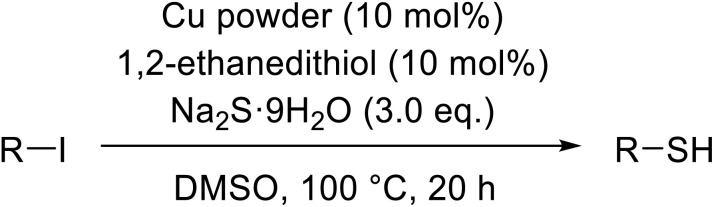		
Entry	R	Yield
1	4-Toluyl	97%
2	2-Toluyl	88%
3	3,5-Me_2_C_6_H_3_	96%
4	4-Ph	97%
5	1-Naphthyl	97%
6	2-MeOC_6_H_4_	83%
7	3-MeOC_6_H_4_	89%
8	4-MeOC_6_H_4_	93%
9	4-H_2_NC_6_H_4_	77%
10	3-HOC_6_H_4_	96%
11	4-ClC_6_H_4_	95%
12	4-AcC_6_H_4_	96%
13	3-HO_2_CC_6_H_4_	92%
14	4-HO_2_CC_6_H_4_	99%
15	2-Pyridyl	76%

### 4.2 Palladium catalysis

Palladium catalysis is a lynchpin in synthetic chemistry and offers nearly endless possibilities for C–C bond formation.^204^ Palladium-catalysed reactions have also dominated C–X bond formation due to their versatility, functional group tolerance, efficiency,^204^ and low catalyst loadings.^205,206^ Palladium-catalysed aryl halide to thiol conversions are applicable to a broader (pseudo)halide substrate scope than their copper catalyst counterparts.^204^

A Pd-catalysed coupling of aryl chlorides, bromides and triflates with thiosulfate has been developed by Yi and colleagues (Table 60).^205^ Initial screening proved that 1 mol% Pd_2_(dba)_3_ with 4 mol% 2-dicyclohexyl-phosphino-2′,4′,6′-tri-i-propyl-1,1′-biphenyl (XPhos) as the ligand and caesium carbonate was optimal. *In situ* reduction with zinc powder and hydrochloric acid furnished the target thiols in high yields (entries 1–9). For aryl chlorides (entries 11–14) and triflates (entries 15–17), Buchwald's preactivation strategy, using a combination of 2 mol% Pd(OAc)_2_ and 3 mol% Xphos,^207^ offered improved yields (entries 10 *vs.* 11).

**Table 60:** Conversion of aryl halides and triflates to thiophenols using palladium catalysis

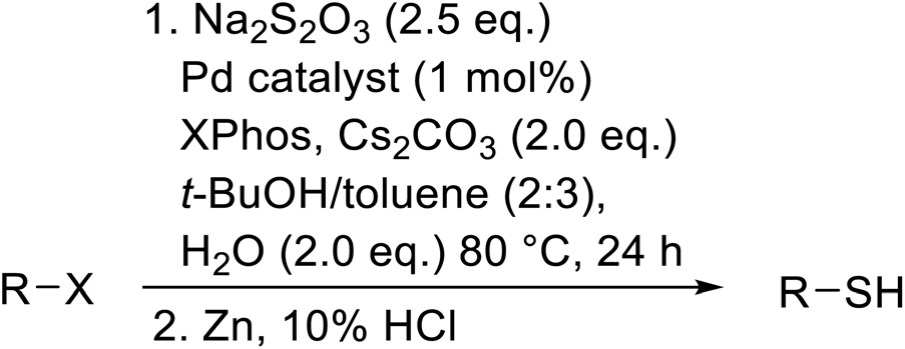					
Entry	Substrate		Pd catalyst	XPhos loading	Yield
	R	X			
1	4-Toluyl	Br	Pd_2_(dba)_3_	4 mol%	81%
2	2-Toluyl	Br	Pd_2_(dba)_3_	4 mol%	75%
3	1-Naphthyl	Br	Pd_2_(dba)_3_	4 mol%	67%
4	2-Naphthyl	Br	Pd_2_(dba)_3_	4 mol%	95%
5	4-F_3_CC_6_H_4_	Br	Pd_2_(dba)_3_	4 mol%	80%
6	4-Biphenyl	Br	Pd_2_(dba)_3_	4 mol%	90%
7	2-MeOC_6_H_4_	Br	Pd_2_(dba)_3_	4 mol%	78%
8	3-Toluyl	Br	Pd_2_(dba)_3_	4 mol%	82%
9	4-FC_6_H_4_	Br	Pd_2_(dba)_3_	4 mol%	73%
10	4-Toluyl	Cl	Pd_2_(dba)_3_	4 mol%	68%
11	4-Toluyl	Cl	Pd(OAc)_2_	3 mol%	82%
12	Ph	Cl	Pd(OAc)_2_	3 mol%	72%
13	4-F_3_CC_6_H_4_	Cl	Pd(OAc)_2_	3 mol%	85%
14	2-MeOC_6_H_4_	Cl	Pd(OAc)_2_	3 mol%	68%
15	2-Naphthyl	OTf	Pd(OAc)_2_	3 mol%	90%
16	3,5-Me_2_C_6_H_3_	OTf	Pd(OAc)_2_	3 mol%	91%
17	4-F_3_CC_6_H_4_	OTf	Pd(OAc)_2_	3 mol%	70%

**Scheme 26:**
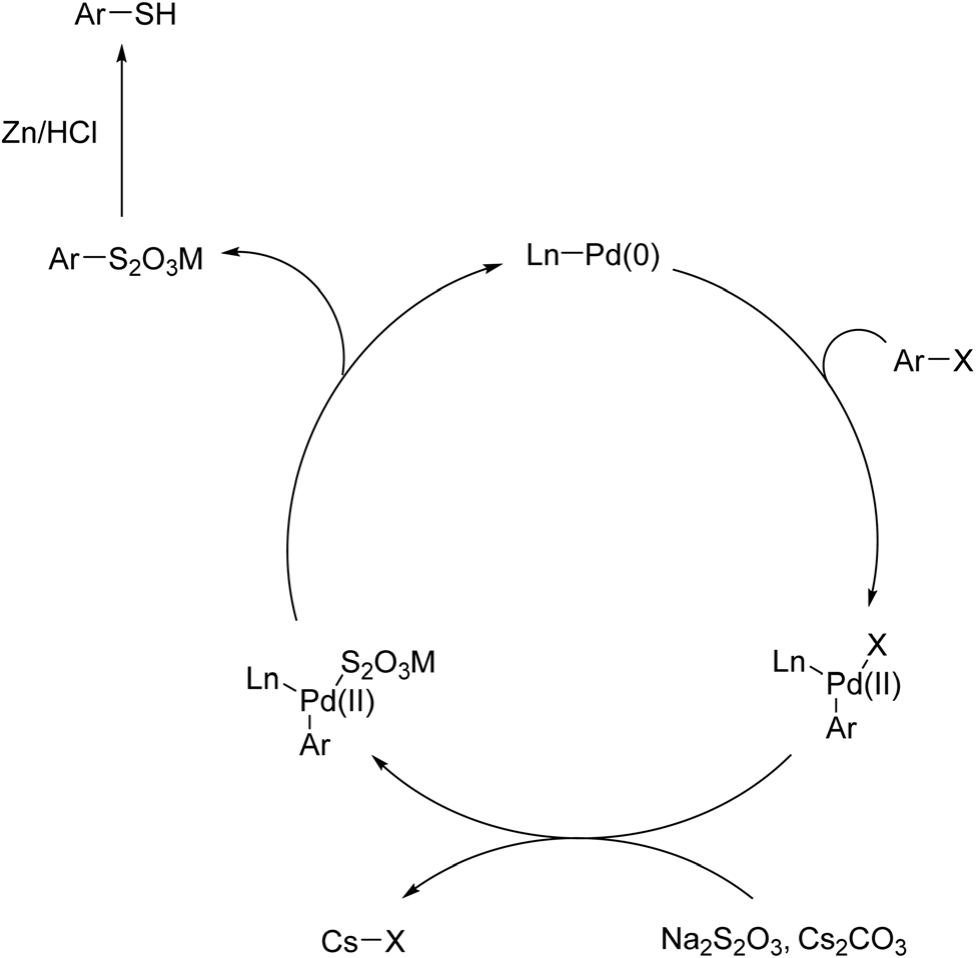
Pd-mediated coupling of thiosulfates.

The proposed mechanism is outlined in Scheme 26. Oxidative addition of R–X to Pd(0) is followed by anion exchange between the Pd(ii) halide intermediate and thiosulfate to generate the Pd(ii) thiosulfate intermediate. Reductive elimination regenerates Pd(0) and affords the aryl thiosulfate intermediate. Reduction of the intermediate with zinc powder and hydrochloric acid affords the thiol product.

Van Den Hoogenband *et al.* successfully prepared several *S*-aryl thioacetates as thiol surrogates (Table 61).^206^ Inspired by Hartwig's prior work on the thioetherifcation of aryl halides, a catalyst system of Pd_2_(dba)_3_ and (*R*)-1-[(S_p_)-2-(dicyclohexylphosphino)ferrocenyl]ethyldi-*tert*-butylphosphine (CyPF-*t*-Bu) in toluene at 110 °C promoted the coupling of thioacetate with aryl bromides (entries 1–9, 11, 14) and triflates (entries 10, 12, 15–18).^208^ In general, aryl bromides were more reactive than triflates (entry 9 *vs.* 10, 11 *vs.* 12), while coupling of aryl chlorides was possible but in depressed yields (entry 13). The authors note that the thioacetate surrogates may be converted to their corresponding thiols *via* basic hydrolysis.

**Table 61:** Preparation of *S*-arylthioacetates as aryl thiol surrogates

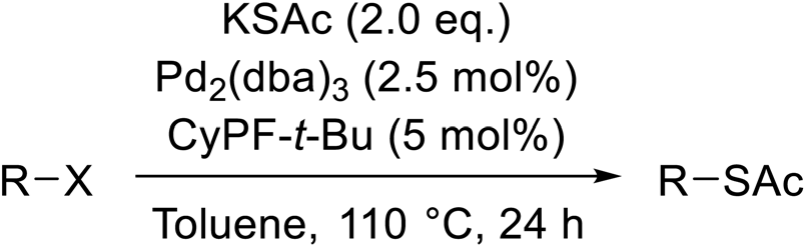			
Entry	Substrate		Yield (*S*-aryl thioacetate)
	R	X	
1	3-Quinolyl	Br	90%
2	3-Pyridyl	Br	80%
3	4-H_2_NC_6_H_4_	Br	60%
4	4-AcHNC_6_H_4_	Br	72%
5	4-CbzHNC_6_H_4_	Br	55%
6	4-HCOHNC_6_H_4_	Br	90%
7	4-PhCOC_6_H_4_	Br	70%
8	4-O_2_NC_6_H_4_	Br	15%
9	3-O_2_NC_6_H_4_	Br	13%
10	3-O_2_NC_6_H_4_	OTf	0%
11	4-EtO_2_CC_6_H_4_	Br	67%
12	4-EtO_2_CC_6_H_4_	OTf	58%
13	4-EtO_2_CC_6_H_4_	Cl	36%
14	4-MeOC_6_H_4_	Br	20%
15	5-Indolyl	OTf	16%
16	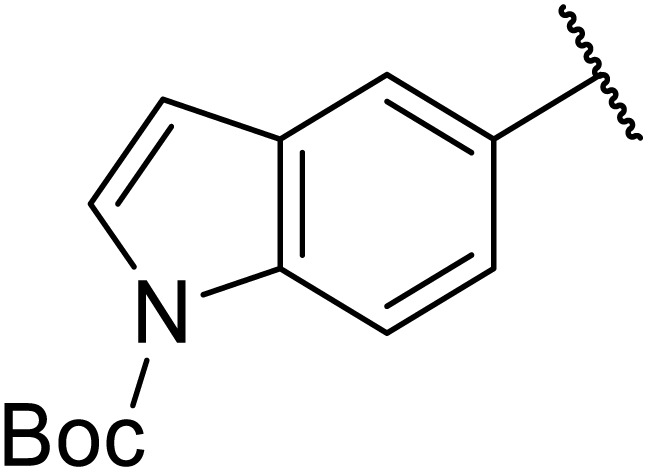	OTf	79%
17	6-Quinolyl	OTf	69%
18	4-AcC_6_H_4_	OTf	67%

### 4.3 Nickel catalysis

For aryl halide to thiophenol transformations, nickel appears to be at least as efficient as copper, with typically excellent yields. An advantage of nickel catalysis is the low catalyst loadings (<1 mol%) required to effect transformations. Nickel is also less toxic than palladium, although it is more harmful than copper and is a common culprit for causing allergic contact dermatitis.^209^ Nickel-catalysed cross-coupling of aryl iodides is compatible with highly functionalised and labile substrates, as has been demonstrated in the synthesis of known Active Pharmaceutical Ingredients.^210^

The ability of nickel catalysis to facilitate C–S cross-coupling reactions between aryl halides and thiols to afford aryl sulfides is well established,^211,212^ with an early example reported by Takagi.^213^ A Ni(0) catalyst was generated *in situ* from bis(triethylphosphine)Ni(ii) chloride and sodium cyanoborohydride which facilitated the S_N_Ar reaction of aryl iodides with thiourea to form *S*-isothiouronium iodide salts.^213^ Low catalyst (0.8 mol%) and reductant (1.2 mol%) loadings were sufficient to catalyse the reactions and afford the *S*-isothiouronium salts in good to excellent yields (Table 62). The intermediate salts were easily converted to aryl thiols by aqueous hydrolysis. Polar solvents were preferable, as non-polar solvents such as benzene led to the precipitation of nickel and diminished yields (entry 6). Electron-donating groups had little effect on the reactivity of the aryl iodide substrates (entries 9, 10). While bromobenzene did not react under these conditions, dithiol formation was observed with 1-bromo-4-iodobenzene, suggesting that the isothiouronium group increases the reactivity of aryl halides (entry 12).

**Table 62:** Synthesis of aryl thiols from aryl halides and thiourea under nickel catalysis

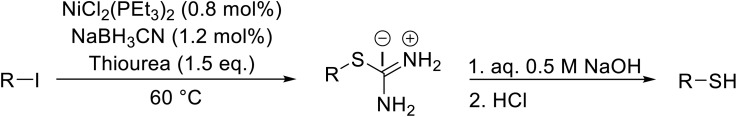				
Entry	R	Solvent	Time (h)	Yield
1	Ph	DMF	3	98%
2^a^	Ph	DMF	25	98%
3	Ph	MeCN	4	97%
4	Ph	Acetone	10	70%
5	Ph	1,4-Dioxane	4	92%
6	Ph	Benzene	10	16%
7	4-Toluyl	DMF	3	97%
8	2-Toluyl	DMF	10	91%
9	4-MeOC_6_H_4_	DMF	3	98%
10	4-H_2_NC_6_H_4_	DMF	3	98%
11	4-ClC_6_H_4_	DMF	3	98%
12	4-BrC_6_H_4_	DMF	24	b73%

a Reaction conducted at 40 °C.

b *para*-Benzenedithiol was obtained in 20% yield.

Building upon Takagi's work, Ball and Magné improved the efficiency of the original methodology and expanded the substrate scope (Table 63).^210^ Optimisation studies confirmed that a 0.8 mol% loading of commercially available bis(triphenylphosphine)nickel(ii) dichloride with picoline-borane as reductant in an anaerobic atmosphere was optimal. Due to difficulties in crystallising isothiouronium iodide salts from NMP, they were instead converted to the corresponding isothiouronium 3,5-dinitrobenzoate (DNB) salts and isolated by filtration. The thiols could be obtained by subsequent basic hydrolysis in near-quantitative yields. Aryl substrates with electron-donating (entries 1–5) or electron-withdrawing (entries 7–16) groups were successfully transformed. Reactions involving *ortho*-substituted aryl iodides were generally slower (entries 17–20, 22). The conversion of anilines (entry 23) and phenols (entry 24) proceeded smoothly as did the reaction of base and nucleophile-sensitive substrates (entries 32–35). Both polyfunctional (entries 38–41) and heteroaryl substrates (entries 42–54) were likewise compatible with these conditions. The versatility of this approach is exemplified by the preparation of the pharmaceutically relevant precursor to celecoxib (entry 38), an important non-steroidal anti-inflammatory drug.^214^

**Table 63:** Synthesis of *S*-isothiouronium-DNB salts as thiol surrogates from aryl halides and thiourea

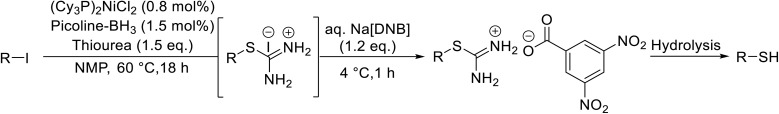					
Entry	R	Yield (salt)	Entry	R	Yield (salt)
1	4-Et_2_NC_6_H_4_	65%	2	4-MeOC_6_H_4_	91%
3	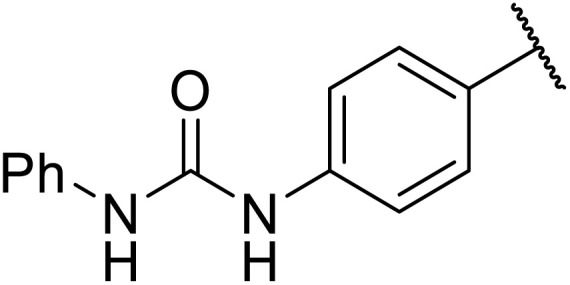	97%	4	4-Toluyl	69%
5	3-EtC_6_H_4_	90%	6	Ph	84% (a69%)
7	4-FC_6_H_4_	96% (b92%)	8	4-ClC_6_H_4_	97%
9	4-F_3_COC_6_H_4_	88%	10	4-AcC_6_H_4_	95%
11	3-AcC_6_H_4_	98%	12	4-PhOCC_6_H_4_	90%
13	4-MeO_2_CC_6_H_4_	88% (a95%)	14	4-F_3_CC_6_H_4_	74%
15	4-NCC_6_H_4_	83%	16	3-NCC_6_H_4_	91%
17	2,4-Me_2_C_6_H_3_	65% (a93%)	18	2-Me-4-FC_6_H_3_	73%
19	2-MeOC_6_H_4_	77%	20	2-EtC_6_H_4_	62%
21	1-Naphthyl	94%	22	2-ClC_6_H_4_	36%
23	4-H_2_NC_6_H_4_	83%	24	4-HOC_6_H_4_	98%
25	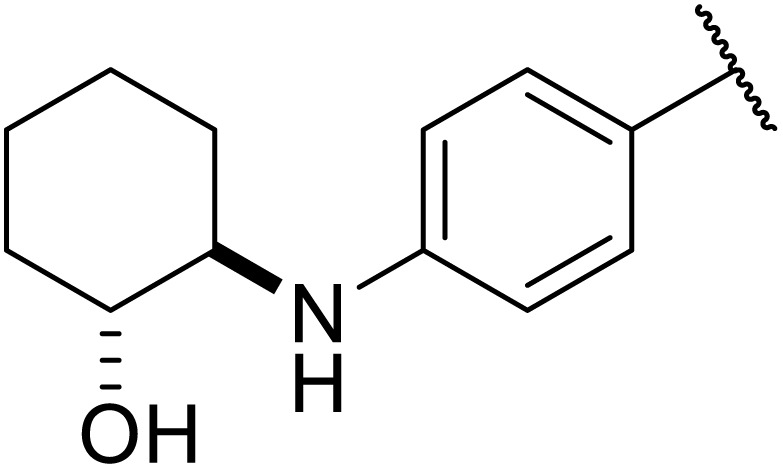	70%	26	4-H_2_NOCC_6_H_4_	97% (a97%)
27	4-H_2_NO_2_SC_6_H_4_	82%	28	3-HO_2_CC_6_H_4_	82%
29	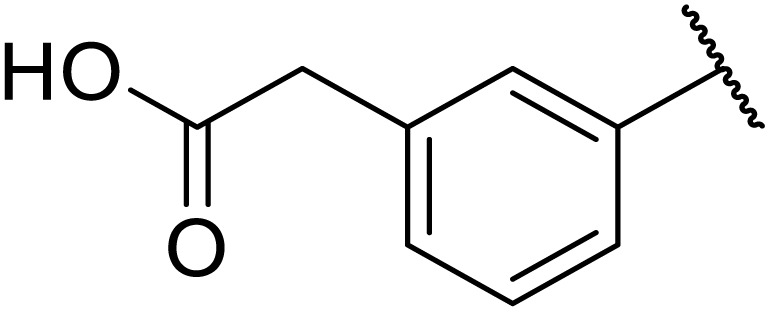	86%	30	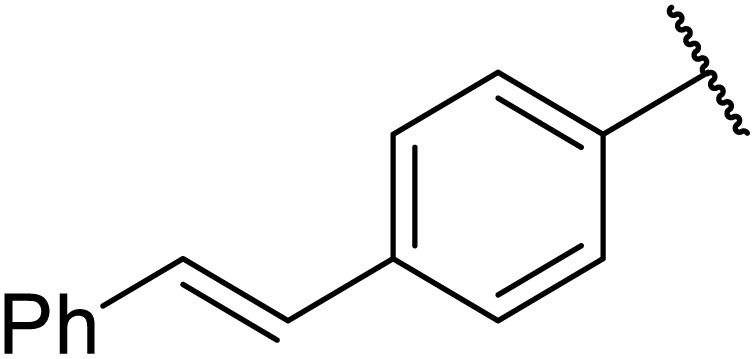	86%
31	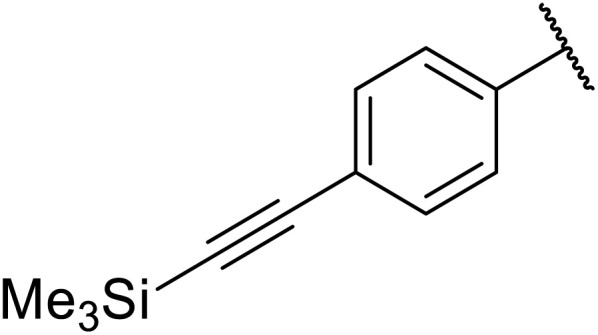	59%	32	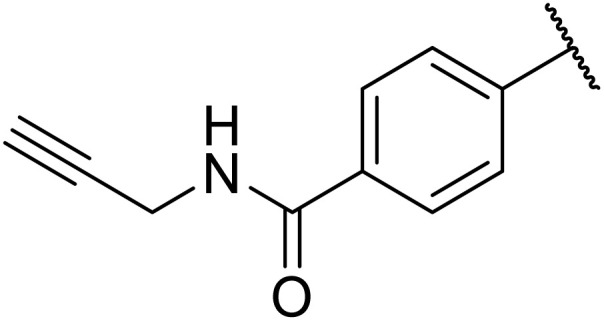	75%
33	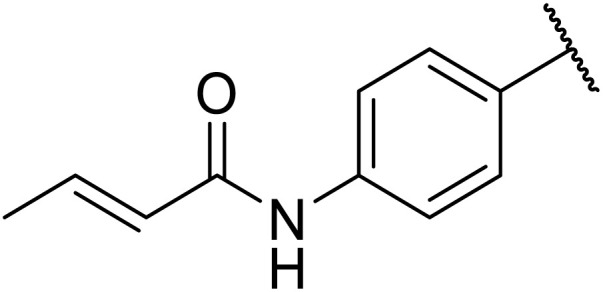	81%	34	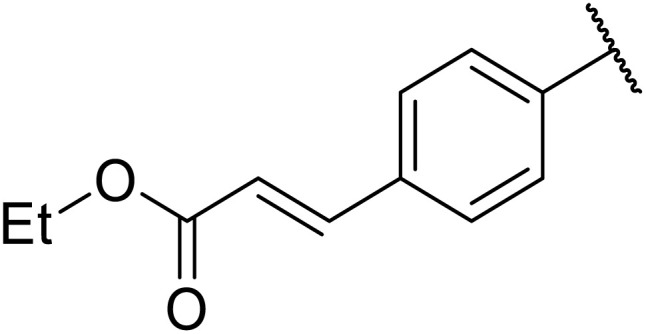	91%
35	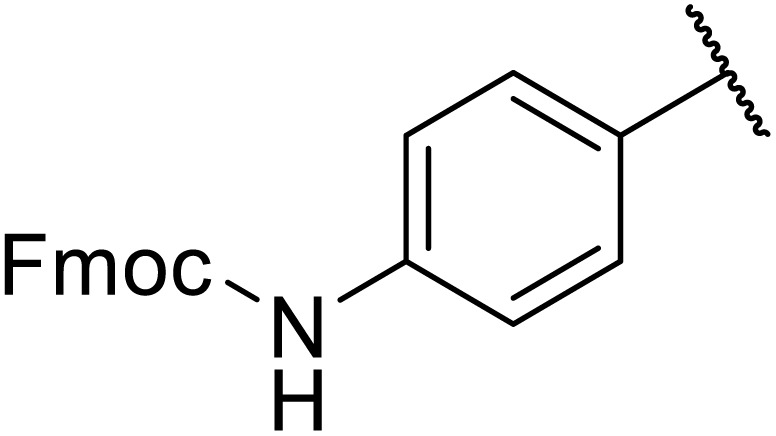	97%	36	4-B(pin)C_6_H_4_	84%
37	3-TsOC_6_H_4_	92%	38	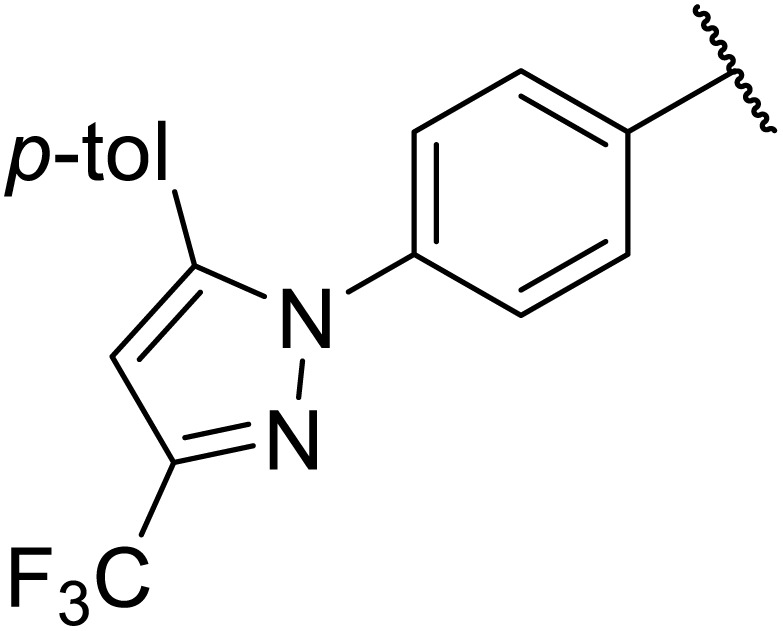	74%
39	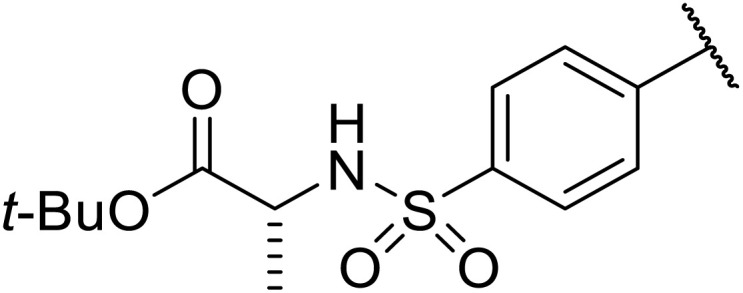	78%	40	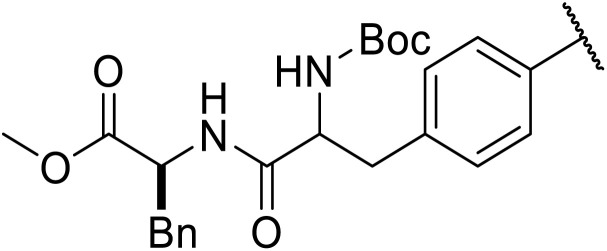	82%
41	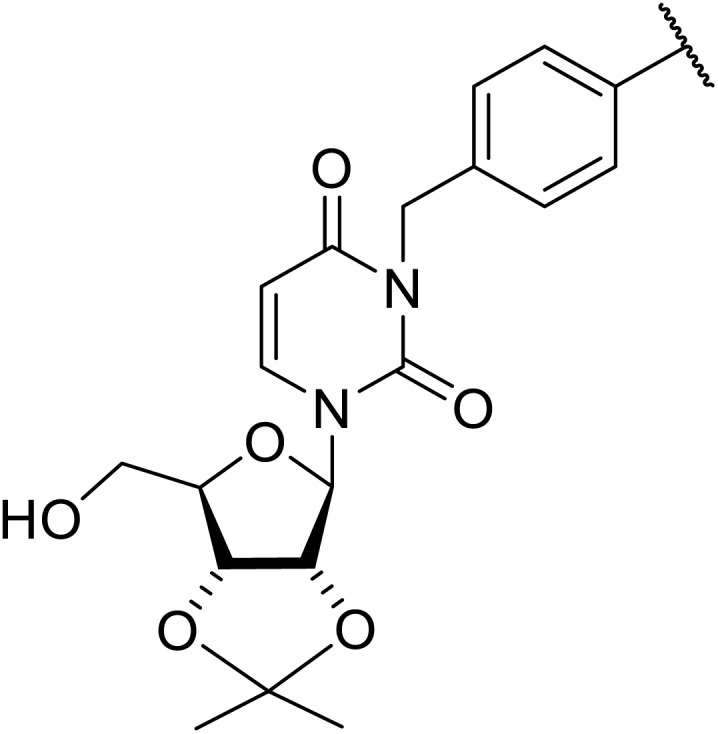	54%	42	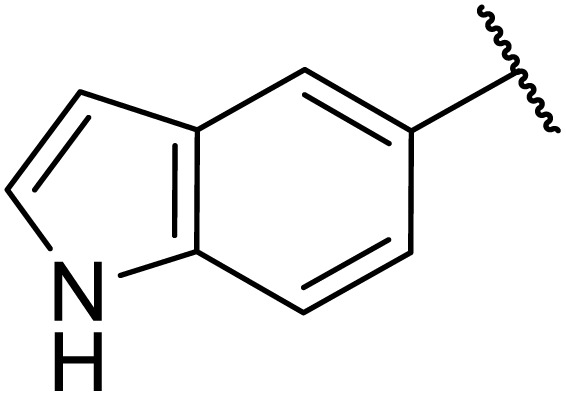	93%
43	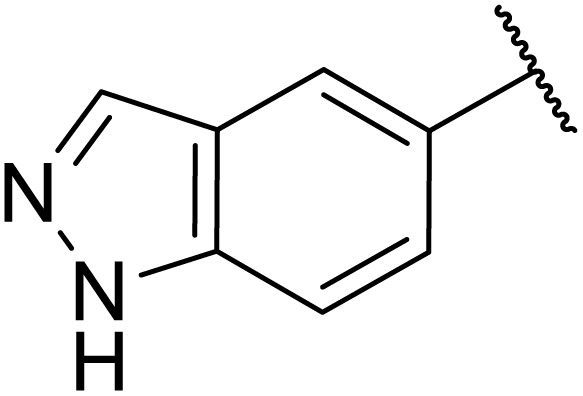	83%	44	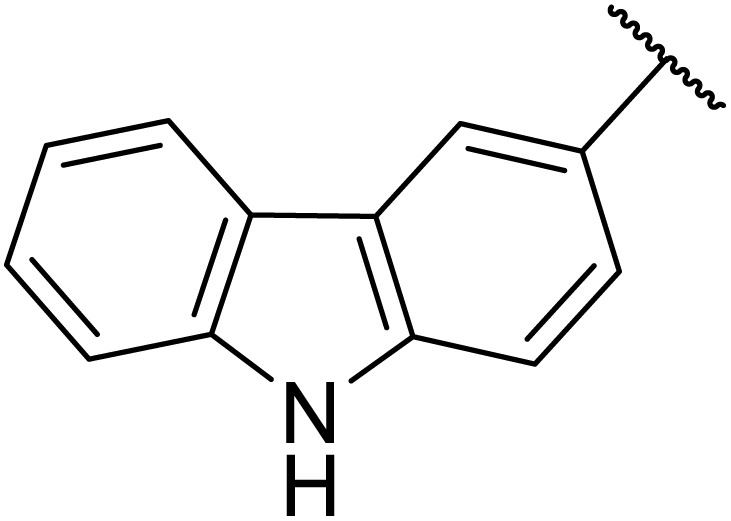	99%
45	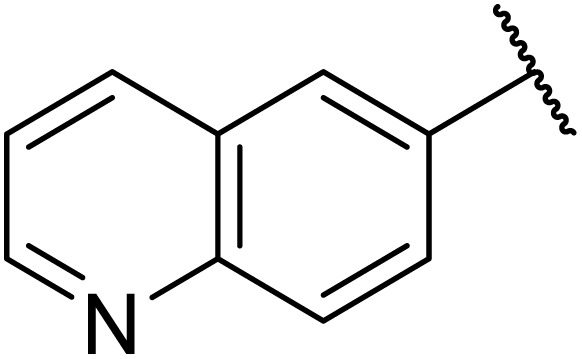	91%	46	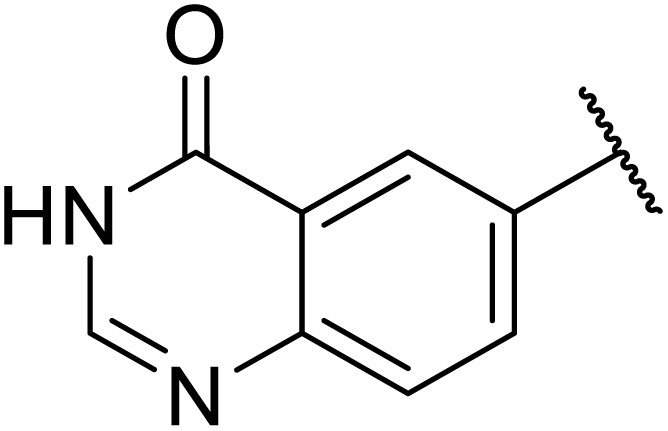	99%
47	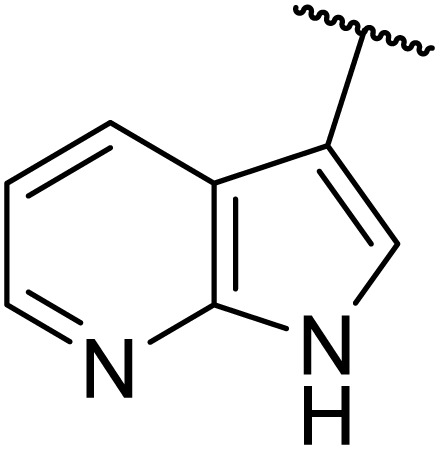	67%	48	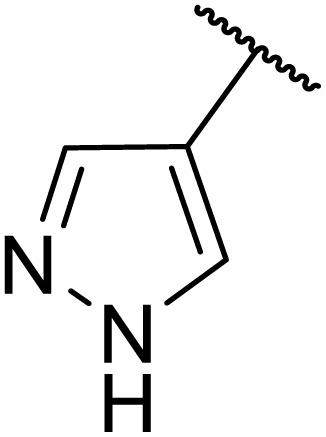	20%
49	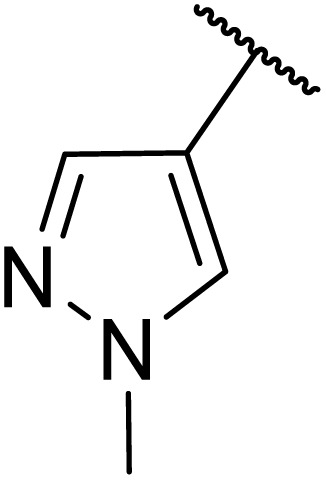	68%	50	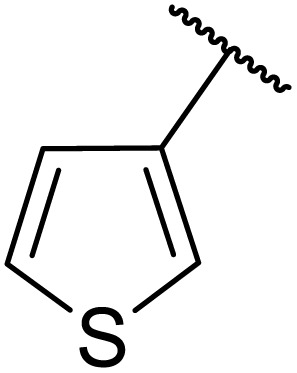	76%
51	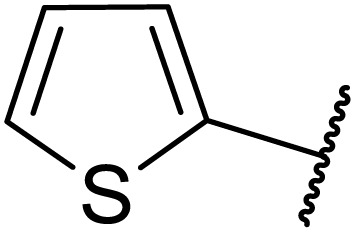	40%	52	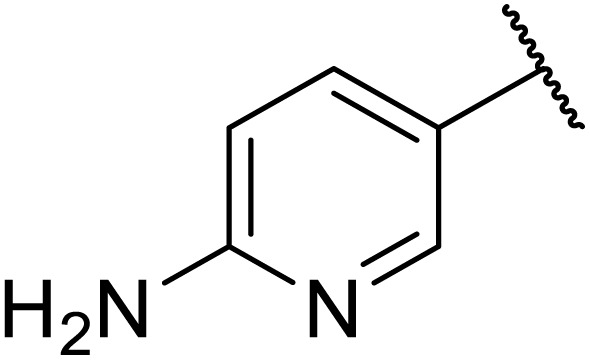	65%
53	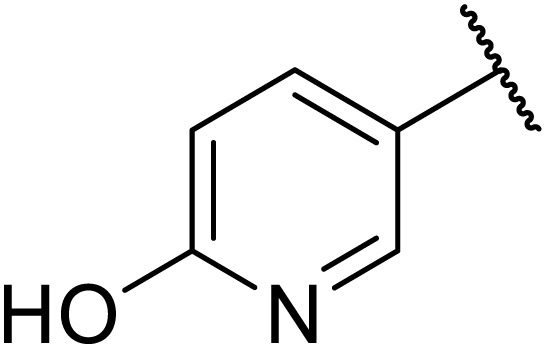	70% (a68%)	54	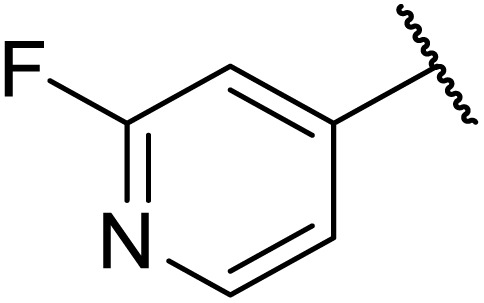	78%

a Isolated yield of the *S*-isothiouronium-DNB salt at 10-fold scale increase.

b Isolated yield of the *S*-isothiouronium-DNB salt at 100-fold scale increase.

### 4.4 Miscellaneous methods

This section includes a variety of reagents and/or methods for converting aryl halides to thiols without recourse to transition metal catalysts. Of these methods, the most synthetically relevant employ alkanethiolate salts which have proven effective at converting unactivated aryl halides.^215–217^

Tiecco *et al.* developed a one-pot synthesis of aryl thiols using sodium methanethiolate in HMPA.^215^ A range of unactivated aryl halides were successfully thiolated in high yields (Table 64, entries 1–7). Initial nucleophilic aromatic substitution generates the aryl methylsulfide, followed by attack of the thiolate to afford the thiol upon acidification. The first step is generally faster than the second, facilitating access to aryl methyl sulfides in high yields.

**Table 64:** One-pot thiolation of unactivated aryl halides

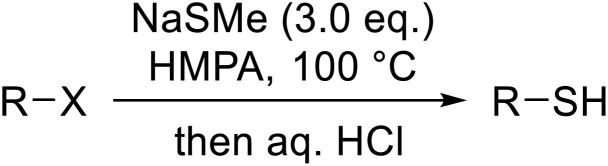				
Entry	R	X	Time (h)	Yield
1	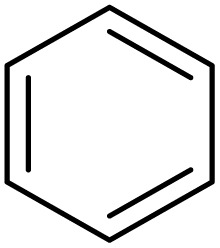	Cl	5	90%
2	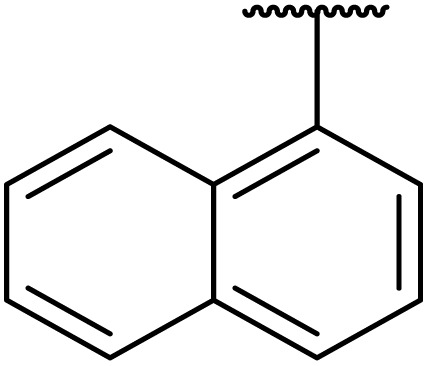	Br	3.5	90%
3	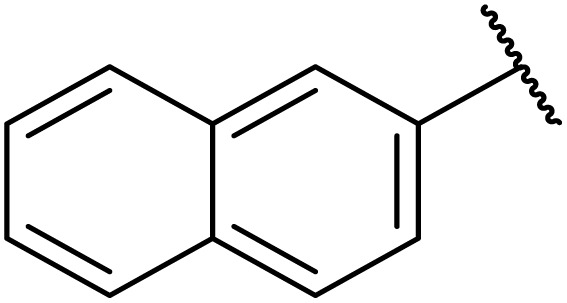	Br	5	97%
4	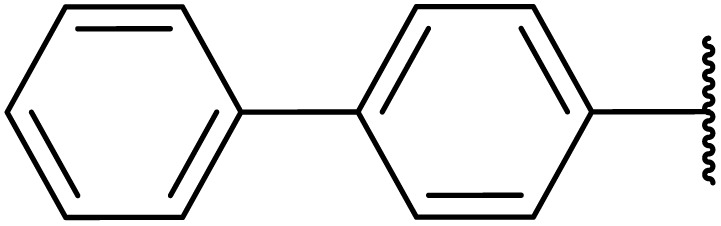	Br	2	96%
5	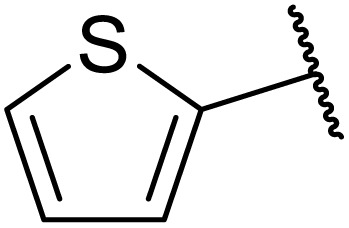	Br	2	88%
6	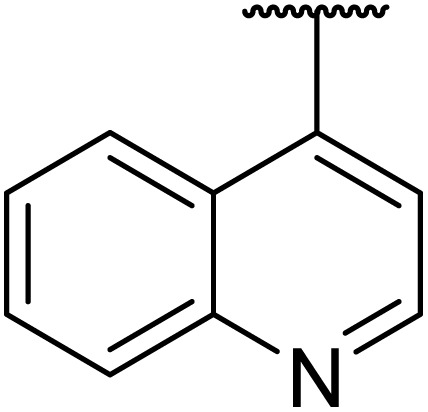	Cl	25	80%
7	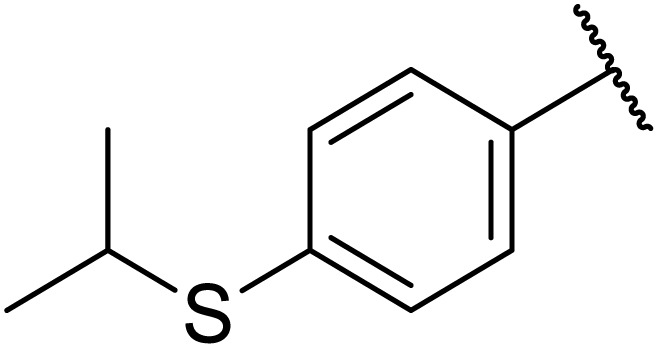	Cl	2	90%

The same authors later modified the conditions to replace carcinogenic HMPA with DMF and swapped sodium methanethiolate with easier-to-handle sodium ethanethiolate (Table 65).^216^ These improved conditions were compatible with both aryl (entries 1–7) and heteroaryl (entries 8–10) substrates. Thiolation of polyhalogenated rings was also successful (entries 6, 7).

**Table 65:** Thiolation of hetero/aryl halides using sodium ethanethiolate in DMF

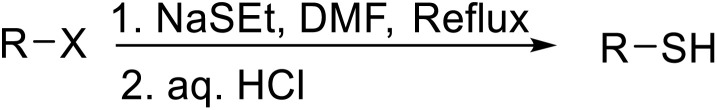						
Entry	Substrate		NaSEt (eq.)	Time (h)	Product	
	R	X			R	Yield
1	4-Ph	Cl	10	23	4-Ph	78%
2	4-Biphenyl	Br	6	22	4-Biphenyl	67%
3	1-Naphthyl	Br	6	22	1-Naphthyl	95%
4	2-Naphthyl	Br	5	4	2-Naphthyl	94%
5	4-i-PrSC_6_H_4_	Cl	6	24	4-i-PrSC_6_H_4_	a95%
6	4-ClC_6_H_4_	Cl	5	17	4-EtSC_6_H_4_	a80%
7	2,4,5-Cl_3_C_6_H_2_	Cl	8	1	2,4,5-(EtS)_3_C_6_H_2_	75%
8	4-Pyridyl	Cl	5	16	4-Pyridyl	a70%
9	2-Quinolyl	Cl	7	7	2-Quinolyl	a81%
10	4-Quinolyl	Cl	5	6	4-Quinolyl	80%

a Product isolated as the methyl sulfide following MeI addition.

In a related study, Shaw obtained superior yields by substituting DMF for NMP.^217^ Reaction of aryl chlorides with either sodium propanethiolate or butanethiolate produced the target thiols in high yields as measured by gas chromatography (Table 66, entries 1–5). By contrast, branched alkyl thiolates returned low yields due to increased steric hindrance (entry 6).

**Table 66:** Thiolation of aryl chlorides using thiolate salts in NMP

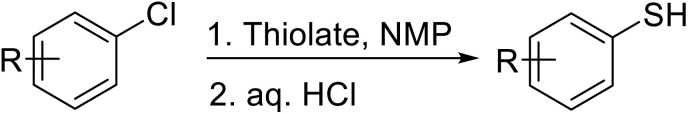						
Entry	Substrate	Thiolate	Temp. (°C)	Time (h)	Product	
	R				R	Yield
1	4-Me	*n*-PrSNa	186	20	4-Me	87%
2	4-Me	*n*-BuSNa	186	20	4-Me	90%
3	2-Me	*n*-BuSNa	186	20	2-Me	96%
4	H	*n*-PrSNa	172	9	H	92%
5	4-Cl	*n*-BuSNa	185–190	9	*n*-BuS	94%
6	4-Cl	*t*-BuSNa	150	6.5	*t*-BuS	7%

Thiébault and colleagues employed electrochemical means for preparing thiophenols from aryl chlorides.^193^ When current was applied to a combination of aryl chloride, thiourea and potassium *tert*-butoxide in liquid ammonia in the presence of potassium bromide electrolyte and a mediator, the target thiols were generated in low to good yields (Table 67, entries 1–5). Substrates capable of producing more reactive radicals returned higher yields (entries 1–2), whereas 2-pyridyl, chlorophenyl and pyridinophenyl radicals were associated with poorer outcomes (entries 3–5). The reaction proceeds *via* an S_RN_1 mechanism, where the aryl chloride is reduced to its radical, and then combines with thiourea anion to form a C–S-coupled anion-radical. This anion-radical undergoes cleavage to produce a thiophenoxide, ultimately leading to the target thiol.

**Table 67:** Electrochemical synthesis of aryl thiols from aryl chlorides

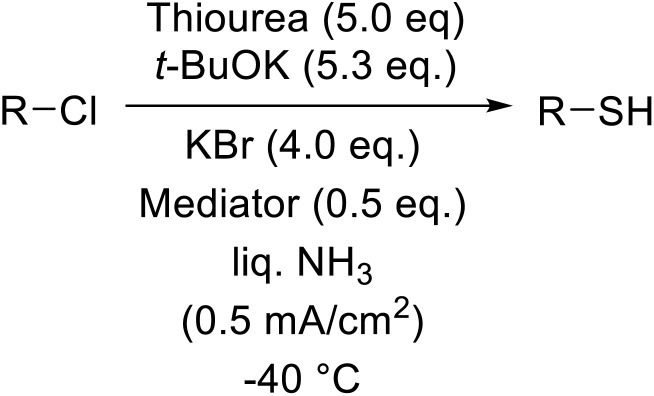			
Entry	R	Mediator	Yield
1	4-NCC_6_H_4_	4,4′-dipyridyl	34%
2	4-Pyridyl	2,4′-dipyridyl	75%
3^a^	2-Pyridyl	2,2′-dipyridyl	23%
4^b^	2-ClC_6_H_4_	Benzonitrile	16%
5	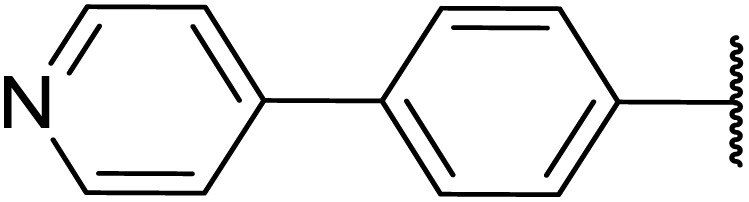	4,4′-dipyridyl	32%

a Yield determined by HPLC.

b Thiophenol obtained as the product.

**Scheme 27:**
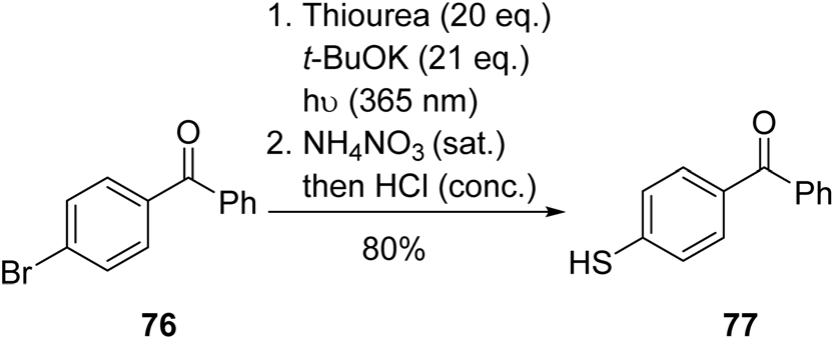
Photochemical coupling of thiourea.

In a similar vein, Peñéñory and colleagues employed photochemical means to couple aryl halides and thiourea.^192^ Treatment of a large excess of thiourea with base generated the anion which underwent photoinduced coupling to the aryl halide at 365 nm. Following acidic hydrolysis, the resulting thiolate intermediates were typically quenched with methyl iodide to produce aryl sulfides, rather than thiols. In one instance, however, protonation of the thiolate intermediate afforded aryl thiol 77 in 80% yield (Scheme 27). Addition of di-*tert*-butyl nitroxide (DTBN) inhibited the reaction, confirming a radical mechanism.

The main characteristics of the different approaches for preparing thiophenols from aryl halides are summarised in Table 68.

**Table 68:** Summary of approaches for the preparation of thiophenols from aryl halides

Approach	Characteristics	Advantages	Disadvantages
Copper catalysis	Cu(0), Cu(i) and Cu(ii) catalysts	Widely available; cheap; ligand-free; non-toxic	Mostly limited to ArI
Palladium catalysis	Pd(0) and Pd(ii) catalysts	Low loading; functional group tolerance; (pseudo)halide substrate diversity	Environmental pollutant; toxic; expensive
Nickel catalysis	Ni(ii) catalysts	Very low loading; cheap; excellent functional group tolerance	Mostly limited to ArI; moderately toxic
Miscellaneous methods	Transition metal-free	Suitable for ArCl; sustainability; good yields	More limited substrate pool; lower functional group tolerance

## 5. Conclusion

Alcohols and thiols are ubiquitous in nature and represent significant functional groups in synthetic and medicinal chemistry. The direct transformation of alkyl and aryl halides constitutes an attractive approach for their preparation. This review summarises the wide diversity of approaches that have been developed to effect this transformation. For both aliphatic alcohols and thiols, reactions of aliphatic halides with an oxyanion or thiolate salt are heavily represented in the literature. Although an intermediate is formed which must be in turn converted to the alcohol or thiol, these strategies remain popular due to their high yield, wide applicability to different halides and their broad functional group tolerance. Unsurprisingly, there is a major emphasis on transition metal-catalysed transformations in more recent literature, particularly for the preparation of aryl thiols. Copper, palladium and nickel have all proved to be effective, with the sustainability, toxicity profile and cost-effectiveness of the former making it especially attractive. By contrast, transition metal-catalysed methodologies for producing aliphatic alcohols are limited. Additionally, cutting-edge techniques, such as photochemistry and electrochemistry, are increasingly coming to the fore. Many of these newer methodologies offer greener alternatives to traditional routes although significant work is still required to improve yields and expand substrate scope. In summary, this review will hopefully serve as a useful overview of the current state-of-the-art to practising chemists and offer inspiration for future investigations in this field.

## Conflicts of interest

There are no conflicts of interest to declare.

## Data Availability

No primary research results, software or code have been included and no new data were generated or analysed as part of this review.
